# Germplasm Resources and Strategy for Genetic Breeding of *Lycium* Species: A Review

**DOI:** 10.3389/fpls.2022.802936

**Published:** 2022-02-11

**Authors:** Haiguang Gong, Fazal Rehman, Yun Ma, Biao A, Shaohua Zeng, Tianshun Yang, Jianguo Huang, Zhong Li, Dongpo Wu, Ying Wang

**Affiliations:** ^1^Key Laboratory of South China Agricultural Plant Molecular Analysis and Genetic Improvement, Provincial Key Laboratory of Digital Botanical Garden and Public Science, South China Botanical Garden, Chinese Academy of Sciences, Guangzhou, China; ^2^School of Life Science, Gannan Normal University, Ganzhou, China; ^3^School of Life Science, University of Chinese Academy of Sciences, Beijing, China; ^4^Agricultural Comprehensive Development Center in Ningxia Hui Autonomous Region, Yinchuan, China; ^5^Bairuiyuan Company, Yinchuan, China

**Keywords:** *Lycium* species, molecular breeding, strategy of breeding, standardization, trait detection

## Abstract

*Lycium* species (goji), belonging to Solanaceae, are widely spread in the arid to semiarid environments of Eurasia, Africa, North and South America, among which most species have affinal drug and diet functions, resulting in their potential to be a superior healthy food. However, compared with other crop species, scientific research on breeding *Lycium* species lags behind. This review systematically introduces the present germplasm resources, cytological examination and molecular-assisted breeding progress in *Lycium* species. Introduction of the distribution of *Lycium* species around the world could facilitate germplasm collection for breeding. Karyotypes of different species could provide a feasibility analysis of fertility between species. The introduction of mapping technology has discussed strategies for quantitative trait locus (QTL) mapping in *Lycium* species according to different kinds of traits. Moreover, to extend the number of traits and standardize the protocols of trait detection, we also provide 1,145 potential traits (275 agronomic and 870 metabolic) in different organs based on different reference studies on *Lycium*, tomato and other Solanaceae species. Finally, perspectives on goji breeding research are discussed and concluded. This review will provide breeders with new insights into breeding *Lycium* species.

## Introduction

Goji, the general name of the plants in the genus *Lycium*, are members of the Solanaceae family found in arid to semiarid regions of Eurasia, Africa, and North and South America. Worldwide, 31 species of *Lycium* have been reported as medicines and/or foods, such as *L. ciliatum* (medicine for digestive inflammation), *L. cinereum* (fruit as food and medicine for rheumatism and headache; root as medicine for perfume, kidney disease, and anodyne), *L. pallidum* (fruit as food; root as medicine for chickenpox and toothache), *L. richii* (fruit as food), *L. intricatum* (seed and fruit as medicine for helminthiasis and eye diseases, respectively), among which *L. chinense* and *L. barbarum* have been widely regarded as superfoods with affinal drug and dietary properties in recent years (Yao et al., 2018b). Goji (wolfberry), the fruit of *Lycium* plants, contains trace elements such as zinc, iron, calcium, and phosphorus as well as metabolic components such as flavonoids, carotenoids, and polysaccharides in red fleshy fruits with affinal drug and dietary functions (Yin and Dang, 2008; Wang C.C. et al., 2010; Wang J. et al., 2010; Qiu et al., 2014). Antioxidative compounds, polysaccharides (Dahech et al., 2013), and hydroxycinnamic acid amides (HCCAs) provide potential anti-inflammatory effects (Wang S. et al., 2017). *Lycium barbarum* polysaccharides (LBPs) have neuroprotective (Xing et al., 2016), liver-protective (Liu et al., 2015), anti-radiation (Duan et al., 2015), anti-fatigue (Reeve et al., 2010), antitumor (Peifei et al., 2015) and anti-aging properties (Ye et al., 2015), and they scavenge free radicals (Mocan et al., 2014), boost immunity (Su et al., 2014), reduce ischemia/reperfusion injury (Lu and Zhao, 2010), protect against cardiac poisoning (Xin et al., 2007, 2011), and improve reproductive function (Qian and Yu, 2016). Goji (*Lycium* Linn) is a significant medicinal plant, part of which goji fruit (gouqizi) and root (digupi) are two of the most important Chinese medicinal materials for diabetes prevention and treatment. Goji leaf tea, on the other hand, has a hypoglycemic effect on diabetes (Sun et al., 2018).

China is, in fact, the world’s largest producer and exporter. Goji production areas in China are estimated at 88,000 ha, covering the entire northwest to central China, including Xinjiang, Ningxia, Gansu, Qinghai, Shanxi, Inner Mongolia and Hubei (Chen et al., 2018). The yield of dry goji berries in China reached 410,608 t in 2017, while the yield in the Ningxia region, which is considered the largest goji planting area, is expected to be approximately 108,473 t (State Forestry Administration of China [SFAC], 2018). As domestic and worldwide market demands grow, the manufacturing capacity of Ningxia Province cannot keep pace (Yao et al., 2018b). Thus, it is necessary to increase goji berry production in more favorable cultivation areas (Cao and Wu, 2015; Gong et al., 2019). Furthermore, all of the cultivars used in the goji industry are for dry fruit cultivation in Ningxia Province, China, and are rarely used for other purposes. The goji industry’s constraint is a lack of cultivars for other uses (Nan et al., 2017). As a result, new cultivars with high production yield and for other uses in the goji industry must be developed.

Starting from the genetic origin of *Lycium* fruit, continuously researching and developing improved new varieties on the basis of the original varieties of *Lycium* species and improving the breeding technology system of good varieties of *Lycium* are effective methods to overcoming this bottleneck (Shi et al., 2016). However, there are few reports on the molecular markers and regulatory genes related to edible traits such as the fruit size, shape and hardness of *Lycium* species. With the development of the social economy, consumer demand for wolfberry has increased and includes products for medicinal uses, fresh food, processing, fruits, and vegetables. As such, traditional wolfberry varieties and industrial models have been unable to meet the constantly rich market demand; thus, wolfberry breeding work will be toward selection research on special types of wolfberry varieties to breed and the specificity to meet diverse usage requirements in industry (Nan et al., 2017).

In terms of breeding methods, the same problems that exist in the selection of special cultivars and ordinary cultivars of traditional *Lycium* breeding methods, especially individual plant selection methods, have been emphasized, while biological technology breeding methods have been neglected. With the development of biotechnology, molecular marker-assisted selection (MAS) breeding has been widely used in crop breeding. For example, researchers cloned for the first time a high-yield gene that increases the grain number per panicle after mapping QTLs using populations constructed by Koshihikari (cultivars with tall plants and low yields) and Habataki (cultivars with short plants and high yields). With this gene, a new type of super rice with high yield and lodging resistance was developed by MAS (Ashikari et al., 2005). The same MAS procedure was also successfully executed in disease resistance traits in tomato, taste and virus resistance selection in pepper, and bacterial wilt resistance in eggplant (Foolad and Panthee, 2012; Hanson et al., 2016; Khapte et al., 2018; Moodley et al., 2019). In the breeding of special varieties of *Lycium* species, we should strengthen the organic combination of traditional breeding technology and molecular marker-assisted breeding. It cannot only improve the selection efficiency but also greatly shorten the breeding time (fewer years) to implementing early assisted selection with molecular marker-based traditional breeding as the main method (Nan et al., 2017). The present attention of practical breeding is to develop an increasing number of markers using biotechnology tools to enable molecular-assisted selection to accelerate the delivery of improved varieties to agriculturalists (Rubiales et al., 2021).

**FIGURE 1:**
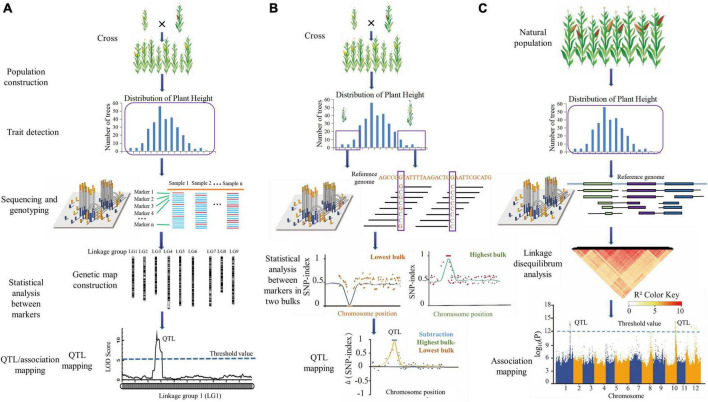
The procedure of MAS combining next-generation sequencing (NGS). **(A)** Linkage mapping; **(B)** bulk segregation analysis (BSA); **(C)** association mapping. Genetic population construction, morphological trait detection, population sequencing and genotyping, statistical analysis between markers, and QTL/association mapping are common procedures in MAS combining NGS.

As shown in Figure 1, the procedure of MAS combined with next-generation sequencing (NGS) breeding mainly includes genetic population construction, morphological trait detection, population sequencing and genotyping, statistical analysis between markers, and QTL/association mapping.

Thus, in this study, we summarize all factors in molecular MAS, including germplasm resources, compatibility between species with karyotypes, candidate traits and their protocols for detection, and the progress of molecular MAS in *Lycium*. Moreover, we also discuss research strategies for different traits and prospective directions of selective breeding in *Lycium* species.

## Germplasm Resources of Goji Berry

By 2001, approximately 80 species of *Lycium* had been found and named from South America to North America, Australia to the Pacific Islands and Eurasia to the South (Fukuda et al., 2001; Shi et al., 2012), whereas in 2018, the number exceeded 97 (Yao et al., 2018a). *Lycium* species are found in a variety of habitats, primarily in temperate and subtemperate climates (Fukuda et al., 2001; Shi et al., 2012). According to statistics, there are 14 species of goji in Eurasia, 26 in South Africa, and 25 in the southern part of North America. Up to 32 species are widely distributed in South America, whereas there are only three in Australia.

**FIGURE 2:**
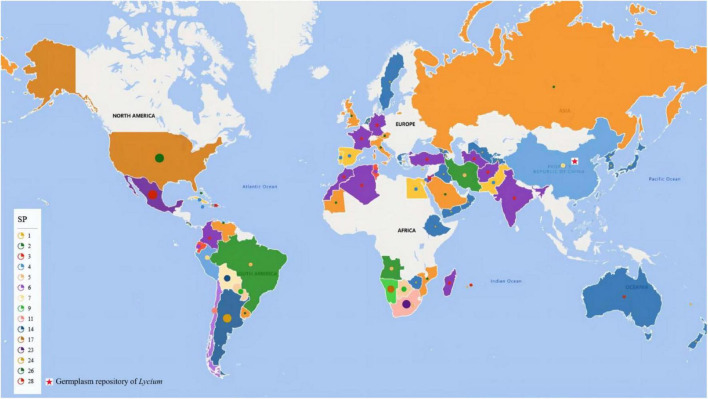
Distribution of *Lycium* species worldwide. SP, species number. Dots with different colors and sizes indicate species numbers in the country marked by them. The red pentacle indicates the location of the germplasm repository of *Lycium*.

**FIGURE 3:**
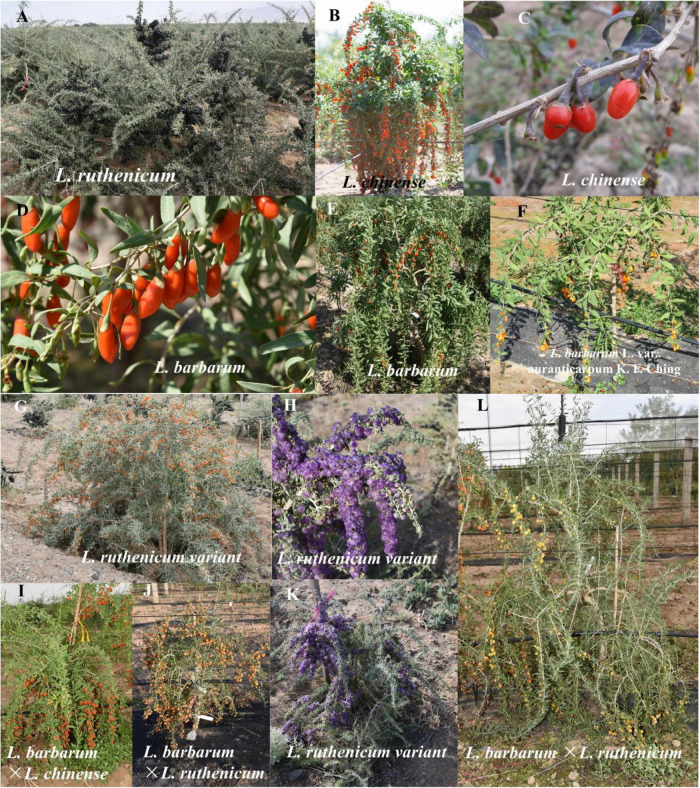
Some collections of Lycium species in the resources. **(A)** *L. ruthenicum*; **(B,C)** *L. chinense*; **(D,E)** *L. barbarum*; **(F)** *L. barbarum* variant; **(G,H,K)** *L. ruthenicum* variant; **(I)** offspring between *L. barbarum* and *L. chinense*; and **(J,L)** offspring cross between *L. barbarum* and *L. ruthenicum*.

To date, as shown in Figure 2, the *Lycium* resources of 86 countries/regions have been reported, among which Mexico and the US have the most abundant *Lycium* species (Editorial Committee of Chinese Flora, Chinese Academy of Sciences, 1978; Yao et al., 2018a). Goji is primarily found in the northern parts of China (Yao et al., 2018a). There are seven species and three varieties of *Lycium* in China including *L. cylindricum*, *L. yunnanense*, *L. dasystemum* along with mutants (*L. dasystemum* var. rubricaulium), *L. truncatum*, *L. ruthenicum* (Figure 3A), *L. barbarum* (Figures 3D,E) along with mutants (*L. barbarum* L. var. *auranticarpum* K. F. Ching) (Figure 3F), and *L. chinense* Mill. (Figures 3B,C) along with mutants [*L. chinense* Mill. var. potaninii (Pojark.) A. M. Lu] (Editorial Committee of Chinese Flora, Chinese Academy of Sciences, 1978; Dong et al., 2008; Yao et al., 2018a), among which five species have medical functions and four can be regarded as food (Editorial Committee of Chinese Flora, Chinese Academy of Sciences, 1978; Li Y. C. et al., 2001; Azadi et al., 2007; Olatunji et al., 2018; Wang et al., 2018; Li et al., 2019a; Ye and Jiang, 2020).

However, among the *Lycium* species, according to the Chinese Pharmacopeia, only *L. barbarum* is recorded as a medicine (Zhang et al., 2001). Several cultivars of *L. barbarum* have been reported, including the Ningqi series (from Ningqi No. 1 to No. 10), Chaiqi series (Chaiqi No. 1, No. 2), Qingqi series (No. 1, No. 2), Jingqi series (from Jingqi No. 1 to No. 6) (Qin and Dai, 2017), and Zhongkelvchuan No. 1 (Yang et al., 2015).

In addition to new species, intercross plants have also been reported, such as *L. ciliaturn* × *L. cestroides* (Bernardello et al., 1995), *L. barbarum* × *L. chinense* (Rehman et al., 2020; Figure 3I), and *L. barbarum* × *L. yunnanense* (Zhao et al., 2021). In the germplasm repository of the Chinese Academy of Sciences (E 106.04968°, 38.4398563°), offspring of *L. barbarum* and *L. ruthenicum* (Figures 3J,L) have also been created. Intercross-demonstration offspring can be used to map QTLs with interspecies crosses.

To provide a reference for germplasm collection in genetic breeding, we summarized the resource diversity in all countries reported (Figure 2 and Supplementary Table 1). These resources with multiple trait variations will provide multiple materials for genetic research on traits related to fruits (color, size, and metabolism), leaves (length, width, and thickness), resistances and so on.

Currently, the *Lycium* germplasm repository at the Goji Engineering Technology Center mainly focuses on Ningxia Province, and it comprises seven species and three varieties distributed naturally in China as well as germplasm imported from the United States, South Korea, Malaysia, and other countries, totaling 60 varieties (lines) and more than 2,000 intermediate materials (1,500 red fruits, 120 yellow fruits, 300 black fruits and 150 other fruits) (NingXia Academy of Agricultural and Forestry Sciences, 2015). The second germplasm resource is in the Chinese Academy of Sciences, which has collected 35 core *Lycium* species resources with different characteristics from all over the world and obtained approximately 10,000 hybrid progenies through intraspecific and interspecific hybridization, providing abundant germplasm resources for breeding and parent plants with rich phenotypes for the construction of NAM (nesting association mapping) populations of *Lycium* species.

## Karyotyping

Cytological examination is a necessary procedure in molecular breeding (Xu, 2010), for validation of both intercrosses and intracrosses in relation to fertility and for avoiding individuals containing translocations and polyploid species containing monosomes or partial chromosomes from being used as mapping parents (Xu, 2010). The cross between tetraploid and diploid *L. barbarum* showed strong cross affinity or backcross affinity; however, the progeny were highly sterile, and the seed satiation rate was very low, making the breeding of triploid seedless plants difficult (Li J. et al., 2001), so it was not conducive to the construction of hybrid populations.

The karyotypes of all of the studied taxa can reveal differences among the species, including size, major visible chromosomal rearrangements, cryptic structural changes, paracentric inversions or reciprocal translocations of segments of similar length (Stiefkens and Bernardello, 2002). A karyotypic analysis showed that *L. barbarum* from Ningxia had 12 pairs of chromosomes (2n = 24) (Chen J. et al., 2013). In the karyotype study, 57 kinds of species, varieties, and hybrid plant were used out of a total of 97 species. Most of the *Lycium* species are diploid with 12 pairs of chromosomes (Laura et al., 2010). Some others are teraploid, octaploid, decaploid, and even hendecaploid (Bernardello et al., 1995; Stiefkens and Bernardello, 2005; Chen J. et al., 2013; Stiefkens et al., 2020; Table 1).

**TABLE 1:** Cytological characteristics of *Lycium* species.

	Taxon	Code	Chromosome number	Karyotype	Tlb	c	r	A1	A2	St	R	References
1	*L. elongatum*	Diploid	24	11 m* + 1 sm	25.01	2.08	1.22	0.16	0.12	1 A	−	Stiefkens and Bernardello, 1996
2	*L. afrum*	Haploid	12	−	−	−	−	−	−	−	−	Minne et al., 1994
3	*L. ameghinoi*	Diploid	24	11 m* + 1 sm	20.2	1.7	1.23	0.17	0.16	2A	1.7	Stiefkens and Bernardello, 2005
4	*L. americanum*	Diploid	24	11 m* + 1 sm	22.74	1.89	1.22	0.16	0.12	1 A	−	Stiefkens and Bernardello, 1996
5	*L. amoenum*	Diploid	24	10 m* + 2 sm	26.76	2.23	1.29	0.2	0.1	1A	1.41	Laura et al., 2010
6	*L. arenicolum*	Triploid	36	−	−	−	−	−	−	−	−	Minne et al., 1994
7	*L. barbarum*	Diploid	24	12 m	49.99	4.17	1.23	−	−	−	1.51	Chen J. et al., 2013
8	*L. bosciifolium*	Diploid	24	11 m* + 1 sm	25.04	2.09	1.25	0.24	0.12	1A	1.49	Laura et al., 2010
9	*L. cestroides*	Diploid	24	11 m* + 1 sm	25.78	2.14	1.22	0.17	0.12	1 A	−	Stiefkens and Bernardello, 1996
		Diploid	24	11 mb + 1 sm	19.19	1.6	1.21	0.15	0.14	−	−	Bernardello et al., 1995
10	*L. chanar*	Diploid	24	11 m* + 1 sm	22.68	1.89	1.17	0.11	0.14	2A	−	Stiefkens and Bernardello, 2002
11	*L. chinense*	Diploid	24	12 m	50	4.17	1.31	−	−	−	1.93	Chen J. et al., 2013
12	*L. chilense* var. chilense	Diploid/Tetraploid	24/48	11 m* + 1 sm	20	1.7	1.14	0.1	0.13	1A	1.52	Stiefkens and Bernardello, 2005
13	*L. chilense* var. confertifolium	Tetraploid	48	22 m + 2 sm	22.61	1.88	−	−	−	−	−	Stiefkens et al., 2020
14	*L. chilense* var. descolei	Diploid/Tetraploid	24/48	11 m* + 1 sm	23.9	2	1.19	0.14	0.12	1A	1.46	Stiefkens and Bernardello, 2005
15	*L. chilense* var. Descolei	Tetraploid	48	22 m + 2 sm	26.32	2.19	−	−	−	−	−	Stiefkens et al., 2020
16	*L. chilense* var. filifolium	Diploid/Tetraploid	24/48	11 m* + 1 sm	30	2.5	1.2	0.15	0.12	1A	1.51	Stiefkens and Bernardello, 2005
*17*	*L. ciliatum*	Diploid	24	11 m* + 1 sm	21.2	1.7	1.21	0.15	0.11	2A	1.4	Stiefkens and Bernardello, 2005
		Tetraploid	48	22 m + 2 sm	22.03	1.84	−	−	−	−	−	Stiefkens and Bernardello, 2002
18	*L. ciliatum*	Diploid	24	11 mb + 1 sm	20.57	1.71	1.24	0.17	0.11	−	−	Bernardello et al., 1995
19	*L. cuneatum*	Diploid	24	11 m* + 1 sm	20.34	1.69	1.18	0.12	0.14	2A	−	Stiefkens and Bernardello, 2002
20	*L. depressum*	Diploid	24	11 m + 1 sm	56.48	4.71	1.44	−	−	2A	−	Sheidai et al., 1999
		Diploid	24	12 m	64.08	5.34	1.3	−	−	1A	−	Sheidai et al., 1999
		Diploid	24	12 m	67.18	5.60	1.83	−	−	1A	−	Sheidai et al., 1999
21	*L. ferocissimum*	Diploid	24	10 m* + 2 sm	22.16	2.02	1.3	0.21	0.08	1A	1.29	Laura et al., 2010
22	*L. fremontii*	Octaploid	96	−	−	−	−	−	−	−	−	Chen J. et al., 2013
23	*L. fuscum*	Diploid	24	11 m* + 1 sm	22.7	1.89	1.22	0.16	0.14	2A	−	Stiefkens and Bernardello, 2002
24	*L. gilliesianum*	Diploid	24	11 m* + 1 sm	20.66	1.72	1.25	0.18	0.17	2A	−	Stiefkens and Bernardello, 2002
25	*L. horridum*	Diploid	24	−	−	−	−	−	−	−	−	Minne et al., 1994
26	*L. humile*	Diploid	24	11 m + 1 sm	21.45	1.79	1.34	0.16	0.11	-	1.71	Stiefkens et al., 2020
27	*L. infaustum*	Diploid	24	11 m* + 1 sm	21.52	1.79	1.25	0.18	0.13	1 A	-	Stiefkens and Bernardello, 1996
28	*L. kopetdaghi*	Diploid	24	12 m	50.91	4.24	1.38	−	−	1A	−	Sheidai et al., 1999
29	*L. makranicum*	Diploid	24	12 m	51.36	4.28	1.32	−	−	1A	−	Sheidai et al., 1999
30	*L. minutifolium*	Diploid	24	11 m* + 1 sm	24.93	2.07	1.21	0.16	0.08	1A	−	Stiefkens and Bernardello, 2002
31	*L. morongii*	Diploid	24	11 m* + 1 sm	23.23	1.94	1.2	0.14	0.12	1A	−	Stiefkens and Bernardello, 2002
32	*L. nodosum*	Diploid	24	11 m* + 1 sm	20.03	1.67	1.27	0.19	0.13	2A	−	Stiefkens and Bernardello, 2002
33	*L. oxycarpum*	Diploid	24	10 m* + 2 sm	23.22	1.93	1.3	0.2	0.08	1A	1.3	Laura et al., 2010
34	*L. pallidum*	Diploid	24	10 m + 2 sm	49.99	4.17	1.22	−	−	−	1.55	Chen J. et al., 2013
35	*L. rachidocladum*	Diploid	24	11 m* + 1 sm	28.01	2.33	1.28	0.20	0.16	1 A	−	Stiefkens and Bernardello, 1996
36	*L. repens*	Hendecaploid	132	−	22.68	1.89	−	−	−	−	−	Stiefkens et al., 2020
37-1	*L. ruthenicum*	Diploid	24	9 m + 3 sm	70.87	5.91	1.5	−	−	2A	−	Sheidai et al., 1999
37-2	*L. ruthenicum*	Diploid	24	9 m + 3 sm	66.84	5.57	1.52	−	−	2A	−	Sheidai et al., 1999
37-3	*L. ruthenicum*	Diploid	24	12 m	50.18	4.18	1.25	−	−	−	1.53	Chen J. et al., 2013
38	*L. shawii*	Diploid	24	11 m + 1 sm	40.92	3.41	1.35	−	−	2A	−	Sheidai et al., 1999
39	*L. stenophyllum*	Diploid	24	11 m* + 1 sm	22.9	1.9	1.17	0.12	0.13	1A	−	Stiefkens and Bernardello, 2002
40	*L. tenue*	Diploid	24	10 m* + 2 sm	21.65	1.8	1.28	0.18	0.1	2A	1.43	Laura et al., 2010
41	*L. tenuispinosum*	Diploid	24	11 m* + 1 sm	−	−	−	−	−	−	−	Stiefkens and Bernardello, 1996
42	*L. tenuispinosum* var. friesii	Diploid	24	11 m* + 1 sm	25.29	2.10	1.21	0.15	0.12	1 A	−	Stiefkens and Bernardello, 1996
43	*L. tenuispinosum* var. tenuispinosum	Diploid	24	11 m* + 1 sm	23.63	1.96	1.21	0.16	0.1	1 A	−	Stiefkens and Bernardello, 1996
44	*L. tenuispinosum* var. calysinum	Diploid	24	11 m* + 1 sm	21.52	1.79	1.21	0.15	0.12	1 A	−	Stiefkens and Bernardello, 1996
45	*L. tetrandrum*	Triploid	36	−	−	−	−	−	−	−	−	Minne et al., 1994
46	*L. villosum*	Diploid	24	−	−	−	−	−	−	−	−	Minne et al., 1994
47	*L. vimineum*	Diploid	24	11 m* + 1 sm	20.01	1.67	1.24	0.17	0.13	2A	-	Stiefkens and Bernardello, 2002
48	*L. australe*	Diploid	24	10 m + 2 sm	31.43	2.62	1.25	0.16	0.12	−	1.61	Stiefkens et al., 2020
49	*L. berlandieri*	Diploid	24	10 m + 2 sm	38.7	3.23	1.36	0.22	0.12	−	1.55	Stiefkens et al., 2020
50	*L. californicum*	Diploid	24	11 m + 1 sm	29.8	2.48	1.19	0.14	0.1	−	1.46	Stiefkens et al., 2020
		Tetraploid	48	22 m + 2 sm	39.65	3.3	−	−	−	−	−	Stiefkens et al., 2020
51	*L. exsertum*	Tetraploid	48	20 m + 4 sm	40.26	3.35	−	−	−	−	−	Stiefkens et al., 2020
52	*L. fremontii*	Decaploid	120	–	29.24	2.44	−	−	−	−	−	Stiefkens et al., 2020
53	*L. intricatum*	Diploid	24	10 m + 2 sm	33.59	2.8	1.24	0.21	0.16	-	1.81	Stiefkens et al., 2020
54	*L. pallidum*	Diploid	24	10 m + 2 sm	29.98	2.5	1.37	0.25	0.11	-	1.49	Stiefkens et al., 2020
55	*L. parishii*	Diploid	24	11 m + 1 sm	30.67	2.55	1.15	0.12	0.08	-	1.36	Stiefkens et al., 2020
56	*Lycium* sp.	Tetraploid	48	20 m + 4 sm	24.52	2.04	−	−	−	−	−	Stiefkens et al., 2020
57	Hybrid (*L. ciliaturn* × *L. cestroides*)	Diploid	24	11 mb + 1 sm	22.23	1.85	1.22	0.16	0.13	−	−	Bernardello et al., 1995

*Tl, mean total haploid chromosome length; C, mean chromosome length; r, mean arm ratio. Mean asymmetry indices: A1, intrachromosomic; A2, interchromosomic; St, Stebbins’ (1971), category of asymmetry; R, ratio between largest and smallest chromosomes in complement. Lengths in μm. m, metacentric chromosome; sm, submetacentric chromosome. An asterisk indicates that the first chromosome pair has a satellite on the short arm.*

## Traits for Quantitative Trait Locus Mapping in *Lycium*

Although *Lycium* species are found all over the world, only *L. barbarum* is frequently planted as fruit trees and medicinal plants, particularly in China (Shi et al., 2012). Thus, the majority of breeding selection research has been conducted in China. Although the Chinese government has recognized nearly 10 top varieties of *Lycium*, only “Ningqi No. 1,” “Ningqi Cai No. 1,” “Mengqi No. 1,” “Ningqi No. 4,” “Ningqi No. 7,” and “Zhongkelvchuan No. 1” have been actively promoted and implemented in production (An et al., 2009; Chen et al., 2018), and the number of new cultivars is still growing. However, the majority of goji fruits on the market are dried fruits in medical use, and fruit juice and fresh fruits are rarely promoted to the market (Huang et al., 2013). As a result, single usage (only dry fruits for medical use) cannot adjust to the demands of industrial product diversification (An et al., 2009). The usage needs to be extended to food, tea, juice, fruit, and chemical extraction uses, such as goji leaf tea, wolfberry tea, wolfberry juice, and wolfberry wine.

Breeders have been grappling with how to standardize the evaluation of germplasm resources and new varieties in recent years, as goji plants have become more widely planted and the demands for germplasm resources have grown (Zhang and Sun, 2011). The State Forestry Administration and the Ministry of Agriculture are currently the main authorities in China for examining and approving new varieties. In anticipation of the acceptance of new *Lycium* variants, the two departments have developed two sets of “Guidelines for the Conduct of Tests for Distinctness, Uniformity, and Stability of *Lycium* species” (DUS) (LY/T2099-2013 and NY/T2528-2013). The number of agricultural qualities covered by the two DUS guidelines is limited, and the breeding requirements cannot be fully met. The DUS testing guidelines for new plant varieties need to be further improved (Zhang and Sun, 2011). Tomato, eggplant, potato, and goji are all members of the Solanaceae family, which is one of the most significant families in agriculture. It possesses a highly conserved genomic sequence and a phenotypic trait set that is extremely diverse. Therefore, comparative genomics across different Solanaceae species can frequently lead to major findings (Bombarely et al., 2010). Currently, international studies on other Solanaceae species, such as tomato and potato, are quite in depth regardless of the variety of features, gene research, or other themes. Plants have been investigated for more than 1,000 trait descriptions worldwide,^1^ of which 387 are for the Solanaceae family (solgenomics.net). The Sol Genetic Network (SGN)^2^ is a professional portal that provides genomic and phenotypic data on Solanaceae and allied species. It compiles information on the genomic and phenotypic characteristics of tomato, potato, pepper, eggplant, tobacco, and other species (Bombarely et al., 2010). In contrast to other crops, research on the resource base of *Lycium* species is still in its early stages, and systematic and in-depth research must be strengthened (An et al., 2009). We combine data from SGN, DUS, “Normative description and data standard of *L. barbarum* germplasm resources” (Shi et al., 2012), and other published papers to provide additional traits and their standardized detection methods and QTL mapping for breeders to facilitate standardization of trait research.

### Whole-Plant Traits

Many genetic traits, such as ground diameter, crown width, plant height, branch hardness, and natural plant type, are dominated by whole-plant attributes, which can be utilized as the primary reference indices for *Lycium* species breeding selection (Yuan et al., 2013). Trunk diameter (TD), for example, is related to the yield, resistance and growth rate of the plant. Unlike the branch angle or tree height, the trunk diameter is unaffected by trimming and can properly depict the plant’s growth state. It is a crucial index for precisely reflecting the plant’s growth status, such as the amount of growth (Miller and Scorza, 2010; Wang et al., 2015b). Wang et al. (2015b) mapped four QTL loci influencing the growth of cherry trunk using a high-density genetic map of *Prunus avium* and discovered that the QTL loci controlling the growth of cherry trunk varied with distinct developmental stages (Wang et al., 2015b). These findings suggested that the QTLs governing trunk growth were stage-specific (Liu et al., 2008). To make production management easier, cultivars with a large branching angle, small tree body, and large trunk diameter have been produced (Wang et al., 2015b). Thus, trunk diameter is extremely important in production.

Whole-plant traits, such as plant height, trunk diameter, and internode length, are regulated by genes that can be detected by QTL mapping in *Lycium* and neighboring species (Frary et al., 2003; Barchi et al., 2009; Gong et al., 2019). Thus, mapping of QTLs and gene mining of plant-related traits are useful.

To standardize the detection of whole-plant-related traits, we summarized thirty kinds of traits and their references (Supplementary Table 2).

### Resistance Traits

Plants of the *Lycium* genus attract a variety of pests due to their lush stems and leaves as well as their sweet fruit juice. According to research and investigation, there are approximately 38 pests in *L. barbarum* in Ningxia, including seven major pests and four diseases, the majority of which are unique to goji plants. If prevention and control are not strengthened in a timely manner, they frequently cause serious goji production problems, even failure, and severely affect yield (Cao and He, 2013). Planting resistant cultivars and spraying fungicides are two possible approaches to controlling pests (Villegas-Fernández et al., 2021). However, continuous use of pesticides can result in loss of biodiversity, environmental contamination, and a slew of ecological issues. Furthermore, widespread contamination of the environment and accumulation in the food chain can pose a risk of teratogenic, genotoxic, oncogenic, and carcinogenic effects on humans (Singh et al., 2017). Thus, stress-resistant cultivar breeding is required.

The first strategy to breeding cultivars with continuous resistance is to select plants with more than resistance genes with the help of robust molecular markers (Rubiales et al., 2021). Research on resistance to biotic stress on neighboring species in Solanaceae, including Capsicum (pepper), Solanum (potato), and Lycopersicon (tomato), showed that these resistances were regulated by resistance genes (R genes). Moreover, homologous genes in the R family (Pto, N, Sw-5, I2, and Prf) in pepper were discovered in syntenous sites in genomes of other solanaceous species and occasionally also discovered to extra sites neighboring phenotypically defined R genes in Solanaceae (Grube et al., 2000). Because resistance is regulated by resistance genes, QTL mapping can be conducted for resistance traits. QTL mapping can be used to further understand the genetic basis of various traits, including resistance to biotic stress (Iordãchescu et al., 2020).

The obtainability of a variety of resistant sources and of a dependable screening technique are crucial to execute a resistance selection project. Study on the molecular and inherited foundation of resistance can improve breeding efficiency (Rispail et al., 2020). To date, there are still no reports about genetic breeding in resistance research on *Lycium*. To facilitate genetic research on resistance in *Lycium* species and genetic resource conservation and evaluation, standardized methods to evaluate resistance to 11 kinds of insects/disease and references were summarized (Supplementary Table 3).

### Phenology Traits

As domestic and worldwide market demand grows, the manufacturing capacity of Ningxia Province cannot keep pace (Yao et al., 2018b). Thus, it is necessary to increase goji berry output in other favorable agricultural areas (Barchi et al., 2009; Gong et al., 2019). The study of phenology aids in the development of *Lycium* species variants that are appropriate for various climate conditions. Higher latitudes and altitudes and shorter growing seasons than the current optimal areas limit the number of cultivars growers choose in outdoor production (Kevany et al., 2008). Qinghai Province, for example, is a plateau-climate producing area (Yao et al., 2018b). The harvest season for goji fruit is from July to November each year. However, in September in Qinghai, the temperature drops below 10°C, drastically shortening the harvest season and resulting in lower yield (Zheng et al., 2018). Growing early maturing cultivars is one way to address climate-related constraints. Growers benefit from these varieties as well because the earliest fruit to market in a season might command a higher price (Kevany et al., 2008). Thus, the ability to have fruits mature early is a very desirable and important feature (Kevany et al., 2008).

Early ripening occurs when the ethylene receptor LeETR4 is suppressed, but fruit size, yield, and flavor-related chemical composition remain mostly intact. Moreover, gibberellins (GAs) were also found to have negative effects on fruit ripening. Restriction of GAs can activate ripening regulator genes such as CNR, RIN, and NOR, mediating the biosynthesis of ethylene. Further study showed that the SlGA2ox1 gene regulated GA catabolism, resulting in reductions in GA levels in fruit tissues and leading to early ripening in tomato (Li et al., 2019b). Biotechnology could provide important tools for the creation of early ripening cultivars as our understanding of the molecular control of fruit ripening grows (Kevany et al., 2008). Thus, to apply MAS in phenology trait QTL mapping and standardize the detection method, we summarized 25 phenology traits in Supplementary Table 4.

### Fruit Traits

The fruit size of *L. barbarum* impacts the price of dry goji fruits and is one of the most important indicators of goji fruit grading (Ma et al., 2017). The higher the wolfberry grade and the larger the fruit size, the higher the price. Therefore, in regard to goji cultivation, breeders are more likely to choose types with large fruit sizes and high yields. Fruit weight, fruit length, and fruit breadth are subdivided into indices for analyzing goji fruit size (An et al., 2007). Meanwhile, crop yield is considered positively correlated with fruit weight, fruit length, and fruit width, showing that superior farming varieties with large fruit length, width, and weight cannot only improve fruit quality but also increase yield (Henareh et al., 2015). Artificial domestication can boost the productivity and quality of numerous crops. When compared with predomestication, the size and weight of tomato fruits grew dramatically with favorable features following long-term artificial domestication (Tanksley, 2004).

Fruit weight in most tomato species is controlled by a major QTL (*fw2.2*) (Alpert et al., 1995). In *Lycium* species, QTLs for fruit traits were first detected by Zhao et al. (2019) with an interspecies genetic map, and 41 stable QTLs were detected (Zhao et al., 2019). QTL mapping for fruit traits was executed by Rehman et al. (2020), with two stable QTLs (Rehman et al., 2020). Genetics are a key factor for fruit traits, and it is feasible to map QTLs with a combination of genetic mapping data and morphological data. Standardized techniques were used to determine the 129 fruit-related traits (Supplementary Table 5).

### Leaf Traits

Leaf traits of *Lycium* include leaf length, leaf width, leaf thickness, petiole length, leaf color et al. (State Forestry Administration of China [SFAC], 2013). The distance between the upper and lower surfaces of the blade is measured as the leaf thickness (LT) (Coneva et al., 2017). Leaf thickness is an important leaf shape parameter for crop plant type improvement and serves as a reference index in the breeding of high-yield crop varieties (Chen D. et al., 2015). Leaf thickness and crop yield have been discovered to have a substantial positive association in rice studies. Plants with thick leaves produce more fruit (Liu C. et al., 2014). As a result, genetic improvement of crop leaf thickness is critical for increasing crop output (Chen D. et al., 2015). Meanwhile, research has discovered that thicker leaves are better for plant growth in arid environments (Coneva et al., 2017).

Photosynthesis is a leaf feature that describes the process by which plants receive light energy and transform it into chemical energy, and it is the foundation of plant organic matter synthesis (Richards, 2000). It is critical to a plant’s ability to synthesize its own chemicals (Long et al., 2006; Zhu et al., 2012). Photosynthetic features include the photosynthetic rate (Pn), stomatal conductance, intercellular carbon dioxide, the transpiration rate, the limiting value of the stoma, water usage efficiency, the chlorophyll content, and a variety of other variables (Qu et al., 2017). Research on other plants shows that polygenes play a major role in photosynthesis (Yang et al., 2007; Kumar et al., 2012; Czyczylo-Mysza et al., 2013). Moreover, Gong et al. (2019) detected 29 QTLs for photosynthetic traits, among which eight are stable QTLs (Gong et al., 2019), indicating that photosynthesis in *Lycium* species is regulated by genes and is applicable to mapping QTLs in *Lycium* species.

Thus, we provide 33 kinds of leaf trait descriptions and their references for standardized detection (Supplementary Table 6).

### Flower Traits

Flowering transition, meristem identity choice, floral organ initiation, and floral organ morphogenesis are the four major steps of tomato flower development (Liu J. et al., 2014). (1) Plants respond to the external environment and their own signals during the flowering transition stage, transitioning from vegetative to reproductive growth. This process is controlled by a set of genes associated to flowering time (Liu J. et al., 2014). In tomato, two flowering control routes have been identified: photoperiod and autonomic. The photoperiod pathway is regulated by uniflora (*UF*) and compound inflorescence (*S*) gene, whereas the autonomous flowering pathway is regulated by single flower truss (*SFT*) gene and jointless (*J*) gene (Dielen et al., 2004; Molinero-Rosales et al., 2004; Liu J. et al., 2014). Flowering time-related genes *S* and *J* are positioned downstream of *UF*, which is a crucial gene in tomato that regulates flowering time (Quinet et al., 2006). Florigen signals are stimulated by SFT protein. The overexpression of *SFT* gene could stimulate flowering in tomatoes (Lifschitz and Eshed, 2006). (2) Plants respond to signals from diverse flowering time regulatory pathways to activate meristem characteristic genes and define meristem attributes during the meristem identity choice stage. After flowering induction, the vegetative meristem becomes the inflorescence meristem (IM) (Liu J. et al., 2014). Lateral organs replace leaves on the inflorescence meristem, which is followed by the flower meristem (FM) (Chandler, 2012). In tomato, the IM motifs SFT, UF, J, and Macrocalyx (MC) have been identified (Liu J. et al., 2014). (3) Meristem signature genes activate floral organ signature genes in various areas during the floral organ initiation stage. Meristem identity genes are critical genes that control the process of IM formation FM and determine the transition from flowering to floral organogenesis. Falsiflora (*FA*) and anantha (AN) were the FM-regulated genes in tomato (Liu J. et al., 2014). (4) Floral organ characteristic genes activate downstream organ morphogenesis genes during the floral organ morphogenesis stage, determining the precise cell types and tissues that make up each organ (Jack, 2004; Liu J. et al., 2014). The ABCE model is used to describe the mechanism of floral organ development (Krizek and Fletcher, 2005). The ABC genes are initially activated by floral meristem identification genes including leafy (*LFY*), apetala 1 (*AP1*), *AP2*, and unusual floral organs (*UFO*) gene, but later expression patterns are adjusted through interactions between the ABC genes. Four activities are present in adjacent whorls of the flower: A, B, C, and E. These four activities are thought to work together with different combinations to determine the identity of the flower’s four organs: sepals, petals, stamens, and carpels (Jack, 2004; Liu J. et al., 2014). In the whole steps, Auxin is also the key factor in determination of the discrimination and quantity of floral organs. The disorder of auxin signaling, transportation or biosynthesis always results in failure of floral discrimination (Cheng and Zhao, 2007).

The relative location of the pistil and stamen has a significant impact on plant fertility. The pistil is more exposed than the stamen in many plant species, and it is the main feature of cross-pollination (Chen and Tanksley, 2004). In hybrid systems of crops, particularly in the application of hybrid rice, the stigma exertion rate plays a vital function (Zhou et al., 2017). The stigma and the top of the stamen tube are almost equal in most wild tomatoes, indicating cross-pollination, whereas in domesticated tomatoes, the stigma and the top of the stamen tube being almost equal indicates self-pollination (Ranc et al., 2008).

The relative location of the pistil and stamen has a significant impact on plant fertility (Chen and Tanksley, 2004). Cross-pollination occurs in the majority of tomato species with exposed stigmas. On the other hand, most tomato species with similar lengths of pistils and stamens or with pistils shorter than the stamen display autocompatibility (Chen and Tanksley, 2004). The pistil of the sterile male varietal Ningqi No. 5 was substantially higher than the stamen among cross-pollinated *L. barbarum* varieties (Chen C. et al., 2017). Thus, the relative location of the pistil and stamen could be an indicator for identifying selfing fertility in *Lycium* plants.

In this section, we describe 27 flower-related traits and their detection references for standardizing the detection method.

### Seed Traits

The seed is the primary site for endogenous hormone synthesis and accumulation, which is critical for fruit development. Previous studies in several fruits have revealed a strong link between fruit seed number and fruit size (Zheng et al., 2015). The incidence of the preharvest decline of fruits is associated with the loss of hormones from the seeds of almost ripe apples (Luckwill, 1948). Seed weight, length, and width have a substantial effect on fruit size development in *L. barbarum* according to previous studies. Seed number, on the other hand, has a greater impact on fruit size development than seed weight (Zheng et al., 2015; Wang J. et al., 2017). For the eight evaluated peach rootstocks, the fresh mass of fruits exhibits a strong positive association with the fresh mass of seeds (Souza et al., 2016). The fresh mass of seeds has a strong association with the germination speed index and mean germination time (Souza et al., 2016). Therefore, it can also serve as a predictor of plant reproductivity. Fruit yield was found to be positively associated with average seed yield and fruit weight per plant in a tomato study. The seed vigor index has the greatest direct effect on seed yield and is regarded as a useful measure for predicting seed lot performance in the field (Sharma, 2012).

According to research, there is a link between fruit development and seed development (Zheng et al., 2012). It reveals that the initial rapid growth phase of the fruit is also the first rapid growth period of the seed, but the seed’s growth ratio is faster than the fruit’s, and the seed’s endosperm grows rapidly during this period. Seeds had slow development characteristics when fruits entered the slow growth stage, and the rate of rise in seed length and width was much lower than the rate of growth of seeds in the initial fast growth stage, during which seeds mostly underwent embryo differentiation. Fruit volume and weight increased fast during the second rapid development stage, although seed length and width increased very slightly. In this stage, only the embryo grew larger (Zheng et al., 2012). Goji seed research shows a similar trend to tomato seed development (Wm Hilhorst et al., 1998). In the early stages, a significant increase in ABA content is invariably followed by an increase in seed weight, however, in the later stages, the ABA content falls and the seed enters the sluggish development stage (Wm Hilhorst et al., 1998).

In seed development stage, a variety of metabolites and jasmonic acid (JA) accumulate in certain organs and tissues. Jasmonate-inducible genes, are expressed in tandem with the accumulation, including developmentally regulated genes and defense genes (Wasternack et al., 2013). In seed formation, coordinated expression of embryo and endosperm is necessary, which is aided by both sporophytic and gametophytic genes from parents (Chaudhury et al., 2001). In the early stages of seed formation, the *SlNAC3* gene (No apical meristem, *NAM*; *ATAF1*/*ATAF2*, and Cup-shaped cotyledon, *CUC* genes in tomato, Solanaceae) in tomato regulates embryo development, while factor for inversion stimulation (*FIS*) regulates endosperm development (Chaudhury et al., 2001; Han et al., 2014).

Although the chromosome ploidy of parents has an effect on seed fertility and the seed satiation rate (Li J. et al., 2001), in the case of good parental compatibility, the seed traits are mainly regulated by genetics. Research on Arabidopsis show that seed weight, size as well as the accumulation of seed oil and protein are regulated by APETALA2 (AP2) (Jofuku et al., 2005). Research on overexpression and mutation of da1-1 allele demonstrated that genes in this family play a key role as controller of organ and seed size in vegetation (Li et al., 2008). Thus, the feasibility of QTLs for seed traits was validated.

Here, eight kinds of traits and their standardized reference are provided (Supplementary Table 8).

### Metabolites

The plant metabolome has a wide range of functions and is frequently viewed as a link between the genome and the phenome (Chen et al., 2016). It can make the link between genes and agronomic features easier. We found additional candidate genes potentially responsible for the variation in traits such as grain color and size using parallel mGWAS (metabolic GWAS) and pGWAS (phenotype GWAS) and provided evidence of a metabotype-phenotype linkage (Chen et al., 2016). Thus, research on mQTLs and mGWAS is crucial. With the advancement of liquid chromatography–mass spectrometry (LC–MS) technology, a large number of metabolites can be detected on a large scale with a targeted or untargeted metabolic profile, relative content, or absolute content (Chen W. et al., 2013; Chen et al., 2016). Khan et al. (2012) discovered 418 metabolites in apple peel and 254 in apple pulp using untargeted LC–MS metabolic profiling, of which 50% in the peel and 44% in the pulp had their mQTLs successfully identified (Khan et al., 2012). Chen et al. (2016) discovered 587 metabolites in cereal using a targeted metabolic profile, of which 331 (56.4%) showed at least one significant relationship, indicating a link between metabotype and phenotype (Chen et al., 2016). Shi created a broad-targeted metabolome approach for detecting 612 compounds in goji fruits in 2019 (Shi et al., 2019). Thus, LC–MS may be used to detect a large number of metabolites for mQTLs (or mGWASs). As Supplementary Table 9 shows, 870 metabolic traits and their reference detection methods have been described (Supplementary Table 9).

In addition, although the metabolic traits include the basic nutritional and medical functions of goji, according to *International Standard* of wolfberry (ISO 23193:2020), *American Herbal Pharmacopoeia*, *British Pharmacopoeia Commission*, *European Pharmacopoeia*, *The standards for wolfberry* in China (GB/T 18672-2014), *Chinese Pharmacopoeia*, *Korean Pharmacopoeia*, and *Vietnam Pharmacopoeia*, polysaccharide, total sugar, protein, zeaxanthin, zeaxanthin dipalmitate, betaine, and ash contents are regarded as quality indicators in wolfberry, and directly determine the level of quality (Korea Food and Drug Administration, 2014; Chinese Pharmacopoeia Commission, 2015; European Directorate for the Quality of Medicines, 2016; Japanese Pharmacopoeia Editorial Committee, 2016; British Pharmacopoeia Commission, 2017; Yao et al., 2018a; The American Herbal Pharmacopoeia, 2019). Thus, they can be key traits in breeding.

### Postharvest Traits

The fruit processing and storage procedures for *L. barbarum* are determined by its postharvest quality. For example, Ningqi No. 1 and Ningqi No. 7 (elite *L. barbarum* cultivars) have excellent yield and disease resistance and are suitable for the production of dried fruits, but they are not suitable for the production of fruit juice due to their bitter and heavy medicinal flavor. Wolfberry with yellow fruits is ideal for making fruit juice because it has no unpleasant tastes, is bright in color, and leaves little residue after juicing (Wang et al., 2016). By examining the changes in the decay index and physiological indices of fresh *L. barbarum* fruit after harvest, Ge et al. (2008) discovered the ideal storage temperature for fresh fruits after harvest (Ge et al., 2008). Thus, mapping QTLs for postharvest traits can help to accelerate cultivar selection for the goji industry for different uses. In tomato, fruit firmness was validated to be regulated by GA2-oxidase, which is encoded by the *FIS1* gene (Li et al., 2020). In *Lycium* species, Rehman et al. (2020) also mapped one QTL (*qFF10–1*) for fruit firmness traits (Rehman et al., 2020), confirming that fruit firmness is a gene-regulated trait and that mapping QTLs by linkage mapping is feasible. Twenty-three kinds of postharvest traits are described in this section for further research (Supplementary Table 10).

## Progress of Marker-Assisted Selection Technology in *Lycium*

### Genetic Marker Development

Molecular markers are based on nucleotide sequence changes, directly reflecting DNA sequence variations. Because molecular markers are not affected by environmental changes, development, differentiation, or cellular defense, compared with traditional phenotypic markers, they are more stable and observable (Agarwal et al., 2008).

The ideal genetic markers have the following characteristics: high genetic polymorphism, high reproducibility (the results can be repeated in different laboratories), codominance (the heterozygosity and homozygosity can pass an experimental test), low price (low cost of marker development and genotype identification), simple operation (automated operation, procedure, and sequencing), neutral selection (no multiple gene effects), markers uniformly distributed throughout the genome, and clear differentiation of alleles (Xu, 2010). Traditional molecular marker techniques include restriction fragment length polymorphism (RFLP), random amplified polymorphic DNA (RAPD), amplified fragment length polymorphism (AFLP), simple sequence repeat (SSR), and NGS-based single nucleotide polymorphism (SNP) (Blenda et al., 2012).

In 2000, molecular markers such as RAPD were first applied in *Lycium* species for the validation of the genetic diversity of *L. barbarum* (Cheng et al., 2000), while RAPD was also validated for its feasibility to distinguish close species of *Lycium* (Zhang et al., 2001). To date, simple sequence repeat (SSR), conserved ortholog set II (COSII), characterized amplified region (SCAR), amplified fragment length polymorphism (AFLP), restriction site−associated DNA sequencing (RAD-seq), internal transcribed spacer (ITS), sequence-related amplified polymorphism (SRAP), intersimple sequence repeat (ISSR), and random amplified, microsatellite polymorphism (RAMP)-PCR markers have been applied in genetic diversity, population structure, morphological variation, phylogenetic inference, traceability, and cultivar/species identification and discrimination (Zhang et al., 2001, 2018; Yin et al., 2005; Sze et al., 2008; Chung et al., 2009; Kwon et al., 2009; Levin et al., 2009; Zhao et al., 2010; Balasubramani et al., 2011; Liu et al., 2012, 2020b,c; Tripathi, 2013; Xin et al., 2013; Chen and Zhong, 2014; Wang et al., 2015a; Chen C. et al., 2017; Chen J. et al., 2017; McCulloch et al., 2020; Jung et al., 2021). SSR markers can be used for the detection of genetic polymorphisms of species, calculation of genetic relationships (genetic distance) between varieties, identification of cultivars, and even construction of genetic maps and QTL mapping with sufficient SSR markers (Chen H. et al., 2015; Essid et al., 2015; Ibrahim et al., 2016; Thammina et al., 2017; Portis et al., 2018b). One hundred fifteen SSR and 12 ILP markers were applied to construct a genetic map with the F1 population (Hu, 2015). However, because of the limited number of markers and huge average genetic distance between two adjacent markers, QTL mapping was not suitable (Hu, 2015). To address this problem, Chen C. et al. (2017) developed 22,537 EST-SSR markers using transcriptomes of different fruit ripening stages.

### Linkage Mapping

Linkage mapping is based on the frequency of recombination between two markers, which can be used to determine their genetic distance on chromosomes. The greater the genetic distance between two markers on a chromosome, the higher their exchange frequency (Xu, 2010). A genetic map depicts the linear order of DNA markers on a chromosome based on their relative positions (Madhusudhana, 2015). The linkage mapping procedure mainly includes constructing mapping population(s), trait data collection, sequencing and genotyping with polymorphic markers, genetic map construction, and QTL mapping (marker-trait associations between genotypic and trait phenotypic data Madhusudhana, 2015; Figure 1A). Linkage mapping can be applied under QTL mapping for multiple traits at the same time (Meuwissen and Goddard, 2004). The linkage mapping (genetic map) population is always constructed with a cross of two parents (Yu et al., 2008). There are several advantages of linkage mapping: (1) The complexity of the population structure is reduced due to parental confirmation, which improves accuracy and reduces false-positives; and (2) the mapping population is constructed by combining the offspring of two parents. Even if the features are regulated by rare genes, the offspring contains a significant proportion of unusual genes. As a result, linkage mapping may be able to locate rare genes (Mackay and Powell, 2007; Yu et al., 2008).

Linkage mapping has been successfully applied in *Lycium* species (Table 2). The intraspecies genetic map consisted of 23,967 markers with a size of 964.03 cM (Gong et al., 2019). According to the genome published by Cao, the size of the genome in *L. barbarum* is 1.67 Gb. Thus, we determined that the average DNA content between two neighboring markers (genome size/marker number) was 69,679 bp. However, the two interspecies genetic maps had higher genetic distances (1702.45 and 1649.03 cM) and lower marker numbers (6,733 and 3,495 cM) (Zhao et al., 2019; Rehman et al., 2020), and the average DNA content between two neighboring markers was 248,032 and 477,825 bp. Obviously, intraspecies genetic maps have higher resolution than intraspecies genetic maps. However, all three maps still have higher resolution than most other species, such as maize (130,000 bp) and grape (676,000 and 606,000 bp in two parent maps) (Chen et al., 2014; Chen J. et al., 2015), which were successfully mapped to QTLs. Thus, mapping QTLs with linkage mapping is applicable to *Lycium* species.

**TABLE 2:** Genetic map construction and QTL mapping in *Lycium* species.

Population type	Parents	Population size	Chr	Length (cM)	No. of markers	Marker interval (cM)	Max interval (cM)	Traits	QTL	References
Intraspecies	Lb × Lb	305	12	964.03	23,967	0.04	1.98	6	32	Gong et al., 2019
Interspecies	Lb × Lc	302	12	1702.45	6,733	0.31	11.7	7	55	Zhao et al., 2019
Interspecies	Lc × Lb	305	12	1649.03	3,495	0.47	16.99	11	117	Rehman et al., 2020

*Lb, L. barbarum; Lc, L. chinense; Chr, chromosome.*

## Other Marker-Assisted Selection Techniques With Potential Value

### Bulk Segregation Analysis

Bulk segregant analysis (BSA) is a QTL mapping technique for locating genomic regions with genetic loci that impact a trait of interest (Michelmore et al., 1991; Magwene et al., 2011). Individuals are assayed for the focal characteristic in a segregating population derived from a genetic cross, and two pools (bulks) of segregants are established by choosing individuals from the phenotypic distribution tails (other sampling designs can also be used as discussed below). Individual genotyping or the production of pooled DNA samples from which allele frequencies are obtained are used to estimate genotype frequencies for the two bulks. In genomic regions with no loci affecting the trait, allele frequencies should be roughly comparable between the two bulks. Allele frequencies should differ between bulks in regions of the genome that include causative loci. High marker density and accurate allele frequency estimation inside bulks are the most effective features of BSA (Ehrenreich et al., 2009; Magwene et al., 2011; Lu et al., 2021).

Although utilization of BSA to map QTLs in *Lycium* species has not yet been reported, BSA, with its cost-effectiveness and efficiency, has been widely used to map QTLs in woody, herbaceous species, including apple and watermelon (Dong et al., 2018; Liu et al., 2021), as well as neighboring species in Solanaceae, such as fruit color in pepper, tomato, and eggplant (Lee et al., 2020; Lu et al., 2021; Tang et al., 2021). Thus, it has the potential for application in *Lycium* species.

The BSA procedure is performed by following key points: establishing a segregating population, collecting trait data and performing bulk selection. Individuals with high and low values for the trait of interest are phenotyped and selected. These individuals’ DNAs are pooled into high and low bulks, which are then sequenced and SNP-called, obviating the requirements to design markers ahead of time and to statistically analyze linkage distances among markers of the two bulks. SNPs discovered in reads derived from regions not connected to the trait of interest should be approximately 50% of the reads in bulks selected from populations (Figure 1B). Depending on the bulk, SNPs in reads aligning to genomic regions directly associated with the trait should be over- or underrepresented. Under QTL mapping, QTL identification can be accomplished by comparing relative allele depths or SNP indices (defined as the number of reads containing an SNP divided by the total sequencing depth at that SNP) between the bulks (Mansfeld and Grumet, 2018). BSA is a cost-effective and efficient approach to fine-mapping genes in unsequenced genomes by targeting SNPs to specific genomic intervals (Trick et al., 2012).

### Association Mapping

Genome-wide association study (GWAS) is an analytical method based on linkage disequilibrium of genes or loci among biological populations, combining genotype and phenotypic data from mapping populations and analyzing the relationship between detection markers or loci and traits using statistical methods (Yu and Buckler, 2006). The procedure of association mapping comprises population selection, morphological data collection, NGS and genotyping, linkage disequilibrium analysis, and association mapping between markers and traits (Zhu et al., 2008; Figure 1C).

GWAS mapping technique is regarded as a substitute for linkage mapping for research on association between genotype and phenotype. Based on recombination to reorganize the genome, both techniques are for evaluating associations between genotype and phenotype. Linkage disequilibrium (LD), the term to evaluate the non-random connotation between markers, was the basis of GWAS (Barchi et al., 2019). GWAS offers three advantages over linkage analysis: (1) High precision and resolution. Linkage disequilibrium (LD) declines quickly after multiple hybridization, recombination, and mutation information from associated populations, and localization precision is considerably increased with high resolution. (2) Without the constraints of the parental population, multiple gene types can be discovered on the same locus. (3) Time efficiency is achieved by using natural populations as a source of material, which reduces the time required to produce genetic populations (Yu and Buckler, 2006; Madhusudhana, 2015; Hu et al., 2018). To date, there is still no report about the utilization of association mapping in *Lycium* species.

However, utilization in neighboring species in Solanaceae, such as QTLs for leaf-, plant-, and fruit-related traits in eggplant (Portis et al., 2015), virus resistance in pepper (Tamisier et al., 2020), and multiple root- and fruit quality-related traits in tomato (Tamisier et al., 2020), demonstrates potential utilization in the breeding of *Lycium* species.

## Mapping Population and Strategies of High-Density Genetic Map Construction in *Lycium*

### Classification of Mapping Populations

**FIGURE 4:**
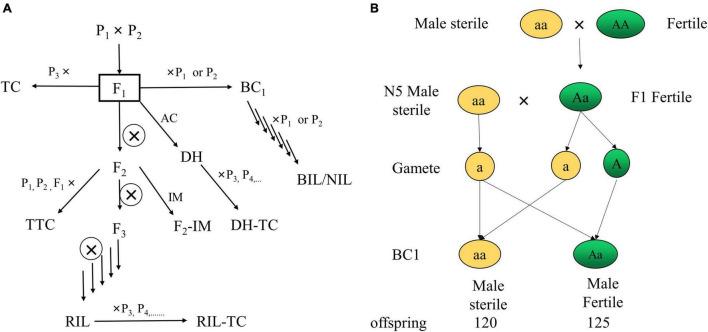
Classification of mapping populations **(A)** (Xu, 2010) and strategy of population construction through sterile male QTL mapping **(B)**. P_*n*_, parent; F1, filial generation 1; AC, mapping population can be classified into anther culture; BC, backcross population; BIL, backcross inbred line; DH, double haploid; IM, intermating; NIL, near-isogenic line; RIL, recombinant inbred line; TC, testcross; TTC, triple testcross; N5, a sterile male cultivar of *L. barbarum*.

The mapping population can be classified into anther culture (AC), backcross population (BC), backcross inbred line (BIL), double haploid (DH), intermating (IM), near-isogenic line (NIL), recombinant inbred line (RIL), testcross (TC), and triple testcross (TTC) (Xu, 2010; Figure 4A).

### Mapping With F_1_ Population

Self-incompatibility is common among woody plants. After a lengthy period of evolution, *Lycium species*, like other woody plants, are genetically polymorphic and extremely heterozygous (Zhao et al., 2010). Goji are woody plants with a long development cycle, high genomic heterozygosity, and difficulty acquiring homozygous genes, making the genetic map of woody plants more difficult to generate than that of herbaceous species (Song et al., 2012; Guo et al., 2015; Zhang et al., 2015). However, the high heterozygosity of woody plants satisfies the requirements for genetic map construction and QTL mapping using the F_1_ population. Thus, all genetic maps of woody plants and some highly heterozygous perennial herbs, such as *Prunus avium*, *Juglans regia*, *Camellia sinensis*, *Paeonia* Sect., *Prunus mume*, and *Cynara cardunculus*, are currently based on F_1_ populations (Cai et al., 2015; Ma et al., 2015; Wang et al., 2015b; Zhang et al., 2015; Zhu et al., 2015; Portis et al., 2018a). To date, three genetic maps of *Lycium* species have been constructed using the F_1_ population. The first high-density genetic map was constructed with an intraspecies cross population of *L. barbarum* (Gong et al., 2019), while the other two were *L. barbarum* and *L. chinense* interspecies cross populations (Zhao et al., 2019; Rehman et al., 2020).

### Mapping With Other Population

Although the F_1_ population is suitable for genetic map development, as shown in Figure 4B, several features, particularly qualitative features governed by recessive alleles, show no distribution in F_1_ offspring, according to our research. Thus, selfing, sib mating and back-crossing to construct F_2_ or BC1 populations are necessary. For example, Ningqi No. 5 (N5) is a *L. barbarum* (Chen C. et al., 2017) sterile male cultivar, and we crossed N5 with Zhongkeluchuan No. 1 to examine sterile male features. The F_1_ offspring, on the other hand, are entirely reproductive males. As a result, we backcrossed with N5 and obtained 120 sterile males and 125 fertile plants. Thus, we obtained an F_2_ population with male sterility segregation (sterile males: fertile plants = 1:1) that can be used for QTL mapping, and we discovered that male sterility is regulated by a single recessive gene. The segregations derived from diploid parents can be classified into eight segregations (ab × cd, ef × eg, hk × hk, lm × ll, nn × np, aa × bb, ab × cc and cc × ab), and only homozygous segregation pattern (aa × bb) is suitable for constructing the genetic map in BC1 and F2 populations (Fernando, 1982; Yu et al., 2013; Zhang et al., 2013). According to Zhao et al. (2019) and Rehman et al. (2020), the number of genotypes (aa × bb) in the parent (*L. barbarum* × *L. chinense*) is 17,513 (17.02%) and 24,329 (59.9%), respectively, indicating that the F_2_ and BC1 populations can be used to generate genetic maps and map QTLs in *Lycium* species. Furthermore, using the newly published (Cao et al., 2021) *L. barbarum* genome by Cao et al. (2021) as a reference genome, we can map QTLs using a more cost-effective method, such as BSA.

### Multiparental Population

When investigating natural populations, genome-wide association analysis offers numerous advantages, but it also has many drawbacks: (1) the population structure has an impact on the accuracy of GWAS analysis. Locus identification is prone to false-positives when the population structure is complicated. (2) Rare genes in populations are challenging to find using GWAS. Due to their low proportion and wide distribution in the population (linkage mapping), rare genes are easily filtered out of analyses (Lander and Schork, 1994). Linkage map analysis, on the other hand, has the following drawbacks: (1) due to the low frequency of marker recombination, the coverage of markers in broad chromosome regions is low (Yu et al., 2008; Hu et al., 2018); (2) a mapped population cannot be used for numerous traits because the population is only derived from the cross of two parents and the number of variant characteristics is limited compared with association mapping (Yu et al., 2008); and (3) it takes a long time to build the population because it is built by crossing (Madhusudhana, 2015).

However, in terms of prior knowledge, cross-validation, and statistical power, linkage analysis and association mapping are complementary to one another (Wilson et al., 2004). The genetic population of association mapping, on the other hand, is a natural population, whereas linkage mapping is invariably a biparental population. Yu et al. (2008) suggested the nested association mapping (NAM) technique to integrate the benefits of both linkage and association mapping strategies (Madhusudhana, 2015). The Buckler lab at Cornell University introduced nested association mapping (NAM) for the first time in 2008. Large populations with many small populations can be created by hybridizing multiple parents with a common parent (Buckler et al., 2009). The inbred line B73 was chosen as the common parent in the maize NAM population construction, and it was crossed with 25 other inbred lines with a wide genetic variety and then self-crossed to the F_5_ generation using the single-grain transfer method. Finally, 25 RIL populations were acquired. There were 200 recombinant inbred lines (RILs) in each population (Madhusudhana, 2015). The use of the NAM population for QTL mapping has two obvious benefits: (1) because the NAM population has distinct parents, both association and linkage analyses can be performed at the same time, and mapping approaches are more diversified and versatile; (2) the population structure can be simplified, and the false-positives generated by the complicated population structure can be minimized because there is a common parent and a clear population structure (Madhusudhana, 2015). Minor changes are required to apply the NAM population to *Lycium*. Unlike herbaceous plants that have a brief lifespan, goji species are shrubs that live for decades (Cao and He, 2013) and can be bred by cuttage (Huang et al., 2015), which is convenient for germplasm resource conservation. As a result, there is no need to create a recombination inbred line. Furthermore, because goji species, like other shrub plants, are heterozygous (Zhao et al., 2010), they are excellent for mapping with F_1_ populations. Conclusively, NAM populations can be created using F_1_ populations crossed by a common parent with distinct cultivars/lines. After modest modifications, other multiparental populations, such as multiparental advanced generation inter-crosses (MAGIC) (Pascual et al., 2015), random-open-parent association mapping (ROAM) (Glowinski and Flint-Garcia, 2018), and complete-diallel plus unbalanced breeding-derived inter-crosses (CUBIC) (Liu et al., 2020a), can be used in *Lycium* breeding.

## Perspectives of *Lycium* Species Breeding

### Mechanized Harvest

In China, the level of mechanization of Chinese medicinal materials is rather low; production is primarily through manpower, and the instruments are simple (Junfa et al., 2009), particularly for goji berries. Currently, the harvesting of goji is mainly reliant on manpower, resulting in low picking efficiency and high picking costs (Zheng et al., 2018). Furthermore, with the rapid migration of rural workers to urban areas, agricultural labor costs are rising. With a comprehensive understanding of farmers’ labor (Junfa et al., 2009) costs, it is critical to build harvesting machinery systems (Zheng et al., 2018). A one-time picking technique, which requires crop growth periods and harvest times to be coordinated, can be utilized to cut labor costs and use mechanization (Zheng et al., 2018). However, due to the complicated growth features of goji fruit, mechanical harvesting is challenging due to the presence of ripe fruits, immature fruits, and blossoms on the same branch at the same time (Zheng et al., 2018). As a result, cultivating new varieties that are compact, tolerant to dense planting, and suitable for mechanized operation and have synchronized fruit ripening and compact plant architecture, has become the primary goal of agricultural genetic improvement (Guo et al., 2019). In coffee fruits, synchronized flowering and fruiting and a reduction in fruit removal force were shown to be ideal for mechanical harvesting (Tongumpai, 1993). Thus, we need to determine which characteristic makes fruit ripening more coordinated. The fruit ripening period of plants with a flower terminal is comparatively concentrated, according to tomato research, which is helpful to mechanical harvesting (Li et al., 2018; Guo et al., 2019). Flower production and limited growth at the apex of the branch can promote uniform fruit ripening, providing theoretical support and practical direction for plant type breeding suitable for mechanical harvesting operations (Guo et al., 2019). For many years, crop physiology, culture, and breeding have focused on improving flower terminal characteristics. Centroradialis (CEN) gene expression increases in the inflorescence, and the apex stops growing and blossoms, indicating apical capping (Bradley et al., 1996). The flower terminal of the tomato was found to be controlled by a gene, and gene editing might be used to domesticate it (Pnueli et al., 1998; Li et al., 2018).

**FIGURE 5:**
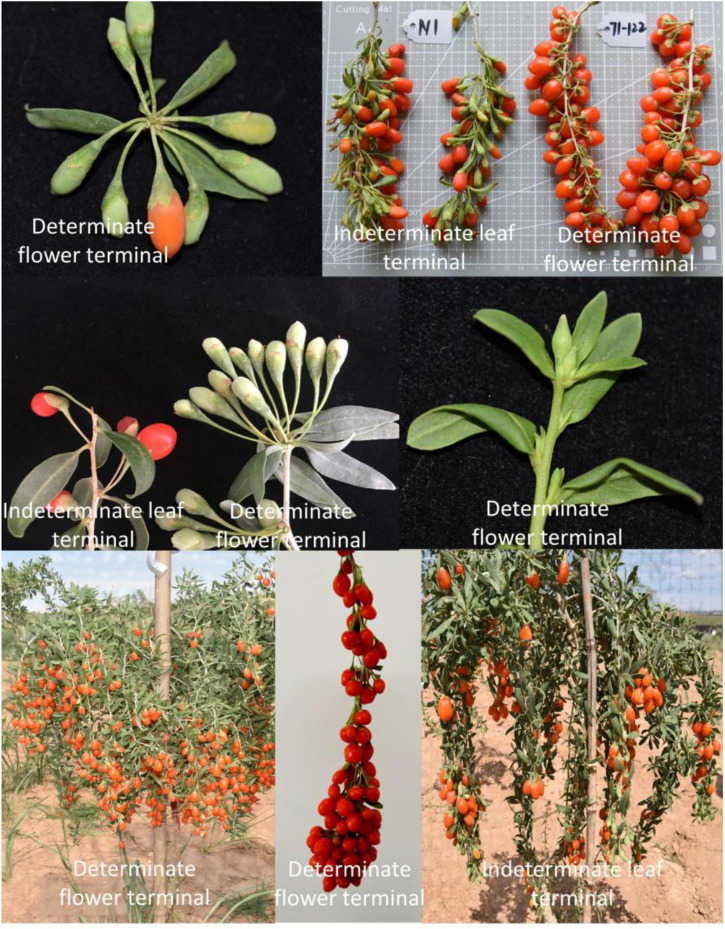
Comparison of cultivars with indeterminate leaf terminal and determinate flower terminal.

The M1 strain developed in the early stages of our research group was used as the female parent, and Zhongkeluchuan1 was used as the male parent. A mutant plant with the flower terminal attribute was established in the established genetic population (population size, 425 plants). The branch tip stops growing when the new branches reach 40 cm, and the apical meristem develops into a cluster of flowers (Figure 5). The ripening rate of the fruits on the same branch is very consistent. The discovery of this germplasm will aid in the future identification of candidate genes for controlling flower terminal characteristics in *L. barbarum*. The directional breeding of congruent varieties of *L. barbarum*, as well as the functional verification of genes and the establishment of molecular markers, provides a solid theoretical and practical foundation.

Thus, to fulfill this aim, we could select approximately 300 offspring and their parents as genetic populations to construct a high-density genetic map with linkage mapping (SLAF-seq or ddRAD-seq) technology according to previous studies (Figure 1A, steps 1, 3, and 4) (Gong et al., 2019; Zhao et al., 2019; Rehman et al., 2020). For flower terminal trait detection, traits No. 8 (flower terminal) and No. 9 (flower terminal rate) in Supplementary Table 7 can be reference methods (Figure 1A, step 2). Finally, as Figure 1A and Step 5 show, QTLs for flower terminals could be mapped in a genetic map with the combination of sequencing and phenotypic data. In addition, BSA-seq could also be used as an alternative, as shown in Figure 1B.

The detected QTLs could be developed as markers for determinate terminal trait selection in *Lycium* species, which will accelerate the selection of flower terminal cultivars. Moreover, the publication of the genome of *L. barbarum* (Cao et al., 2021) enables the mining of candidate genes for further study, such as candidate gene validation, rapid breeding of cultivars with determinate flower terminal using transgenic technology, and studies on the mechanism of terminals flower development.

### Cultivars for Fruits

The goji products on the market right now are mostly dried fruits; however, the drying process causes losses of proteins, carotenoids, fatty acids, and other bioactive elements (Reeve et al., 2010; Hu et al., 2011). Fresh fruits, with their complete nutritional value and delicious flavor, can meet consumer demands for functional fruits. Therefore, fresh wolfberry, as a new functional fruit, has large market potential (Huang et al., 2013). The goji industry should be built on the basis of medicinal value and expanded on the basis of editable value. Thus, we should move beyond the research category of therapeutic impact and broaden the area of fresh food research to broaden the application and industrial scope (Qin and Dai, 2017). Fresh fruits, as opposed to dried fruits, must have a stunning appearance, a lengthy fruit, thick flesh, hard skin, a sweet and good taste, and other favorable traits. However, most modern cultivars have plump pulp, thin skin, and a high moisture content. They are easily damaged while being picked and during transportation, resulting in cracked peels and eventually moldy fruits (Li et al., 2010). Therefore, fruit color, size (especially length), pulp thickness, peel stiffness, and sensory taste should be prioritized in *Lycium* fruit breeding.

**FIGURE 6:**
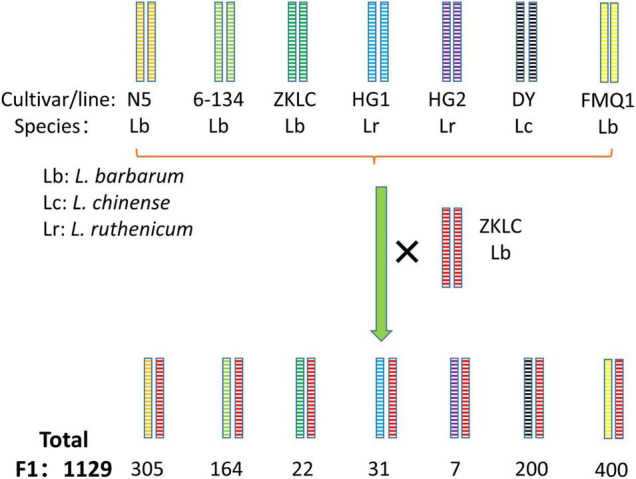
Construction of NAM population in *Lycium*. cultivar/line of *L. barbarum*: N5, 6-134, ZKLC, FMQ1; cultivar/line of *L. chinense*: DY; cultivar/line of *L. ruthenicum*: HG1, HG2; × : cross.

Obviously, there are many phenotypic requirements in fruit cultivars. To facilitate the breeding of fruit cultivars, we need a population with segregations of all traits required. However, it is not easy to construct a biparental population with so many distinguishing variations. Thus, the NAM population, constructed by crossing multiple parents and one common parent (Figure 6), with sharp segregations of multiple kinds of traits, could be a suitable option. NAM populations plus both association mapping and linkage mapping technologies could map QTLs for multiple fruit use related traits, with both high resolution and precision. Moreover, besides the contribution to QTL to facilitate breeding of fruit use cultivar (same to QTL for flower terminal trait), NAM population could cause more plant with more significant phenotypic variations, such as fruit color, fruit shape, leaf shape, plant shape, and et al. (Figures 3G–J), which facilitate the preservation of germplasm resources for ornamental horticulture and other potential uses.

### Improving Light Use Efficiency

Global food crop production has doubled in the last two decades and continues to rise (Evans, 1996). According to Richard’s research, the increases in grain yield in recent decades have two characteristics: (1) photosynthesis theory can be used to boost agricultural yields; (2) photosynthesis-related genetic theories have not been used to boost crop yields (Richards, 2000). Photosynthesis is genetically controlled and plays a crucial role in plant development, biomass buildup, and crop yield (Qu et al., 2017). Therefore, photosynthesis has not been completely utilized in modern agricultural production despite having a great development potential for increasing crop yields (Long et al., 2015). Improving the photosynthetic rate is thought to be one of the most essential ways to boost crop yield (Makino, 2011). There are a variety of techniques to boost the net photosynthetic rate, including increasing light gathering capacity, light energy conversion efficiency, CO_2_ fixation ability, and CO_2_ conversion efficiency in leaves (Ort et al., 2015). However, there have been few findings on genes that control the photosynthetic biosynthesis pathway (Osipova et al., 2016). The genes that affect the photosynthetic rate and water use efficiency are being studied to improve these qualities (Zheng et al., 2011).

There are sharp differences in photosynthesis between cultivars and species in Lycium, such as *L. cylindricum* (net photosynthetic rate, Pn, 9.40 μmol CO_2_ s^–1^ m^–2^), *L. yunnanense* (14.18 μmol CO_2_ s^–1^ m^–2^), *L. dasystemum* (19.46 μmol CO_2_ s^–1^ m^–2^), *L. ruthenicum* (7.29 μmol CO_2_ s^–1^ m^–2^), *L. barbarum* L. var. *auranticarpum* K. F. Ching (8.69 μmol CO_2_ s^–1^ m^–2^), *L. barbarum* “Ningqi-5” (9.20 μmol CO_2_ s^–1^ m^–2^) and *L. barbarum* L. “Zhongkelüchuan-1” (18.19 μmol CO_2_ s^–1^ m^–2^) (Cao and Wu, 2015; Gong et al., 2019), providing multiple resources for research on photosynthetic traits. Moreover, Gong et al. (2019) detected 29 QTLs for photosynthetic traits, including Pn, water use efficiency (WUE), limiting value of the stoma (Ls), transpiration rate (Trmmol), intracellular carbon dioxide (Ci), and stomatal conductance (Cond) (Gong et al., 2019), which will greatly shorten the breeding procedure. Existing photosynthetic QTLs could be developed as markers for photosynthetic trait selection in *Lycium* species, which will accelerate the selection of flower terminal cultivars. The other steps, including candidate gene mining, validation, rapid breeding of cultivars with highly efficient photosynthesis and study of the mechanism of photosynthesis, are the same as the procedure for research on flower terminal traits mentioned above.

### Role of Marker-Assisted Selection in the Cultivation and Conservation of Germplasm Resources and Research on Multiple Traits

MAS with NGS consists of germplasm resource collection, population construction, trait detection, sequencing and genotyping, genetic statistical analysis, and QTL mapping (Figure 1). Thus, research on multiple traits with MAS could facilitate not only the breeding of elite cultivars but also cultivation and conservation of new germplasm resources.

Worldwide, there are 97 species with different variations and functions in *Lycium* (Yao et al., 2018a), and they provide a variety of germplasm resources for cultivation and breeding in goji plants. Here, we summarized the distribution of all species in *Lycium*, species richness in all countries worldwide, location of existing germplasm repositorys, and functions of different species according to existing published studies (Figure 2 and Supplementary Table 1), simplifying and facilitating the collection and conservation of germplasm resources.

Most of the population in MAS was constructed by crossing. Different ploidies of chromosomes in parents may cause highly sterile progeny and a low rate of seed satiation, resulting in difficult breeding of seedless plants (Li J. et al., 2001). Validation of karyotype analysis in parents is necessary (Xu, 2010). To select intra- and interspecies parents for crossing and evaluate their fertilities, Table 2 could be referenced. Table 2 summarizes the karyotype analysis of 50 species, eight varieties, and one hybrid plant, among which 42 species had the diploid type. Crosses can occasionally create plants with new variations, such as fruit color (Figure 3) and flower terminals (Figure 5), which will facilitate the cultivation of new germplasms.

Compared with other crops, studies on the resource base of *Lycium* species, including systematic and in-depth research, are still lagging behind (An et al., 2009). Conversely, studies on other Solanaceae species, such as tomato and potato, are quite in depth, regardless of gene research, the variety of features, or other themes. Thus, the conclusion in our research on 1,144 traits (274 agronomic and 870 metabolic) and their standardized detection based on tomato, *Lycium*, and other Solanaceae species in the prospective trait supplying section (Supplementary Tables 2–10) will facilitate the morphological evaluation, conservation and breeding of *Lycium* germplasm.

QTLs derived from sequencing data and phenotypic data have two kinds of usages. One is for marker development, which could accelerate the selection for elite cultivars with specific trait selection. The present emphasis on practical breeding is incorporating biotechnological methods for the development of a variety of robust markers to execute MAS, in order to speed up the breeding of elite cultivars and delivery of them to the producer (Rubiales et al., 2021), such as the breeding of elite cultivars in grain, tomato, pepper, and eggplant (Foolad and Panthee, 2012; Hanson et al., 2016; Khapte et al., 2018; Moodley et al., 2019). The other is for genetic and mechanistic research of the traits, for example, rice grain. A gene (Gn1a) encoding cytokinin oxidase/dehydrogenase was detected after mapping QTLs for grain yield. The enzyme was found to restrict the synthesis of the phytohormone cytokinin. After a series of validations, the mechanism was successfully demonstrated as gene-cytokinin oxidase/dehydrogenase-phytohormone cytokinin-grain yield (Ashikari et al., 2005).

Finally, after reviewing many studies, we came to a conclusion on the future breeding research direction of *Lycium* species. This review will help to guide future *Lycium* breeding studies.

## Author Contributions

HG: conceptualization, methodology, software, writing—review and editing, and visualization. FR, SZ, ZL, and TY: validation and formal analysis. BA: investigation. YM: resources. DW: data curation. JH: writing—original draft preparation. YW: supervision, project administration, and funding acquisition. All authors contributed to the article and approved the submitted version.

## Conflict of Interest

The authors declare that the research was conducted in the absence of any commercial or financial relationships that could be construed as a potential conflict of interest.

## Publisher’s Note

All claims expressed in this article are solely those of the authors and do not necessarily represent those of their affiliated organizations, or those of the publisher, the editors and the reviewers. Any product that may be evaluated in this article, or claim that may be made by its manufacturer, is not guaranteed or endorsed by the publisher.

## Footnotes

1
www.cropontology.org

2
http://solgenomics.net
