# Supplementary material for: Germplasm Resources and Strategy for Genetic Breeding of Lycium Species: A Review
Source: Front Plant Sci. 2022 Feb 11;13:802936. doi: 10.3389/fpls.2022.802936 (PMC8874141; doi:10.3389/fpls.2022.802936)
Supplement: Supplementary file 1 [file Table_1.docx]

Table S1 the resource diversity in every country reported worldwide

| Region | Species | Food Use | Medicine Use | Reference |
| --- | --- | --- | --- | --- |
| Afghanistan | L. depressum Stocks |  | medicine | (Yao et al., 2018) |
| Afghanistan | L. ruthenicum Murray | food | medicine | (Editorial Committee of Chinese Flora, Chinese Academy of Sciences, 1978) |
| Africa | L. barbarum L. | food | medicine | (Sun et al., 2011) |
| Algeria | L. europaeum L. | food | medicine | (Bendjedou et al., 2021) |
| Algeria | L. intricatum Boiss |  | medicine | (Yao et al., 2018) |
| Algeria | L. schweinfurthii Dammer |  | medicine | (Mamdouh et al., 2021) |
| Angola | L. bosciifolium Schinz | food |  | (Ndithia and Perrin, 2006) |
| Angola | L. decumbens Welw. ex Hiern |  |  | (Levin et al., 2006) |
| Angola | L. horridum Thunb. |  |  | (Yao et al., 2018) |
| Angola | L. oxycarpum Dunal |  | medicine | (Arnold et al., 2002) |
| Angola | L. tetrandrum Thunb. | food |  | (Watt and Breyer-Brandwijk, 1986) |
| Argentina | L. ameghinoi Speg. |  |  | (Yao et al., 2018) |
| Argentina | L. americanum Jacq. | food |  | (ARENAS and SCARPA, 2007) |
| Argentina | L. athium Bernardello |  |  | (Yao et al., 2018) |
| Argentina | L. cestroides Schltdl. |  | medicine | (Rondina et al., 2008) |
| Argentina | L. chanar Phil. |  |  | (Villavicencio et al., 2005) |
| Argentina | L. chilense Bertero |  | medicine | (Levin and Miller, 2005a) |
| Argentina | L. ciliatum Schltdl. |  | medicine | (Trillo et al., 2010) |
| Argentina | L. cuneatum Dammer |  |  | (Yao et al., 2018) |
| Argentina | L. cyathiformum C.L. Hitchc. |  |  | (Hitchcock, 1932) |
| Argentina | L. elongatum Miers |  | medicine | (Toledo et al., 2014) |
| Argentina | L. fuscum Miers |  |  | (Dalmasso and Márquez, 2018) |
| Argentina | L. gilliesianum Miers |  |  | (Yao et al., 2018) |
| Argentina | L. glomeratum Sendtn. |  |  | (Yao et al., 2018) |
| Argentina | L. humile Phil. |  |  | (Yao et al., 2018) |
| Argentina | L. infaustum Miers |  |  | (Yao et al., 2018) |
| Argentina | L. minutifolium Remy |  |  | (Yao et al., 2018) |
| Argentina | L. morongii Britton |  |  | (Yao et al., 2018) |
| Argentina | L. nodosum Miers |  |  | (Yao et al., 2018) |
| Argentina | L. repens Speg |  |  | (Yao et al., 2018) |
| Argentina | L. schreiteri F.A.Barkley |  |  | (Yao et al., 2018) |
| Argentina | L. stenophyllum J. Rémy |  |  | (Yao et al., 2018) |
| Argentina | L. tenuispinosum S.B. Jones & W.Z. Faust |  |  | (Yao et al., 2018) |
| Argentina | L. tenuispinosum var. friesii (Dammer)C.H. Hitchc. |  |  | (Yao et al., 2018) |
| Argentina | L. vimineum Miers |  |  | (Levin and Miller, 2005b) |
| Armenia | L. anatolicum A.Baytop & R.R.Mill |  |  | (Yao et al., 2018) |
| Asia | L. barbarum L. | food | medicine | (Gong et al., 2016) |
| Asia, | L. chinense Mill. | food | medicine | (PARK et al., 2012) |
| Australia | L. australe F.Muell | food |  | (Jeanes, 1999) |
| Australia | L. cestroides Schltdl. |  | medicine | (Rondina et al., 2008) |
| Australia | L. ferocissimum Miers | food | medicine | (McCulloch et al., 2020) |
| Austria | L. barbarum L. | food | medicine | (Kocyigit and Sanlier, 2017) |
| Austria | L. chinense Mill. | food | medicine | (Yao et al., 2018) |
| Bahamas | L. americanum Jacq. | food |  | (ARENAS and SCARPA, 2007) |
| Bahamas | L. tweedianum Griseb. | food |  | (Roth and Lindorf, 2013) |
| Bolivia | L. americanum Jacq. | food |  | (ARENAS and SCARPA, 2007) |
| Bolivia | L. cestroides Schltdl. |  | medicine | (Rondina et al., 2008) |
| Bolivia | L. chanar Phil. |  |  | (NCN, 2004) |
| Bolivia | L. chilense Bertero |  | medicine | (Yao et al., 2018) |
| Bolivia | L. ciliatum Schltdl. |  | medicine | (Trillo et al., 2010) |
| Bolivia | L. cuneatum Dammer |  |  | (Yao et al., 2018) |
| Bolivia | L. cyathiformum C.L. Hitchc. |  |  | (Yao et al., 2018) |
| Bolivia | L. distichum Meyen |  |  | (Yao et al., 2018) |
| Bolivia | L. divaricatum Rusby |  |  | (Yao et al., 2018) |
| Bolivia | L. glomeratum Sendtn. |  |  | (Yao et al., 2018) |
| Bolivia | L. infaustum Miers |  |  | (Yao et al., 2018) |
| Bolivia | L. morongii Britton |  |  | (Yao et al., 2018) |
| Bolivia | L. nodosum Miers |  |  | (Yao et al., 2018) |
| Bolivia | L. tweedianum Griseb. | food |  | (Roth and Lindorf, 2013) |
| Botswana | L. arenicolum Miers |  |  | (Yao et al., 2018) |
| Botswana | L. bosciifolium Schinz | food |  | (Ndithia and Perrin, 2006) |
| Botswana | L. cinereum Thunb. | food | medicine | (Yao et al., 2018) |
| Botswana | L. hirsutum Dunal |  |  | (Yao et al., 2018) |
| Botswana | L. horridum Thunb. |  |  | (Yao et al., 2018) |
| Botswana | L. pilifolium C.H. Wright |  |  | (Yao et al., 2018) |
| Botswana | L. schizocalyx C.H. Wright |  |  | (Yao et al., 2018) |
| Botswana | L. shawii Roem. & Schult. | food | medicine | (Yao et al., 2018) |
| Botswana | L. villosum Schinz |  |  | (Yao et al., 2018) |
| Brazil | L. cestroides Schltdl. |  | medicine | (Rondina et al., 2008) |
| Brazil | L. chilense Bertero |  | medicine | (Yao et al., 2018) |
| Brazil | L. ciliatum Schltdl. |  | medicine | (Trillo et al., 2010) |
| Brazil | L. glomeratum Sendtn. |  |  | (Yao et al., 2018) |
| Brazil | L. martii Sendtn. |  |  | (Yao et al., 2018) |
| Caicos Islands | L. infaustum Miers |  |  | (Yao et al., 2018) |
| Caicos Islands | L. tweedianum Griseb. | food |  | (Roth and Lindorf, 2013) |
| Chile | L. chanar Phil. |  |  | (Aguilar et al., 2002) |
| Chile | L. chilense Bertero |  | medicine | (Levin and Miller, 2005a) |
| Chile | L. deserti Phil. |  |  | (Pinto et al., 2006) |
| Chile | L. distichum Meyen |  |  | (Montesinos-Tubée, 2016) |
| Chile | L. gilliesianum Miers |  |  | (Yao et al., 2018) |
| Chile | L. humile Phil. |  |  | (Yao et al., 2018) |
| Chile | L. leiostemum Wedd. |  |  | (Yao et al., 2018) |
| Chile | L. minutifolium Remy |  |  | (Yao et al., 2018) |
| Chile | L. rachidocladum Dunal |  |  | (Yao et al., 2018) |
| Chile | L. stenophyllum J. Rémy |  |  | (Yao et al., 2018) |
| Chile | L. tenuispinosum S.B. Jones & W.Z. Faust |  |  | (Yao et al., 2018) |
| China | L. chinense var. potaninii (Pojark.)A.M.Lu |  | medicine | (Yao et al., 2018) |
| China | L. cylindricum Kuang & A. M. Lu |  |  | (Li et al., 2014) |
| China | L. dasystemum Pojark. | food | medicine | (WANG et al., 2013) |
| China | L. glomeratum Sendtn. |  |  | (Yao et al., 2018) |
| China | L. ruthenicum Murray | food | medicine | (Wang et al., 2018) |
| China | L. truncatum Y.C. Wang |  | medicine | (Editorial Committee of Chinese Flora, Chinese Academy of Sciences, 1978) |
| China | L. yunnanense Kuang & A.M. Lu |  |  | (ZHAO et al., 2018) |
| Colombia | L. americanum Jacq. | food |  | (ARENAS and SCARPA, 2007) |
| Colombia | L. infaustum Miers |  |  | (Yao et al., 2018) |
| Colombia | L. tweedianum Griseb. | food |  | (Roth and Lindorf, 2013) |
| Costa Rica | L. americanum Jacq. | food |  | (ARENAS and SCARPA, 2007) |
| Cuba | L. americanum Jacq. | food |  | (ARENAS and SCARPA, 2007) |
| Cuba | L. carolinianum Walter (Moc. & Sessé ex Dunal) C.L.Hitchc | food |  | (Pipoly III and Granson, 2013) |
| Cuba | L. martii Sendtn. |  |  | (Yao et al., 2018) |
| Cuba | L. tweedianum Griseb. | food |  | (Roth and Lindorf, 2013) |
| Cyprus | L. schweinfurthii Dammer |  | medicine | (Ralf, 2000) |
| Daitou Island | L. sandwicense A. Gray | food |  | (Levin and Miller, 2005a) |
| Dominican | L. infaustum Miers |  |  | (Yao et al., 2018) |
| Dominican | L. tweedianum Griseb. | food |  | (Roth and Lindorf, 2013) |
| Dominican Republic | L. americanum Jacq. | food |  | (ARENAS and SCARPA, 2007) |
| Easter Island | L. carolinianum Walter (Moc. & Sessé ex Dunal) C.L.Hitchc | food |  | (Levin and Miller, 2021) |
| Easter Island | L. sandwicense A. Gray | food |  | (Blank et al., 2014) |
| Ecuador | L. americanum Jacq. | food |  | (ARENAS and SCARPA, 2007) |
| Ecuador | L. chilense Bertero |  | medicine | (Yao et al., 2018) |
| Ecuador | L. infaustum Miers |  |  | (Yao et al., 2018) |
| Ecuador | L. minimum C.L. Hitchc. |  |  | (Yao et al., 2018) |
| Ecuador | L. nodosum Miers |  |  | (Yao et al., 2018) |
| Ecuador | L. tweedianum Griseb. | food |  | (Roth and Lindorf, 2013) |
| Egypt | L. europaeum L. | food | medicine | (Zahran and El-Amier, 2013) |
| Egypt | L. intricatum Boiss |  | medicine | (Yao et al., 2018) |
| Egypt | L. schweinfurthii Dammer |  | medicine | (Mamdouh et al., 2021) |
| Egypt | L. shawii Roem. & Schult. | food | medicine | (Soltan and Zaki, 2009) |
| Ethiopia | L. shawii Roem. & Schult. | food | medicine | (Seifu et al., 2006) |
| Europe | L. barbarum L. | food | medicine | (Yao et al., 2018) |
| Europe | L. chinense Mill. | food | medicine | (Yao et al., 2018) |
| France | L. afrum L. | food | medicine | (Dhar et al., 2011; Yao et al., 2018) |
| France | L. chilense Bertero |  | medicine | (Yao et al., 2018) |
| France | L. europaeum L. | food | medicine | (Yao et al., 2018) |
| Georgia | L. ruthenicum Murray | food | medicine | (Yao et al., 2018) |
| Germany | L. afrum L. | food | medicine | (Dhar et al., 2011; Yao et al., 2018) |
| Germany | L. berlandieri Dunal | food | medicine | (Yao et al., 2018) |
| Germany | L. cestroides Schltdl. |  | medicine | (Rondina et al., 2008) |
| Greece | L. schweinfurthii Dammer |  | medicine | (Erwin et al., 2001) |
| Haiti | L. americanum Jacq. | food |  | (ARENAS and SCARPA, 2007) |
| Hawaiian Islands | L. sandwicense A. Gray | food |  | (Blank et al., 2014) |
| India | L. edgeworthii Miers |  |  | (Yao et al., 2018) |
| India | L. europaeum L. | food | medicine | (Kumar and Joshi, 1972) |
| India | L. ruthenicum Murray | food | medicine | (Editorial Committee of Chinese Flora, Chinese Academy of Sciences, 1978) |
| Iran | L. dasystemum Pojark. | food | medicine | (AZADI et al., 2007) |
| Iran | L. depressum Stocks |  | medicine | (Tabaraki et al., 2013) |
| Iran | L. edgeworthii Miers |  |  | (Yao et al., 2018) |
| Iran | L. horridum Thunb. |  |  | (Yao et al., 2018) |
| Iran | L. ruthenicum Murray | food | medicine | (Editorial Committee of Chinese Flora, Chinese Academy of Sciences, 1978) |
| Iraq | L. depressum Stocks |  | medicine | (Ghasemi et al., 2013) |
| Islasde Barlovento | L. americanum Jacq. | food |  | (ARENAS and SCARPA, 2007) |
| Israel | L. depressum Stocks |  | medicine | (Yao et al., 2018) |
| Israel | L. europaeum L. | food | medicine | (Yao et al., 2018) |
| Israel | L. schweinfurthii Dammer |  | medicine | (Abou Auda, 2011) |
| Israel | L. shawii Roem. & Schult. | food | medicine | (Yao et al., 2018) |
| Italy | L. intricatum Boiss |  | medicine | (Yao et al., 2018) |
| Italy | L. petraeum Feinbrun |  |  | (Yao et al., 2018) |
| Jamaica | L. californicum A. Gray |  |  | (Yao et al., 2018) |
| Jamaica | L. carinatum S. Watson |  |  | (Yao et al., 2018) |
| Jamaica | L. infaustum Miers |  |  | (Yao et al., 2018) |
| Jamaica | L. tweedianum Griseb. | food |  | (Roth and Lindorf, 2013) |
| Jordan | L. depressum Stocks |  | medicine | (Yao et al., 2018) |
| Japan | 1. chinense Mill | food | medicine | (Editorial Committee of Chinese Flora, 1978) |
| Jordan | L. petraeum Feinbrun |  |  | (Yao et al., 2018) |
| Jordan | L. shawii Roem. & Schult. | food | medicine | (Yao et al., 2018) |
| Lesotho | L. acutifolium E. Mey. ex Dunal | food | medicine | (D'Arcy, 1992; Dhar et al., 2011) |
| Lesotho | L. arenicolum Miers |  |  | (Yao et al., 2018) |
| Lesotho | L. cinereum Thunb. | food | medicine | (Yao et al., 2018) |
| Lesotho | L. ferocissimum Miers | food | medicine | (Yao et al., 2018) |
| Lesotho | L. horridum Thunb. |  |  | (Yao et al., 2018) |
| Madagascar | L. acutifolium E. Mey. ex Dunal | food | medicine | (D'Arcy, 1992; Dhar et al., 2011) |
| Madagascar | L. horridum Thunb. |  |  | (Yao et al., 2018) |
| Madagascar | L. mascarenense A.M. Venter & A.J. Scott |  |  | (Yao et al., 2018) |
| Mauritania | L. intricatum Boiss |  | medicine | (Yao et al., 2018) |
| Mauritania | L. schweinfurthii Dammer |  | medicine | (Yao et al., 2018) |
| Mauritius | L. horridum Thunb. |  |  | (Yao et al., 2018) |
| Mauritius | L. mascarenense A.M. Venter & A.J. Scott |  |  | (Yao et al., 2018) |
| Mauritius | L. minutifolium Remy |  |  | (Yao et al., 2018) |
| medieval Cairo | L. afrum L. | food | medicine | (Dhar et al., 2011; Yao et al., 2018) |
| Mexico | L. andersonii A. Gray | food |  | (Saunders, 1920) |
| Mexico | L. andersonii var. deserticola (C.L.Hitchc.) Jeps. |  |  | (Yao et al., 2018) |
| Mexico | L. berlandieri Dunal | food | medicine | (Moran-Palacio et al., 2014) |
| Mexico | L. berlandieri var. parviflorum (A. Gray) A. Terracc. | food | medicine | (Hodgson, 2001) |
| Mexico | L. brevipes Benth. |  |  | (Peinado et al., 2005) |
| Mexico | L. californicum A. Gray |  |  | (Levin et al., 2009) |
| Mexico | L. carinatum S. Watson |  |  | (Yao et al., 2018) |
| Mexico | L. carolinianum Walter (Moc. & Sessé ex Dunal) C.L.Hitchc | food |  | (Levin et al., 2009) |
| Mexico | L. cooperi A. Gray |  |  | (Miller, 2002) |
| Mexico | L. densifolium Wiggins |  |  | (Yao et al., 2018) |
| Mexico | L. dispermum Wiggins |  |  | (Fernando, 1982) |
| Mexico | L. exsertum A. Gray | food |  | (Miller, 2002) |
| Mexico | L. fremontii A. Gray | food |  | (Levin et al., 2009) |
| Mexico | L. isthmense F. Chiang |  |  | (Yao et al., 2018) |
| Mexico | L. leiostemum Wedd. |  |  | (Yao et al., 2018) |
| Mexico | L. macrodon A. Gray |  |  | (Yao et al., 2018) |
| Mexico | L. megacarpum Wiggins |  |  | (Yao et al., 2018) |
| Mexico | L. nodosum Miers |  |  | (Yao et al., 2018) |
| Mexico | L. pallidum Miers | food | medicine | (Yao et al., 2018) |
| Mexico | L. parishii A. Gray | food |  | (Savage and Miller, 2006) |
| Mexico | L. parishii var. modestum (I.M. Johnst.)F. Chiang |  |  | (Yao et al., 2018) |
| Mexico | L. puberulum A. Gray |  |  | (Yao et al., 2018) |
| Mexico | L. pubitubum C.L.Hitchc. |  |  | (Yao et al., 2018) |
| Mexico | L. richii A. Gray | food |  | (Hodgson, 2001) |
| Mexico | L. ruthenicum Murray | food | medicine | (Yao et al., 2018) |
| Mexico | L. shockleyi A. Gray |  |  | (Yao et al., 2018) |
| Mexico | L. texanum Correll |  |  | (Yao et al., 2018) |
| Mexico | L. torreyi A. Gray | food | medicine | (Miller, 2002) |
| Morocco | L. ferocissimum Miers | food | medicine | (Noble et al., 2021) |
| Morocco | L. intricatum Boiss |  | medicine | (Midhat et al., 2019) |
| Morocco | L. schweinfurthii Dammer |  | medicine | (Mahklouf, 2020) |
| Mozambique | L. mascarenense A.M. Venter & A.J. Scott |  |  | (Yao et al., 2018) |
| Mozambique | L. schizocalyx C.H. Wright |  |  | (Yao et al., 2018) |
| Namibia | L. amoenum Dammer |  |  | (Yao et al., 2018) |
| Namibia | L. bosciifolium Schinz | food |  | (Ndithia and Perrin, 2006) |
| Namibia | L. cinereum Thunb. | food | medicine | (Yao et al., 2018) |
| Namibia | L. decumbens Welw. ex Hiern |  |  | (Levin et al., 2006) |
| Namibia | L. eenii S. Moore |  |  | (Yao et al., 2018) |
| Namibia | L. ferocissimum Miers | food | medicine | (Yao et al., 2018) |
| Namibia | L. gariepense A.M.Venter |  |  | (Yao et al., 2018) |
| Namibia | L. grandicalyx Joubert & Venter |  |  | (Yao et al., 2018) |
| Namibia | L. hirsutum Dunal |  |  | (Yao et al., 2018) |
| Namibia | L. horridum Thunb. |  |  | (Yao et al., 2018) |
| Namibia | L. oxycarpum Dunal |  | medicine | (Mennicken et al., 2005) |
| Namibia | L. pilifolium C.H. Wright |  |  | (Joubert et al., 1984) |
| Namibia | L. prunus-spinosa Dunal |  | medicine | (Joubert and Venter, 1989) |
| Namibia | L. pumilum Dammer |  |  | (Yao et al., 2018) |
| Namibia | L. schizocalyx C.H. Wright |  |  | (Joubert and Venter, 1989) |
| Namibia | L. tetrandrum Thunb. | food |  | (Watt and Breyer-Brandwijk, 1986) |
| Namibia | L. villosum Schinz |  |  | (Minne et al., 1994) |
| Netherlands | L. afrum L. | food | medicine | (Dhar et al., 2011; Yao et al., 2018) |
| New Zealand | L. ferocissimum Miers | food | medicine | (Ireland et al., 2019) |
| Norfolk Island | L. ferocissimum Miers | food | medicine | (Noble et al., 2021) |
| North America | L. barbarum L. | food | medicine | (Kocyigit and Sanlier, 2017) |
| North America | L. chinense Mill. | food | medicine | (Yao et al., 2018) |
| Ogasawara Islands | L. sandwicense A. Gray | food |  | (Fukuda et al., 2001) |
| North Korea | L. chinense Mill. | Food | Medicine | (Editorial Committee of Chinese Flora, 1978) |
| Oman | L. shawii Roem. & Schult. | food | medicine | (Yao et al., 2018) |
| Pakistan | L. depressum Stocks |  | medicine | (Yao et al., 2018) |
| Pakistan | L. edgeworthii Miers |  |  | (Yao et al., 2018) |
| Pakistan | L. makranicum Schonebeck-Temesy |  |  | (Yao et al., 2018) |
| Pakistan | L. ruthenicum Murray | food | medicine | (Editorial Committee of Chinese Flora, Chinese Academy of Sciences, 1978) |
| Palestinian | L. depressum Stocks |  | medicine | (Yao et al., 2018) |
| Palestinian Territory | L. europaeum L. | food | medicine | (Yao et al., 2018) |
| Palestinian Territory | L. shawii Roem. & Schult. | food | medicine | (Yao et al., 2018) |
| Paraguay | L. americanum Jacq. | food |  | (ARENAS and SCARPA, 2007) |
| Paraguay | L. chilense Bertero |  | medicine | (Yao et al., 2018) |
| Paraguay | L. cuneatum Dammer |  |  | (Yao et al., 2018) |
| Paraguay | L. glomeratum Sendtn. |  |  | (Yao et al., 2018) |
| Paraguay | L. infaustum Miers |  |  | (Yao et al., 2018) |
| Paraguay | L. morongii Britton |  |  | (Yao et al., 2018) |
| Paraguay | L. nodosum Miers |  |  | (Yao et al., 2018) |
| Paraguay | L. tenuispinosum S.B. Jones & W.Z. Faust |  |  | (Yao et al., 2018) |
| Paraguay | L. tweedianum Griseb. | food |  | (Roth and Lindorf, 2013) |
| Peru | L. americanum Jacq. | food |  | (ARENAS and SCARPA, 2007) |
| Peru | L. distichum Meyen |  |  | (Montesinos-Tubée, 2016) |
| Peru | L. divaricatum Rusby |  |  | (Yao et al., 2018) |
| Peru | L. infaustum Miers |  |  | (Yao et al., 2018) |
| Peru | L. leiostemum Wedd. |  |  | (Yao et al., 2018) |
| Peru | L. nodosum Miers |  |  | (Yao et al., 2018) |
| Peru | L. stenophyllum J. Rémy |  |  | (Yao et al., 2018) |
| Portugal | L. europaeum L. | food | medicine | (Dahmani et al., 2020) |
| Portugal | L. infaustum Miers |  |  | (Yao et al., 2018) |
| Portugal | L. intricatum Boiss |  | medicine | (Lombardo et al., 2020) |
| Portugal | L. schweinfurthii Dammer |  | medicine | (Yao et al., 2018) |
| Reunion | L. mascarenense A.M. Venter & A.J. Scott |  |  | (Yao et al., 2018) |
| Russia | L. depressum Stocks |  | medicine | (Yao et al., 2018) |
| Russian | L. ruthenicum Murray | food | medicine | (Editorial Committee of Chinese Flora, Chinese Academy of Sciences, 1978) |
| Saudi Arabia | L. intricatum Boiss |  | medicine | (Yao et al., 2018) |
| Saudi Arabia | L. shawii Roem. & Schult. | food | medicine | (Ghazanfar, 1994) |
| South Africa | L. acutifolium E. Mey. ex Dunal | food | medicine | (Joubert et al., 1984; Dhar et al., 2011) |
| South Africa | L. afrum L. | food | medicine | (Dhar et al., 2011; Yao et al., 2018) |
| South Africa | L. amoenum Dammer |  |  | (Yao et al., 2018) |
| South Africa | L. arenicolum Miers |  |  | (Yao et al., 2018) |
| South Africa | L. bosciifolium Schinz | food |  | (Ndithia and Perrin, 2006) |
| South Africa | L. cinereum Thunb. | food | medicine | (Rubin and Palmer, 1996) |
| South Africa | L. decumbens Welw. ex Hiern |  |  | (Levin et al., 2006) |
| South Africa | L. ferocissimum Miers | food | medicine | (McCulloch et al., 2020) |
| South Africa | L. gariepense A.M.Venter |  |  | (Yao et al., 2018) |
| South Africa | L. grandicalyx Joubert & Venter |  |  | (Yao et al., 2018) |
| South Africa | L. hantamense A.M.Venter |  |  | (Yao et al., 2018) |
| South Africa | L. hirsutum Dunal |  |  | (Yao et al., 2018) |
| South Africa | L. horridum Thunb. |  |  | (Yao et al., 2018) |
| South Africa | L. mascarenense A.M. Venter & A.J. Scott |  |  | (Yao et al., 2018) |
| South Africa | L. oxycarpum Dunal |  | medicine | (Mennicken et al., 2005) |
| South Africa | L. pilifolium C.H. Wright |  |  | (Joubert et al., 1984) |
| South Africa | L. prunus-spinosa Dunal |  | medicine | (Joubert and Venter, 1989) |
| South Africa | L. pumilum Dammer |  |  | (Yao et al., 2018) |
| South Africa | L. schizocalyx C.H. Wright |  |  | (Joubert et al., 1984) |
| South Africa | L. shawii Roem. & Schult. | food | medicine | (Yao et al., 2018) |
| South Africa | L. strandveldense A.M. Venter |  |  | (Yao et al., 2018) |
| South Africa | L. tetrandrum Thunb. | food |  | (Watt and Breyer-Brandwijk, 1986) |
| South Africa | L. villosum Schinz |  |  | (Minne et al., 1994) |
| South America | L. barbarum L. | food | medicine | (Yao et al., 2018) |
| Spain | L. europaeum L. | food | medicine | (Yang et al., 2015) |
| South Korea | Lycium chinense Mill | food | medicine | (Editorial Committee of Chinese Flora, 1978) |
| Spain | L. ferocissimum Miers | food | medicine | (Latorre et al., 2006) |
| Spain | L. intricatum Boiss |  | medicine | (Carrión, 2002) |
| Spain | L. schweinfurthii Dammer |  | medicine | (Yao et al., 2018) |
| Sweden | L. afrum L. | food | medicine | (Dhar et al., 2011; Yao et al., 2018) |
| Switzerland | L. chilense Bertero |  | medicine | (Yao et al., 2018) |
| Territory | L. depressum Stocks |  | medicine | (Yao et al., 2018) |
| Tuks | L. tweedianum Griseb. | food |  | (Roth and Lindorf, 2013) |
| Tunisia | L. afrum L. | food | medicine | (Dhar et al., 2011; Yao et al., 2018) |
| Tunisia | L. europaeum L. | food | medicine | (Tej et al., 2018) |
| Tunisia | L. ferocissimum Miers | food | medicine | (Yao et al., 2018) |
| Tunisia | L. intricatum Boiss |  | medicine | (Abdennacer et al., 2015) |
| Tunisia | L. schweinfurthii Dammer |  | medicine | (Mamdouh et al., 2021) |
| Turkey | L. anatolicum A.Baytop & R.R.Mill |  |  | (Yao et al., 2018) |
| Turkey | L. depressum Stocks |  | medicine | (Yao et al., 2018) |
| Turkey | L. horridum Thunb. |  |  | (Yao et al., 2018) |
| Turkmenistan | L. depressum Stocks |  | medicine | (Yao et al., 2018) |
| Turkmenistan | L. ruthenicum Murray | food | medicine | (Yao et al., 2018) |
| Turks | L. infaustum Miers |  |  | (Yao et al., 2018) |
| UK | L. cestroides Schltdl. |  | medicine | (Rondina et al., 2008) |
| UK | L. chilense Bertero |  | medicine | (Yao et al., 2018) |
| United States | L. arenicolum Miers |  |  | (Yao et al., 2018) |
| Uruguay | L. cestroides Schltdl. |  | medicine | (Rondina et al., 2008) |
| Uruguay | L. vimineum Miers |  |  | (Yao et al., 2018) |
| US | L. andersonii A. Gray | food |  | (Saunders, 1920) |
| US | L. andersonii var. deserticola (C.L.Hitchc.) Jeps. |  |  | (Hodgson, 2001) |
| US | L. berberioides Correll |  |  | (Yao et al., 2018) |
| US | L. berlandieri Dunal | food | medicine | (Newton, 2012) |
| US | L. berlandieri var. parviflorum (A. Gray) A. Terracc. | food | medicine | (Hodgson, 2001) |
| US | L. brevipes Benth. |  |  | (Björk et al., 2007) |
| US | L. californicum A. Gray |  |  | (Levin et al., 2009) |
| US | L. cooperi A. Gray |  |  | (Miller, 2002) |
| US | L. exsertum A. Gray | food |  | (Miller, 2002) |
| US | L. ferocissimum Miers | food | medicine | (Weretilnyk et al., 1989) |
| US | L. fremontii A. Gray | food |  | (Levin et al., 2009) |
| US | L. hassei Greene |  |  | (Yao et al., 2018) |
| US | L. macrodon A. Gray |  |  | (Yao et al., 2018) |
| US | L. oxycarpum Dunal |  | medicine | (Yao et al., 2018) |
| US | L. pallidum Miers | food | medicine | (Strojan et al., 1979) |
| US | L. parishii A. Gray | food |  | (Savage and Miller, 2006) |
| US | L. puberulum A. Gray |  |  | (Yao et al., 2018) |
| US | L. pubitubum C.L.Hitchc. |  |  | (Yao et al., 2018) |
| US | L. repens Speg |  |  | (Yao et al., 2018) |
| US | L. richii A. Gray | food |  | (Hodgson, 2001) |
| US | L. shockleyi A. Gray |  |  | (Yao et al., 2018) |
| US | L. texanum Correll |  |  | (Yao et al., 2018) |
| US | L. torreyi A. Gray | food | medicine | (Miller, 2002) |
| US | L. verrucosum Eastw. |  |  | (Rejmánek, 2018) |
| US, | L. carolinianum Walter (Moc. & Sessé ex Dunal) C.L.Hitchc | food |  | (Levin et al., 2009) |
| Venezuela | L. americanum Jacq. | food |  | (ARENAS and SCARPA, 2007) |
| Venezuela | L. nodosum Miers |  |  | (Yao et al., 2018) |
| Virgin Island | L. tweedianum Griseb. | food |  | (Roth and Lindorf, 2013) |
| West Indies | L. carolinianum Walter (Moc. & Sessé ex Dunal) C.L.Hitchc | food |  | (CHIANG-CABRERA, 1981) |
| Yemen | L. shawii Roem. & Schult. | food | medicine | (Yao et al., 2018) |
| Zimbabwe | L. bosciifolium Schinz | food |  | (Ndithia and Perrin, 2006) |

**References:**

Abdennacer, B., Karim, M., Yassine, M.R., Nesrine, R., and Mouna, D., et al. (2015). Determination of phytochemicals and antioxidant activity of methanol extracts obtained from the fruit and leaves of tunisian lycium intricatum boiss. *Food Chemistry* 174, 577-584. doi:https://doi.org/10.1016/j.foodchem.2014.11.114

Abou Auda, M. (2011). An ethnobotanical use of plants in the middle area, gaza strip, palestine. *Adv Environ Biol* 5, 3681-3688.

Aguilar, R., Bernardello, G., and Galetto, L. (2002). Pollen–pistil relationships and pollen size-number trade-off in species of the tribe lycieae (solanaceae). *Journal of Plant Research* 115, 335-340.

ARENAS, P., and SCARPA, G.F. (2007). Edible wild plants of the chorote indians, gran chaco, argentina. *Botanical Journal of the Linnean Society* 153, 73-85. doi:10.1111/j.1095-8339.2007.00576.x

Arnold, T.H., Prentice, C.A., Hawker, L.C., Snyman, E.E., and Tomalin, M., et al. (2002). *Medicinal and magical plants of southern africa: an annotated checklist.* National Botanical Institute.

AZADI, N., NAZERI, V., NAMAKI SHOUSHTARI, A.A.H., and KAZEM POUR OUSALOU, S. (2007). Lycium dasystemum pojark. (Solanaceae), a new record from iran. *The Iranian Journal of Botany* 13, 109-111.

Bendjedou, H., Barboni, L., Maggi, F., Bennaceur, M., and Benamar, H. (2021). Alkaloids and sesquiterpenes from roots and leaves of lycium europaeum l. (Solanaceae) with antioxidant and anti-acetylcholinesterase activities. *Nat Prod Res* 35, 2784-2788. doi:10.1080/14786419.2019.1666386

Björk, C., Hall, R., McIntosh, T., Riefner, R.E., and Murdock, A.G., et al. (2007). Noteworthy collections. *Madroño* 54, 366-370.

Blank, C.M., Levin, R.A., and Miller, J.S. (2014). Intraspecific variation in gender strategies in lycium (solanaceae): associations with ploidy and changes in floral form following the evolution of gender dimorphism. *American Journal of Botany* 101, 2160-2168. doi:https://doi.org/10.3732/ajb.1400356

Carrión, J.S. (2002). A taphonomic study of modern pollen assemblages from dung and surface sediments in arid environments of spain. *Review of Palaeobotany and Palynology* 120, 217-232. doi:https://doi.org/10.1016/S0034-6667(02)00073-8

CHIANG-CABRERA, F. (1981). "A taxonomic study of the north american species of lycium (solanaceae)". (The University of Texas at Austin: Ann Arbor).

Dahmani, J., Benharbit, M., Fassar, M., Hajila, R., and Zidane, L., et al. (2020). Vascular plants census linked to the biodeterioration process of the portuguese city of mazagan in el jadida, morocco. *Journal of King Saud University - Science* 32, 682-689. doi:https://doi.org/10.1016/j.jksus.2018.10.015

Dalmasso, A.D., and Márquez, J. (2018). Relevamiento de la vegetación del área de castaño nuevo-villa corral, calingasta, provincia de san juan, argentina. *Chloris Chilensis* 21.

D'Arcy, W.G. (1992). Solanaceae of madagascar: form and geography. *Annals of the Missouri Botanical Garden* 79, 29-45. doi:10.2307/2399807

Dhar, P., Tayade, A., Ballabh, B., Chaurasia, O.P., and Bhatt, R.P., et al. (2011). Lycium ruthenicum murray: a less-explored but high-value medicinal plant from trans-himalayan cold deserts of ladakh, india. *Plant Archives* 11, 583-586.

Editorial Committee of Chinese Flora, Chinese Academy of Sciences (1978). *Flora of china*. science press: Beijing.

Erwin, B., Zacharias, K., Ralf, J., Niels, B., and Panayotis, D., et al. (2001). Flora and phytogeographical significance of the islands chrisi, koufonisi and nearby islets (s aegean, greece). *Willdenowia* 31, 329-356. doi:10.3372/wi.31.31205

Fernando, C. (1982). New combinations and new variety of lycium (solanaceae) from north america. *Botanical Sciences* 0. doi:10.17129/botsci.1263

Fukuda, T., Yokoyama, J., and Ohashi, H. (2001). Phylogeny and biogeography of the genus lycium (solanaceae): inferences from chloroplast dna sequences. *Molecular Phylogenetics and Evolution* 19, 246-258. doi:https://doi.org/10.1006/mpev.2001.0921

Ghasemi, P.A., Momeni, M., and Bahmani, M. (2013). Ethnobotanical study of medicinal plants used by kurd tribe in dehloran and abdanan districts, ilam province, iran. *African Journal of Traditional, Complementary and Alternative Medicines* 10, 368-385.

Ghazanfar, S.A. (1994). *Handbook of arabian medicinal plants*. CRC press.

Gong, G., Fan, J., Sun, Y., Wu, Y., and Liu, Y., et al. (2016). Isolation, structural characterization, and antioxidativity of polysaccharide lblp5-a from lycium barbarum leaves. *Process Biochemistry* 51, 314-324.

Hitchcock, C.L. (1932). A monographic study of the genus lycium of the western hemisphere. *Annals of the Missouri Botanical Garden* 19, 179-374. doi:10.2307/2394155

Hodgson, W.C. (2001). *Food plants of the sonoran desert*. University of Arizona Press.

Ireland, K.B., Hunter, G.C., Wood, A., Delaisse, C., and Morin, L. (2019). Evaluation of the rust fungus puccinia rapipes for biological control of lycium ferocissimum (african boxthorn) in australia: life cycle, taxonomy and pathogenicity. *Fungal Biology* 123, 811-823. doi:https://doi.org/10.1016/j.funbio.2019.08.007

Jeanes, J.A. (1999). "Goodeniaceae in: walsh ng, entwisle tj (eds) flora of victoria vol. 4, cornaceae to asteraceae". (Inkata Press, Melbourne).

Joubert, A.M., Verhoeven, R.L., and Venter, H.J.T. (1984). An anatomical investigation of the stem and leaf of the south african species of lycium l. (Solanaceae). *South African Journal of Botany* 3, 219-230. doi:https://doi.org/10.1016/S0022-4618(16)30033-X

Joubert, A.M., and Venter, H.J.T. (1989). A new species of lycium (solanaceae) from namibia. *South African Journal of Botany* 55, 516-522. doi:https://doi.org/10.1016/S0254-6299(16)31149-8

Kocyigit, E., and Sanlier, N. (2017). A review of composition and health effects of lycium barbarum. *International Journal of Chinese Medicine* 1, 1.

Kumar, A., and Joshi, M.C. (1972). The effects of grazing on the structure and productivity of the vegetation near pilani, rajasthan, india. *Journal of Ecology* 60, 665-674. doi:10.2307/2258558

Latorre, A.V.P., Ramos, R.Y., and Sánchez, E.D. (2006). Lycium ferocissimum miers en la península ibérica (málaga, españa). *Acta Botanica Malacitana* 31, 208.

Levin, R.A., Blanton, J., and Miller, J.S. (2009). Phylogenetic utility of nuclear nitrate reductase: a multi-locus comparison of nuclear and chloroplast sequence data for inference of relationships among american lycieae (solanaceae). *Molecular Phylogenetics and Evolution* 50, 608-617. doi:https://doi.org/10.1016/j.ympev.2008.12.005

Levin, R.A., Shak, J.R., Miller, J.S., Bernardello, G., and Venter, A.M. (2006). "Evolutionary relationships in tribe lycieae (solanaceae)". In: *VI International Solanaceae Conference: Genomics Meets Biodiversity 745* (ed.).).

Levin, R.A., Whelan, A., and Miller, J.S. (2009). The utility of nuclear conserved ortholog set ii (cosii) genomic regions for species-level phylogenetic inference in lycium (solanaceae). *Molecular Phylogenetics and Evolution* 53, 881-890. doi:https://doi.org/10.1016/j.ympev.2009.08.016

Levin, R.A., and Miller, J.S. (2005a). Relationships within tribe lycieae (solanaceae): paraphyly of lycium and multiple origins of gender dimorphism. *American Journal of Botany* 92, 2044-2053. doi:https://doi.org/10.3732/ajb.92.12.2044

Levin, R.A., and Miller, J.S. (2005b). Relationships within tribe lycieae (solanaceae): paraphyly of lycium and multiple origins of gender dimorphism. *American Journal of Botany* 92, 2044-2053. doi:https://doi.org/10.3732/ajb.92.12.2044

Levin, R.A., and Miller, J.S. (2021). Molecular signatures of long‐distance oceanic dispersal and the colonization of pacific islands in lycium carolinianum. *American Journal of Botany* 108, 694-710.

Li, W., Guan, K., Turland, N.J., and Wiersema, J.H. (2014). (021–022) proposals to amend art. 40 note 2 and add a new example. *Taxon* 63, 693. doi:10.12705/633.12

Lombardo, E., Bancheva, S., Domina, G., and Venturella, G. (2020). Distribution, ecological role and symbioses of selected shrubby species in the mediterranean basin: a review. *Plant Biosystems-an International Journal Dealing with All Aspects of Plant Biology* 154, 438-454.

Mahklouf, M.H. (2020). Biodiversity of the coastal flora of tripoli province. *Biodiversity Research and Conservation* 58, 13-19. doi:10.2478/biorc-2020-0006

Mamdouh, D., Mahgoub, H.A.M., Gabr, A.M.M., Ewais, E.A., and Smetanska, I. (2021). Genetic stability, phenolic, flavonoid, ferulic acid contents, and antioxidant activity of micropropagated lycium schweinfurthii plants. *Plants* 10. doi:10.3390/plants10102089

McCulloch, G.A., Mauda, E.V., Chari, L.D., Martin, G.D., and Gurdasani, K., et al. (2020). Genetic diversity and morphological variation in african boxthorn (lycium ferocissimum) – characterising the target weed for biological control. *Biological Control* 143, 104206. doi:https://doi.org/10.1016/j.biocontrol.2020.104206

Mennicken, M., Maier, W., and Oberwinkler, F. (2005). A contribution to the rust flora (uredinales) of southern africa, with an emphasis on namibia. *Mycological Progress* 4, 55-75. doi:10.1007/s11557-006-0109-5

Midhat, L., Ouazzani, N., Hejjaj, A., Ouhammou, A., and Mandi, L. (2019). Accumulation of heavy metals in metallophytes from three mining sites (southern centre morocco) and evaluation of their phytoremediation potential. *Ecotoxicology and Environmental Safety* 169, 150-160. doi:https://doi.org/10.1016/j.ecoenv.2018.11.009

Miller, J.S. (2002). Phylogenetic relationships and the evolution of gender dimorphism in lycium (solanaceae). *Systematic Botany* 27, 416-428. doi:10.1043/0363-6445-27.2.416

Minne, L., Spies, J.J., Venter, H., and Venter, A.M. (1994). Breeding systems in some representatives of the genus lycium (solanaceae). *Bothalia* 24, 107-110.

Montesinos-Tubée, D.B. (2016). *The mountain vegetation of south peru: syntaxonomy, ecology, phytogeography and conservation*. Wageningen University.

Moran-Palacio, E.F., Zamora-Álvarez, L.A., Stephens-Camacho, N.A., Yáñez-Farías, G.A., and Virgen-Ortiz, A., et al. (2014). Antioxidant capacity, radical scavenging kinetics and phenolic profile of methanol extracts of wild plants of southern sonora, mexico. *Tropical Journal of Pharmaceutical Research* 13, 1487-1493.

NCN, S.A. (2004). Submitted by omar torres-carvajal, division of herpetology, ku natural history museum and biodiversity re search center, 1345 jayhawkboulevard, lawrence, kansas 66045 7561, usa; E~ mai1: otorres@ ku. Edu. *Herpetological Review* 35, 172.

Ndithia, H., and Perrin, M.R. (2006). Diet and foraging behaviour of the rosy-faced lovebird agapornis roseicollis in namibia. *Ostrich-Journal of African Ornithology* 77, 45-51.

Newton, D.R. (2012) The vascular flora of the eagletail mountain region. Arizona State University.

Noble, M.R., Adair, R.J., and Ireland, K.B. (2021). Biology of invasive plants 2. Lycium ferocissimum miers. *Invasive Plant Science and Management* 14, 41-56. doi:DOI: 10.1017/inp.2021.13

PARK, S., PARK, W.T., PARK, Y.C., JU, J.I., and PARK, S.U., et al. (2012). Metabolomics for the quality assessment of lycium chinense fruits. *Bioscience, Biotechnology, and Biochemistry* 76, 2188-2194. doi:10.1271/bbb.120453

Peinado, M., Delgadillo, J., and Aguirre, J.L. (2005). Plant associations of el vizcaíno biosphere reserve, baja california sur, mexico. *The Southwestern Naturalist* 50, 129-149.

Pinto, R., Barría, I., and Marquet, P.A. (2006). Geographical distribution of tillandsia lomas in the atacama desert, northern chile. *Journal of Arid Environments* 65, 543-552. doi:https://doi.org/10.1016/j.jaridenv.2005.08.015

Pipoly III, J.J., and Granson, S.L. (2013). Shrubs recommended for use as perimeter plantings (informal or formal hedge) in south florida.

Ralf, H. (2000). Contributions to the flora of cyprus i. *Willdenowia* 30, 53-65. doi:10.3372/wi.30.30104

Rejmánek, M. (2018). Vascular plant extinctions in california: a critical assessment. *Diversity and Distributions* 24, 129-136. doi:https://doi.org/10.1111/ddi.12665

Rondina, R.D., Bandoni, A.L., and Coussio, J.D. (2008). Argentine medicinal species with potential analgesic activity. *Dominguezia* 24, 47-69.

Roth, I., and Lindorf, H. (2013). *South american medicinal plants: botany, remedial properties and general use*. Springer Science & Business Media.

Rubin, F., and Palmer, A.R. (1996). The physical environment and major plant communities of the karoo national park, south africa. *Koedoe* 39, 25-52.

Saunders, C.F. (1920). *Useful wild plants of the united states and canada*. New York: RM McBride.

Savage, A.E., and Miller, J.S. (2006). Gametophytic self-incompatibility in lycium parishii (solanaceae): allelic diversity, genealogical structure, and patterns of molecular evolution at the s-rnase locus. *Heredity* 96, 434-444. doi:10.1038/sj.hdy.6800818

Seifu, T., Asres, K., and Gebre-Mariam, T. (2006). Ethnobotanical and ethnopharmaceutical studies on medicinal plants of chifra district, afar region, north eastern ethiopia. *Ethiopian Pharmaceutical Journal* 24, 41-58.

Soltan, M.M., and Zaki, A.K. (2009). Antiviral screening of forty-two egyptian medicinal plants. *Journal of Ethnopharmacology* 126, 102-107. doi:https://doi.org/10.1016/j.jep.2009.08.001

Strojan, C.L., Turner, F.B., and Castetter, R. (1979). Litter fall from shrubs in the northern mojave desert. *Ecology* 60, 891-900. doi:https://doi.org/10.2307/1936857

Sun, Y., Liang, Z., Shan, C., Viernstein, H., and Unger, F. (2011). Comprehensive evaluation of natural antioxidants and antioxidant potentials in ziziphus jujuba mill. Var. Spinosa (bunge) hu ex hf chou fruits based on geographical origin by topsis method. *Food Chemistry* 124, 1612-1619.

Tabaraki, R., Nateghi, A., and Ahmady-Asbchin, S. (2013). In vitro assessment of antioxidant and antibacterial activities of six edible plants from iran. *Journal of Acupuncture and Meridian Studies* 6, 159-162. doi:https://doi.org/10.1016/j.jams.2013.01.016

Tej, R., Rodríguez-Mallol, C., Rodríguez-Arcos, R., Karray-Bouraoui, N., and Molinero-Ruiz, L. (2018). Inhibitory effect of lycium europaeum extracts on phytopathogenic soil-borne fungi and the reduction of late wilt in maize. *European Journal of Plant Pathology* 152, 249-265. doi:10.1007/s10658-018-1469-9

Toledo, B.A., Trillo, C., Grilli, M., Colantonio, S., and Galetto, L. (2014). Relationships between land-use types and plant species used by traditional ethno-medical system. *European Journal of Medicinal Plants*, 998-1021.

Trillo, C., Toledo, B.A., Galetto, L., and Colantonio, S. (2010). Persistence of the use of medicinal plants in rural communities of the western arid chaco [cordoba, argentina]~!2010-01-05~!2010-04-27~!2010-06-22. *The Open Complementary Medicine Journal* 2, 80-89. doi:10.2174/1876391X01002020080

Villavicencio, H.J., Acosta, J.C., and Cánovas, M.G. (2005). Dieta de liolaemus ruibali donoso barros (iguania: liolaeminae) en la reserva de usos múltiples don carmelo, san juan, argentina. *Multequina*, 47-52.

WANG, Z., JIN, K., and GU, L. (2013). Resource investigation and conservation measures of lycium l. In xinjiang. *Northern Horticulture*, 3.

Wang, Z., Yan, Y., Nisar, T., Zou, L., and Yang, X., et al. (2018). Comparison and multivariate statistical analysis of anthocyanin composition in lycium ruthenicum murray from different regions to trace geographical origins: the case of china. *Food Chemistry* 246, 233-241. doi:https://doi.org/10.1016/j.foodchem.2017.11.030

Watt, J.M., and Breyer-Brandwijk, M.G. (1986). *The medicinal and poisonous plants of southern and eastern africa; Eing an account of their medicinal and other uses, chemical composition...* University Microfilms.

Weretilnyk, E.A., Bednarek, S., McCue, K.F., Rhodes, D., and Hanson, A.D. (1989). Comparative biochemical and immunological studies of the glycine betaine synthesis pathway in diverse families of dicotyledons. *Planta* 178, 342-352. doi:10.1007/BF00391862

Yang, T., Dong, J., Yue, J., and Wang, Y. (2015). A new wolfberry cultivar‘zhongke lüchuan 1’. *Acta Horticulturae Sinica* 42, 2557-2558. doi:10.16420/j.issn.0513-353x.2014-1003

Yao, R., Heinrich, M., and Weckerle, C.S. (2018). The genus lycium as food and medicine: a botanical, ethnobotanical and historical review. *Journal of Ethnopharmacology* 212, 50-66. doi:10.1016/j.jep.2017.10.010

Zahran, M.A., and El-Amier, Y.A. (2013). Non-traditional fodders from the halophytic vegetation of the deltaic mediterranean coastal desert, egypt. *J. Biol. Sci* 13, 226-233.

ZHAO, J., LI, H., ZHANG, C., AN, W., and YIN, Y., et al. (2018). Physiological response of four wolfberry (lycium linn.) Species under drought stress. *Journal of Integrative Agriculture* 17, 603-612. doi:https://doi.org/10.1016/S2095-3119(17)61754-4
