# Supplementary material for: Germplasm Resources and Strategy for Genetic Breeding of Lycium Species: A Review
Source: Front Plant Sci. 2022 Feb 11;13:802936. doi: 10.3389/fpls.2022.802936 (PMC8874141; doi:10.3389/fpls.2022.802936)
Supplement: Supplementary file 2 [file Table_2.docx]

**Supplementary File**

**Germplasm Resources and Strategy for Genetic Breeding of *Lycium* species: A Review**

Haiguang Gong1,2, Fazal Rehman1,2, Yun Ma1,2, Biao A1,2, Dongpo Wu, Ying Wang1,2*

1 Key Laboratory of South China Agricultural Plant Molecular Analysis and Genetic Improvement, Provincial Key Laboratory of Applied Botany, South China Botanical Garden, Chinese Academy of Science s, Guangzhou 510650, P.R. China.

2 University of Chinese Academy of Sciences, Beijing 100049, P.R. China.

**Table S2 The** **whole plant related traits**

| No. | **Traits** | **Character** | **Description** | **Reference** |
| --- | --- | --- | --- | --- |
| 1 | thorn | QL | with | (Ministry of Agriculture of China, 2013) |
|  |  |  | without |  |
| 2 | thorn density | QN | number/cm | (Ministry of Agriculture of China, 2013) |
| 3 | plant habit index | QL | erect | SGN(Fernandez-Pozo et al., 2014) & (Shi et al., 2012) |
|  |  |  | semi erect |  |
|  |  |  | decumbent |  |
|  |  |  | spreading |  |
|  |  |  | determinate |  |
|  |  |  | indeterminate |  |
|  |  |  | semi determinate |  |
| 4 | internode length | QN | cm | SGN(Fernandez-Pozo et al., 2014)&(Shi et al., 2012) & (Oguz and Erdogan, 2016) |
| 5 | plant canopy | PQ | fair canopy | SGN(Fernandez-Pozo et al., 2014) |
|  |  |  | good canopy |  |
|  |  |  | poor canopy |  |
| 6 | plant height | QN | cm | (Shi et al., 2012) &(Frary et al., 2003) |
| 7 | growth vigor | PQ | weak | (Shi et al., 2012) |
|  |  |  | intermediate |  |
|  |  |  | strong |  |
| 8 | crown breath | QN | cm | (Shi et al., 2012) |
| 9 | crown height | QN | cm | (Shi et al., 2012) |
| 10 | trunk color | QL | greyish brown | (Shi et al., 2012) |
|  |  |  | reddish brown |  |
|  |  |  | brown |  |
|  |  |  |  |  |
| 11 | thorn color | QL | off white | (Shi et al., 2012) |
|  |  |  | yellowish brown |  |
|  |  |  | dark brown |  |
| 12 | thorn length | QN | cm | (Shi et al., 2012) |
| 13 | firmness of thorn | QL | soft | (Shi et al., 2012) |
|  |  |  | intermediate |  |
|  |  |  | hard |  |
| 14 | color of annual branches | QL | off white | (Shi et al., 2012) |
|  |  |  | yellowish brown |  |
|  |  |  | reddish brown |  |
| 15 | color of perennial branches |  | greyish brown | (State Forestry Administration of China, 2013) |
|  |  |  | yellowish brown |  |
|  |  |  | brown |  |
|  |  |  | dark brown |  |
| 16 | Shoot growth rate | QN | cm/d | (Shi et al., 2012) |
| 17 | branch rigidity | PQ | extremely soft | (Shi et al., 2012) |
|  |  |  | soft |  |
|  |  |  | intermediate |  |
|  |  |  | hard |  |
|  |  |  | extremely soft |  |
| 18 | ratio of one year old branch with fruits | PQ | % | (Shi et al., 2012) |
| 19 | ratio of annual branch with fruits | PQ | % | (Shi et al., 2012) |
| 20 | trunk diameter | QN | mm | (Gong et al., 2019) |
| 21 | shoot size | QN | cm | (Oguz and Erdogan, 2016) |
| 22 | stem diameter | QN | cm | (Oguz and Erdogan, 2016) |
| 23 | branching ability | PQ | extremely weak | (Shi et al., 2012) |
|  |  |  | weak |  |
|  |  |  | intermediate |  |
|  |  |  | strong |  |
|  |  |  | extremely strong |  |
| 24 | root number | QN |  | (Wang et al., 2020) |
| 25 | root length | QN | cm | (Wang et al., 2020) |
| 26 | root diameter | QN | cm | (Wang et al., 2020) |
| 27 | daily growth of new shoots | PQ | cm/d | (Wei et al., 2008) |
| 28 | percentage of drop fruit | QN | % | (Wei et al., 2008) |
| 29 | internode length | QN | cm | (Wei et al., 2008) |
| 30 | the length of bearing shoots | QN | cm | (Qi et al., 2019) |

Abbreviation: QN, quantitative characteristics; QL, qualitative characteristics; PQ, pseudo-qualitative characteristics.

**Table S3 Candidate resistance traits for QTL mapping**

|  | **Traits** | **Character** | | **Description** | **Reference** |
| --- | --- | --- | --- | --- | --- |
| 1 | disease and stress response | | QL | disease resistance | SGN(Fernandez-Pozo et al., 2014) |
|  |  | |  | necrosis |  |
|  |  | |  | wilting |  |
| 2 | Resistance to black fruit (Colletotrichum gloeosporioides Penz) | | PQ | resist | (Shi et al., 2012) |
|  |  | |  | medium |  |
|  |  | |  | susceptible |  |
| 3 | Resistance to Mulberry powdery mildew | | PQ | resist | (Bai et al., 2008) |
|  |  | |  | medium |  |
|  |  | |  | susceptible |  |
| 4 | Resistance to root rot | | PQ | resist | (Cao et al., 1999) |
|  |  | |  | medium |  |
|  |  | |  | susceptible |  |
| 5 | salt stress resistance | | QL |  | (Wu et al., 2015) |
| 6 | Resistance to Arphis | | QL |  | (Xu et al., 2013) |
| 7 | resistance to Paratrioza sinica Yang&Li | | QL |  | (Xu et al., 2013) |
| 8 | resistane to Aceria macrodonis Keifer | | QL |  | (Lin, 2016) |
| 9 | resistance to aceric macrodonis | | QL |  | (Xu et al., 2013) |
| 10 | resistance to jaapiella | | QL |  | (Xu et al., 2013) |
| 11 | resistance to lema decempunctata | | PQ | resistance | (Jin et al., 2016) |
|  |  | |  | medium resistance |  |
|  |  | |  | medium susceptible |  |
|  |  | |  | susceptible |  |

Abbreviation: QN, quantitative characteristics; QL, qualitative characteristics; PQ, pseudo-qualitative characteristics.

**Table S4 Candidate Phenology traits for QTL mapping**

|  | **Traits** | **character** | **Description** | | **reference** |
| --- | --- | --- | --- | --- | --- |
| 1 | date of bud appear | QN | | D/M/Y | (State Forestry Administration of China, 2013) |
| 2 | date of bud blooming in autumn | QN | | D/M/Y | (Shi et al., 2012) |
| 3 | date of bud bursting | QN | | D/M/Y | (Shi et al., 2012) |
| 4 | date of ending bloom | QN | | D/M/Y | (Shi et al., 2012) |
| 5 | date of first blooming | QN | | D/M/Y | (Shi et al., 2012) |
| 6 | date of first fruit harvest | QN | | D/M/Y | (State Forestry Administration of China, 2013) |
| 7 | date of fruit colour turning | QN | | D/M/Y | (Shi et al., 2012) |
| 8 | date of full blooming | QN | | D/M/Y | (Shi et al., 2012) |
| 9 | date of green fruit | QN | | D/M/Y | (Shi et al., 2012) |
| 10 | date of last bloom | QN | | D/M/Y | (Shi et al., 2012) |
| 11 | date of leaf-spreading | QN | | D/M/Y | (State Forestry Administration of China, 2013) |
| 12 | date of planting to field | QN | | D/M/Y | (Shi et al., 2012) |
| 13 | date of sowing | QN | | D/M/Y | (Shi et al., 2012) |
| 14 | Days to flowering | QN | | day | (Frary et al., 2003) |
| 15 | days to fruit ripening | QN | | day | (Li et al., 2015) |
| 16 | duration of flowering | QN | | day | (Nurullayeva et al., 2021) |
| 17 | duration of fruit ripening | QN | | day | (Nurullayeva et al., 2021) |
| 18 | flowering cycle | QN | | day | (Nurullayeva et al., 2021) |
| 19 | frutescence | QN | | D/M/Y | (Shi et al., 2012) |
| 20 | Fruit calyx size | PQ | | very short | (Frary et al., 2003) |
|  |  |  | | short |  |
|  |  |  | | intermediate |  |
|  |  |  | | long |  |
|  |  |  | | very long |  |
| 21 | fruit ripening time | QN | | days from anthesis to ripening | SGN(Fernandez-Pozo et al., 2014) &(Doganlar et al., 2000) |
| 22 | fruit ripening time | QN | | early ripe | SGN(Fernandez-Pozo et al., 2014) |
|  |  |  | | late ripe |  |
| 23 | ripening quantitative change | QN | |  | (Nurullayeva et al., 2021) |
| 24 | ripening time | QN | | D/M/Y | (Nurullayeva et al., 2021) |
| 25 | the first fruit ripening time | QN | | D/M/Y | (Ruangrak et al., 2019) |

Abbreviation: QN, quantitative characteristics; QL, qualitative characteristics; PQ, pseudo-qualitative characteristics.

**Table S5. Fruit related traits and their references**

|  | **Traits** | **Character** | **Description** | **Reference** |
| --- | --- | --- | --- | --- |
| 1 | 100 pod weight |  | g | (Qi et al., 2019) |
| 2 | Antioxidant Activity | QN |  | (Yao et al., 2018a) |
| 3 | Circular | QN |  | (Nankar et al., 2020) |
| 4 | colour index | QN |  | (López Camelo and Gómez, 2004) |
| 5 | Curved height | QN | mm | (Nankar et al., 2020) |
| 6 | distal angle macro 10% | QN |  | (Rodríguez et al., 2010) |
| 7 | distal angle macro 15% | QN |  | (Rodríguez et al., 2010) |
| 8 | distal angle macro 20% | QN |  | (Rodríguez et al., 2010) |
| 9 | distal angle macro 25% | QN |  | (Rodríguez et al., 2010) |
| 10 | distal angle micro 2% | QN |  | (Rodríguez et al., 2010) |
| 11 | distal angle micro 3% | QN |  | (Rodríguez et al., 2010) |
| 12 | distal angle micro 5% | QN |  | (Rodríguez et al., 2010) |
| 13 | Distal eccentricity | QN |  | (Nankar et al., 2020) |
| 14 | distal eccentricity index | QN |  | (Maria et al., 2009) |
| 15 | Distal fruit blockiness | QN |  | (Nankar et al., 2020) |
| 16 | distal fruit end blockiness 10% | QN |  | (Rodríguez et al., 2010) |
| 17 | distal fruit end blockiness 20% | QN |  | (Rodríguez et al., 2010) |
| 18 | distal fruit end blockiness 30% | QN |  | (Rodríguez et al., 2010) |
| 19 | distal fruit end blockiness 5% | QN |  | (Rodríguez et al., 2010) |
| 20 | distal fruit end color (abL value) | QN | average 'a' value | SGN(Fernandez-Pozo et al., 2014) &(Rodríguez et al., 2010) |
|  |  |  | average 'b' value |  |
|  |  |  | average L value |  |
| 21 | distal fruit end color(RGB value) | QN | average RGB blue | SGN(Fernandez-Pozo et al., 2014) & (Rodríguez et al., 2010) |
|  |  |  | average RGB green |  |
|  |  |  | average RGB red |  |
| 22 | distal fruit end shape | QL | indentation | SGN(Fernandez-Pozo et al., 2014) |
|  |  |  | protrusion |  |
| 23 | distance of fruit setting | QN | cm | (Shi et al., 2012) |
| 24 | Eccentricity | QN |  | (Nankar et al., 2020) |
| 25 | eccentricity area index | QN |  | (Maria et al., 2009) |
| 26 | Eccentricity area index | QN |  | (Nankar et al., 2020) |
| 27 | Ellipsoid | QN |  | (Nankar et al., 2020) |
| 28 | endocarp cross section color | QN | average chroma | SGN(Fernandez-Pozo et al., 2014) & (Tilahun et al., 2018) & (López Camelo and Gómez, 2004) |
| 29 | endocarp cross section color | QN | average hue | SGN(Fernandez-Pozo et al., 2014) & (López Camelo and Gómez, 2004) & (Sanjaya et al., 2019) |
| 30 | endocarp cross section color | QN | average luminosity | SGN(Fernandez-Pozo et al., 2014) & (Pieczywek et al., 2018) |
| 31 | endocarp cross section color (abL value) | QN | average 'a' value | SGN(Fernandez-Pozo et al., 2014) &(Rodríguez et al., 2010) |
|  |  |  | average 'b' value |  |
|  |  |  | average 'L' value |  |
| 33 | endocarp cross section color (RGB value) | QN | average RGB blue | SGN(Fernandez-Pozo et al., 2014) &(Rodríguez et al., 2010) |
|  |  |  | average RGB green |  |
|  |  |  | average RGB red |  |
| 34 | endocarp external color | QN | chroma | SGN(Fernandez-Pozo et al., 2014) &(Rodríguez et al., 2010) &(Li et al., 2015) |
| 35 | endocarp external color | QN | hue | SGN(Fernandez-Pozo et al., 2014) &(Rodríguez et al., 2010) & (Li et al., 2015) |
| 36 | endocarp external color | QN | luminosity | SGN(Fernandez-Pozo et al., 2014) &(Rodríguez et al., 2010) &(Li et al., 2015) |
| 37 | endocarp longitudinal section color | QN | chroma | SGN(Fernandez-Pozo et al., 2014) & (Rodríguez et al., 2010) &(Li et al., 2015) |
| 38 | endocarp longitudinal section color | QN | hue | SGN(Fernandez-Pozo et al., 2014) &(Rodríguez et al., 2010) &(Li et al., 2015) |
| 39 | endocarp longitudinal section color | QN | luminosity | SGN(Fernandez-Pozo et al., 2014) &(Rodríguez et al., 2010) &(Li et al., 2015) |
| 40 | endocarp longitudinal section colour (abl value) | QN | average 'a' value | SGN(Fernandez-Pozo et al., 2014) &(Rodríguez et al., 2010) &(Li et al., 2015) |
|  |  |  | average 'b' value |  |
|  |  |  | average 'L' value |  |
| 41 | endocarp longitudinal section color (RGB value) | QN | average RGB blue | SGN(Fernandez-Pozo et al., 2014) & (Rodríguez et al., 2010) &(Li et al., 2015) |
|  |  |  | average RGB green |  |
|  |  |  | average RGB red |  |
| 42 | external color uniformity | QN | percent red % | SGN(Fernandez-Pozo et al., 2014) & (Brewer et al., 2008) |
|  |  |  | percent yellow % | SGN(Fernandez-Pozo et al., 2014) &(Brewer et al., 2008) |
| 43 | first inflorescence length | QN | cm | (Shi et al., 2012) |
| 44 | flesh thickness | QN | cm | (Ministry of Agriculture of China, 2013) |
| 45 | fruit area |  | cm2 | (Brewer et al., 2006) |
| 46 | Fruit brush length | QN | cm |  |
| 47 | Fruit color | QL | yellow | (Ministry of Agriculture of China, 2013) |
|  |  |  | red |  |
|  |  |  | black |  |
| 48 | Fruit color | QN |  | (Ali et al., 2010) |
| 49 | fruit external color (abL value) |  | average 'a' value | SGN(Fernandez-Pozo et al., 2014) & (Weingerl and Unuk, 2015) |
|  |  |  | average 'b' value |  |
|  |  |  | average 'L' value |  |
| 50 | fruit external colour (RGB value) | QN | average RGB blue | SGN(Fernandez-Pozo et al., 2014) &(Gautier et al., 2005) |
|  |  |  | average RGB green |  |
|  |  |  | average RGB red |  |
| 51 | fruit firmness | QN | N | (Hernández-Pérez et al., 2020) |
| 52 | fruit glossiness | QN | dull epidermis | (Frary et al., 2003) |
|  |  |  | intermediate glossy |  |
|  |  |  | glossy epidermis |  |
| 53 | Fruit length | QN | cm | (Rehman et al., 2020) & (Ministry of Agriculture of China, 2013) |
| 54 | fruit lobedness degree | QN |  | (Maria et al., 2009) |
| 55 | fruit locule number | QN |  | SGN(Fernandez-Pozo et al., 2014) & (Maria et al., 2009) |
| 56 | fruit maximum height | QN | cm | (Mazzucato et al., 2010) |
| 57 | fruit maximum width | QN | cm | (Mazzucato et al., 2010) |
| 58 | fruit number in bud eye | QN |  | (Shi et al., 2012) |
| 59 | fruit placenta color (abL value) | QN | average 'a' value | SGN(Fernandez-Pozo et al., 2014) & (Weingerl and Unuk, 2015) |
|  |  |  | average 'b' value |  |
|  |  |  | average 'L' value |  |
| 60 | fruit placenta color (RGB value) | QN | average RGB blue | SGN(Fernandez-Pozo et al., 2014) &(Gautier et al., 2005) |
|  |  |  | average RGB green |  |
|  |  |  | average RGB red |  |
| 61 | fruit section area | QN | cm2 | (Mazzucato et al., 2010) |
| 62 | fruit setting | QN |  | (Javaria et al., 2012) & (Solaiman and Rabbani, 2006) |
| 63 | Fruit setting rate of self-pollination | QN | % | (Shi et al., 2012) |
| 64 | Fruit shape | PQ | Long elliptical | (Ministry of Agriculture of China, 2013) & (Mazzucato et al., 2010) |
|  |  |  | Moderately elliptic |  |
|  |  |  | globular |  |
|  |  |  | Flat spherical |  |
|  |  |  | Globose Obovoid |  |
| 65 | Fruit shape index | QN |  | (Rehman et al., 2020) & (Qi et al., 2019) &(Mazzucato et al., 2010) |
| 66 | Fruit shape index internal | QN |  | (Nankar et al., 2020) |
| 67 | fruit shape triangle | QN |  | (Mazzucato et al., 2010) &(Nankar et al., 2020) |
| 68 | fruit shape triangle 10% | QN |  | (Gonzalo and van der Knaap, 2008) |
| 69 | fruit shape triangle 20% | QN |  | (Gonzalo and van der Knaap, 2008) |
| 70 | fruit shape triangle 30% | QN |  | (Gonzalo and van der Knaap, 2008) |
| 71 | fruit shape triangle 5% | QN |  | (Gonzalo and van der Knaap, 2008) |
| 72 | fruit stalk length | QN | cm | (Ministry of Agriculture of China, 2013) |
| 73 | Fruit weight | QN | g | (Rehman et al., 2020) &(Ministry of Agriculture of China, 2013) |
| 74 | Fruit width | QN | cm | (Rehman et al., 2020) &(Ministry of Agriculture of China, 2013) |
| 75 | fruit yield per plant | QN | kg | (Shi et al., 2012) |
| 76 | heart shape | QN |  | (Gonzalo and van der Knaap, 2008) |
| 77 | Height mid-width | QN | mm | (Nankar et al., 2020) |
| 78 | Hydrolyzed Sugars | QN | % | (Yao et al., 2018a) |
| 79 | internal color uniformity cross section | QN | percent red % | SGN(Fernandez-Pozo et al., 2014) & (Gharezi and Gharezi, 2012) |
|  |  |  | percent yellow % | SGN(Fernandez-Pozo et al., 2014) & (Gharezi and Gharezi, 2012) |
| 80 | internal color uniformity longitudinal section | QN | percent red % | SGN(Fernandez-Pozo et al., 2014) &(Gharezi and Gharezi, 2012) |
|  |  |  | percent yellow % |  |
| 81 | internal eccentricity | QN |  | (Maria et al., 2009) |
| 82 | Lobedness Degree | QN |  | (Nankar et al., 2020) |
| 83 | locule number | QN |  | SGN(Fernandez-Pozo et al., 2014) |
| 84 | mesocarp cross section color | QN | chroma | (Mendes et al., 2020) |
| 85 | mesocarp cross section color | QN | hue | (Carrillo-López and Yahia, 2014) |
| 86 | mesocarp cross section color | QN | luminosity | (Constantino et al., 2021) |
| 87 | mesocarp cross section color （abL value） | QN | average 'a' value | (Carrillo-López and Yahia, 2014) |
|  |  |  | average 'b' value |  |
|  |  |  | average 'L' value |  |
| 88 | mesocarp cross section color （RGB value） | QN | average RGB blue | (Li et al., 2015) |
|  |  |  | average RGB green |  |
|  |  |  | average RGB red |  |
| 89 | mesocarp cross section color uniformity | QN | percent red % | (Rodríguez et al., 2010) &(Pestorić et al., 2021) |
|  |  |  | percent yellow % |  |
| 90 | number of fruits per end cluster | QN | Fruits/end cluster | (Rehman et al., 2020) |
| 91 | Number of fruits per nod | QN | Fruits/nod | (Rehman et al., 2020) |
| 92 | Number of seed per fruit | QN | seeds/fruit | (Ministry of Agriculture of China, 2013) |
| 93 | percentage of drop | QN | % | (Shi et al., 2012) |
| 94 | pericarp area | QN | cm2 | (Maria et al., 2009) |
| 95 | Pericarp thickness | QN | cm | (Li et al., 2018) |
| 96 | Perimeter | QN | mm | (Nankar et al., 2020) |
| 97 | placenta area | QN | mm | (Maria et al., 2009) |
| 98 | placenta color chroma | QN |  | SGN(Fernandez-Pozo et al., 2014) &(Rodríguez et al., 2010) & (Li et al., 2015) |
| 99 | placenta color hue | QN |  | SGN(Fernandez-Pozo et al., 2014) &(Rodríguez et al., 2010) &(Li et al., 2015) |
| 100 | placenta color luminosity | QN |  | SGN(Fernandez-Pozo et al., 2014) &(Rodríguez et al., 2010) &(Li et al., 2015) |
| 101 | placenta color uniformity | QN | percent red % | (Brewer et al., 2008) |
|  |  |  | percent yellow % |  |
| 102 | proximal angle macro 20% | QN |  | (Rodríguez et al., 2010) |
| 103 | proximal angle micro 2% | QN |  | (Rodríguez et al., 2010) |
| 104 | Proximal eccentricity | QN |  | (Nankar et al., 2020) |
| 105 | proximal eccentricity index | QN |  | (Panthee et al., 2013) |
| 106 | Proximal fruit blockiness | QN |  | (Nankar et al., 2020) |
| 107 | proximal fruit color chroma | QN |  | (Mendes et al., 2020) |
| 108 | proximal fruit color hue | QN |  | (Carrillo-López and Yahia, 2014) |
| 109 | proximal fruit color luminosity | QN |  | (Constantino et al., 2021) |
| 110 | proximal fruit color uniformity | QN | percent red % | (Brewer et al., 2008) |
|  |  |  | percent yellow % |  |
| 111 | proximal fruit end blockiness 10% | QN |  | (Rodríguez et al., 2010) |
| 112 | proximal fruit end blockiness 20% | QN |  | (Rodríguez et al., 2010) |
| 113 | proximal fruit end blockiness 30% | QN |  | (Rodríguez et al., 2010) |
| 114 | proximal fruit end blockiness 5% | QN |  | (Rodríguez et al., 2010) |
| 115 | proximal fruit end color | QN |  |  |
| 116 | proximal fruit end color(abL value) | QN | average 'a' value | SGN(Fernandez-Pozo et al., 2014)&(Rodríguez et al., 2010) |
|  |  |  | average 'b' value |  |
|  |  |  | average 'L' value |  |
| 117 | proximal fruit end color (RGB value) | QN | average RGB blue | SGN(Fernandez-Pozo et al., 2014) & (Rodríguez et al., 2010) |
|  |  |  | average RGB green |  |
|  |  |  | average RGB red |  |
| 118 | proximal fruit end height | QN |  | (Maria et al., 2009) |
| 119 | proximal fruit end blockiness shape | QL | very sharp |  |
|  |  |  | sharp |  |
|  |  |  | elliptical |  |
|  |  |  | dull |  |
|  |  |  | sunken |  |
| 120 | Rectangular |  |  | (Nankar et al., 2020) |
| 121 | Sensory evaluation | QN | Odor | (Belović et al., 2012) |
|  |  |  | Off-odor |  |
|  |  |  | Sour taste |  |
|  |  |  | Sweet taste |  |
|  |  |  | Off-taste |  |
|  |  |  | Flavor |  |
|  |  |  | After taste |  |
| 122 | septum area | QN |  | (Maria et al., 2009) |
| 123 | shape index internal | QN |  | (Maria et al., 2009) |
| 124 | simultaneity of fruit ripening | QN | [asynchronous](file:///C:\Users\GHG\AppData\Local\youdao\dict\Application\8.9.9.0\resultui\html\index.html#javascript:;) | (Shi et al., 2012) |
|  |  |  | intermediate |  |
|  |  |  | simultaneous |  |
| 125 | Single fruit weight | QN | g | (Qi et al., 2019) |
| 126 | Texture in mouth | QN | Skin chewiness | (Belović et al., 2012) |
|  |  |  | Firmness |  |
|  |  |  | Solubility |  |
|  |  |  | Juiciness |  |
|  |  |  | Chewiness |  |
|  |  |  | Mealiness |  |
|  |  |  | Covering |  |
| 127 | transect diameter of the fruits | QN | mm | (Qi et al., 2019) |
| 128 | vertical diameter of the fruits | QN | mm | (Qi et al., 2019) |
| 129 | Width mid-height | QN | mm | (Nankar et al., 2020) |

Abbreviation: QN, quantitative characteristics; QL, qualitative characteristics; PQ, pseudo-qualitative characteristics.

**Table S6. leaf related traits**

|  | **Traits** | **Character** | **Description** | | **Reference** | |
| --- | --- | --- | --- | --- | --- | --- |
| 1 | Chlorophyll content | QN |  | | (Wang et al., 2020) | |
| 2 | condition of leaf surface | QL | positive winding | | (Shi et al., 2012) | |
|  |  |  | reverse winding | |  | |
|  |  |  | exlanate | |  | |
| 3 | diameter of subcylindric leaves | QN | mm | | (Qi et al., 2019) | |
| 4 | gloss of leaf surface | QL | with | | (Shi et al., 2012) | |
|  |  |  | without | |  | |
| 5 | Green degree of leaf | QL | light | | (Ministry of Agriculture of China, 2013) | |
|  |  |  | intermediate | |  | |
|  |  |  | dark | |  | |
| 6 | intercellular CO2 concentration | QN | μmol·mol-1 | | (Gong et al., 2019) | |
| 7 | leaf color | QL | brown green | | SGN(Fernandez-Pozo et al., 2014) | |
|  |  |  | dark green | |  | |
|  |  |  | dull green/grey | |  | |
|  |  |  | variegation | |  | |
|  |  |  | purple | |  | |
|  |  |  | white | |  | |
|  |  |  | yellow | |  | |
|  |  |  | virescent | |  | |
| 8 | leaf length | QN | cm | | (Ministry of Agriculture of China, 2013) & (Ministry of Agriculture of China, 2013) & (Wei et al., 2008) | |
| 9 | Leaf lobing | PQ | very weak lobing | | (Frary et al., 2003) | |
|  |  |  | weak lobing | |  | |
|  |  |  | intermediate | |  | |
|  |  |  | strong lobing | |  | |
|  |  |  | very strong lobing | |  | |
| 10 | leaf shape | PQ | Strip shape | | (Ministry of Agriculture of China, 2013) | |
|  |  |  | strip Lanceolate | |  | |
|  |  |  | Wide lanceolate | |  | |
|  |  |  | elliptic-lanceolate | |  | |
|  |  |  | ovate | |  | |
| 11 | Leaf shape index | QN |  | | (Rehman et al., 2020) &(Frary et al., 2003) | |
| 12 | Leaf shape index of oblanceolate leaves | QN |  | | (Qi et al., 2019) | |
| 13 | leaf stalk length | QN | cm | | (Wei et al., 2008) | |
| 14 | Leaf surface appearance | PQ | smooth | | (Frary et al., 2003) | |
|  |  |  | intermediate rugose | |  | |
|  |  |  | rugose | |  | |
|  |  |  | intermediate wrinkled | |  | |
|  |  |  | strongly wrinkled | |  | |
| 15 | leaf thickness | QN | mm | (Shi et al., 2012) & (Wei et al., 2008) | |  |
| 16 | leaf width | QN | cm | | (Ministry of Agriculture of China, 2013) &(Wei et al., 2008) & (Frary et al., 2003) | |
| 17 | length of oblanceolate leaves | QN | cm | | (Qi et al., 2019) | |
| 18 | length of subcylindric leaves | QN | cm | | (Qi et al., 2019) | |
| 19 | limiting value of the stoma | QN |  | | (Gong et al., 2019) | |
| 20 | Net photosynthetic rate | QN | CO2μmol·m-2·S-1 | | (Gong et al., 2019) | |
| 21 | number of leaf clusters within 10 cm of the bearing shoots | QN |  | | (Qi et al., 2019) | |
| 22 | number of leaves to 1st inflorescence | QN |  | | SGN(Fernandez-Pozo et al., 2014) | |
| 23 | petiole length | QN | cm | | (Ministry of Agriculture of China, 2013) | |
| 24 | phyllotaxy | QL | opposite | | (Shi et al., 2012) | |
|  |  |  | alternate | |  | |
| 25 | shape of leaf apex | QL | acutus | | (Shi et al., 2012) | |
|  |  |  | Taper | |  | |
|  |  |  | Blunt round | |  | |
| 26 | Stomatal area | QN | μm2 | | (Wang et al., 2020) | |
| 27 | stomatal conductance | QN | H2Omol .m-2.s-1 | | (Gong et al., 2019) | |
| 28 | Stomatal density | QN | stomatal number/mm2 | | (Wang et al., 2020) | |
| 29 | Stomatal length | QN | μm | | (Wang et al., 2020) | |
| 30 | Stomatal width | QN | μm | | (Wang et al., 2020) | |
| 31 | transpiration rate | QN | H2Ommol·m-2·s-1 | | (Gong et al., 2019) | |
| 32 | water use efficiency | QN | H2Ommol·m-2·s-1 | | (Gong et al., 2019) | |
| 33 | width of oblanceolate leaves | QN | mm | | (Qi et al., 2019) | |

Abbreviation: QN, quantitative characteristics; QL, qualitative characteristics; PQ, pseudo-qualitative characteristics.

**Table S7. Flower related traits**

|  | **Traits** | **Character** | **Description** | **Reference** |
| --- | --- | --- | --- | --- |
| 1 | color of corolla | QL | white | (Ministry of Agriculture of China, 2013) |
|  |  |  | violet |  |
|  |  |  | purple |  |
| 2 | corolla shape | QL | cannular | (Shi et al., 2012) |
|  |  |  | infundibuliform |  |
| 3 | echinoid density in corolla lobe | QN | none | (State Forestry Administration of China, 2013) |
|  |  |  | rare |  |
|  |  |  | thick |  |
| 4 | female sterile | QL | full sterility | SGN(Fernandez-Pozo et al., 2014) |
|  |  |  | partial sterility |  |
|  |  |  | fertility |  |
| 5 | first inflorescence length | QN | cm | (Wei et al., 2008) |
| 6 | flower diameter | QN | cm | (Wei et al., 2008)&(Frary et al., 2003) |
| 7 | flower number per nod | QN | flower/nod | Same with number of fruits per node in (Rehman et al., 2020) |
| 8 | flower terminal | QL | Yes |  |
|  |  |  | No |  |
| 9 | flower terminal rate | QN | % | Percentage of flower terminal branches in a plant |
| 10 | inflorescence | QL | solitary | (Shi et al., 2012) |
|  |  |  | fascicle |  |
| 11 | length of corolla | QN | cm | (Shi et al., 2012) |
| 12 | length of stigma exertion |  | cm | (Chen and Tanksley, 2004) |
| 13 | male sterile | QL | full sterility | SGN(Fernandez-Pozo et al., 2014) |
|  |  |  | partial sterility |  |
|  |  |  | fertility |  |
| 14 | number of flowers | QL |  | (Nurullayeva et al., 2021) |
| 15 | ovary length | QL | cm | (Chen and Tanksley, 2004) |
| 16 | petal diameter | QN | cm | (Ministry of Agriculture of China, 2013) |
| 17 | petal front shape | PQ | sharp | (Ministry of Agriculture of China, 2013) |
|  |  |  | medium sharp |  |
|  |  |  | globular |  |
| 18 | petal number per flower | QN | number/flower |  |
| 19 | pistil length | QN | cm | (Chen and Tanksley, 2004) |
| 20 | Pollen Vitality | QN | % | (Schubert et al., 2019) |
| 21 | sepal length | QN | cm | (State Forestry Administration of China, 2013) |
| 22 | sepal number per flower | QN |  | (State Forestry Administration of China, 2013) |
| 23 | Sepal shape | PQ | triangular | (State Forestry Administration of China, 2013) |
|  |  |  | ovate |  |
|  |  |  | Broadly ovate, |  |
| 24 | stamen length | QN | cm | (Chen and Tanksley, 2004) |
| 25 | style length | QN | cm | (Chen and Tanksley, 2004) |
| 26 | The length of peduncles | QN | mm | (Qi et al., 2019) |
| 27 | Water Content of Stamen | QN | % | (Schubert et al., 2019) |

Abbreviation: QN, quantitative characteristics; QL, qualitative characteristics; PQ, pseudo-qualitative characteristics.

**Table S8 Candidate trait for seed related trait QTL**

|  | **Trait** | **Character** | **Description** | **reference** |
| --- | --- | --- | --- | --- |
| 1 | 100 seed weight | QL | g | (Rehman et al., 2020) |
| 2 | 1000 seed weight |  | g | (Shi et al., 2012) |
| 3 | full abortive rate of seed | QN | % | (Shi et al., 2012) |
| 4 | number of seed per fruit | QN | number/fruit | (Rehman et al., 2020) |
| 5 | seed colour | QL | yellow | (Shi et al., 2012) |
|  |  |  | light yellow |  |
|  |  |  | Brown yellow |  |
| 6 | seed germination |  | % | (Nurullayeva et al., 2021) |
| 7 | seed shape | QL | kidney-shaped | (Shi et al., 2012) |
|  |  |  | rounded |  |
|  |  |  | ovate |  |
| 8 | seed vigour index | QN |  | (Sharma, 2012) |

Abbreviation: QN, quantitative characteristics; QL, qualitative characteristics; PQ, pseudo-qualitative characteristics.

**Table S9. Candidate traits for metabolic QTL**

|  | **Classification** | **Traits** | **organ** | **property** | | **Description** | **reference** |
| --- | --- | --- | --- | --- | --- | --- | --- |
| 1 | Biogenic amines | cadaverine | bark | AC | mg/kg | | (Ai et al., 2021) |
| 2 | Biogenic amines | histamine | bark | AC | mg/kg | | (Ai et al., 2021) |
| 3 | Biogenic amines | methylamine | bark | AC | mg/kg | | (Ai et al., 2021) |
| 4 | Biogenic amines | phenyl ethylamine | bark | AC | mg/kg | | (Ai et al., 2021) |
| 5 | Biogenic amines | putrescine | bark | AC | mg/kg | | (Ai et al., 2021) |
| 6 | Biogenic amines | spermidine | bark | AC | mg/kg | | (Ai et al., 2021) |
| 7 | Biogenic amines | spermine | bark | AC | mg/kg | | (Ai et al., 2021) |
| 8 | Biogenic amines | tryptamine | bark | AC | mg/kg | | (Ai et al., 2021) |
| 9 | Biogenic amines | tyramine | bark | AC | mg/kg | | (Ai et al., 2021) |
| 10 | Anthraquinone | Kukoamines A | dry root | AC | mg/g | | (Li et al., 2017) |
| 11 | Anthraquinone | Kukoamines B | dry root | AC | mg/g | | (Li et al., 2017) |
| 12 | Biogenic amines | cadaverine | Flower | AC | mg/kg | | (Ai et al., 2021) |
| 13 | Biogenic amines | histamine | Flower | AC | mg/kg | | (Ai et al., 2021) |
| 14 | Biogenic amines | methylamine | Flower | AC | mg/kg | | (Ai et al., 2021) |
| 15 | Biogenic amines | phenyl ethylamine | Flower | AC | mg/kg | | (Ai et al., 2021) |
| 16 | Biogenic amines | putrescine | Flower | AC | mg/kg | | (Ai et al., 2021) |
| 17 | Biogenic amines | spermidine | Flower | AC | mg/kg | | (Ai et al., 2021) |
| 18 | Biogenic amines | spermine | Flower | AC | mg/kg | | (Ai et al., 2021) |
| 19 | Biogenic amines | tryptamine | Flower | AC | mg/kg | | (Ai et al., 2021) |
| 20 | Biogenic amines | tyramine | Flower | AC | mg/kg | | (Ai et al., 2021) |
| 21 | flavonoids | kaempferol | flower | AC | μg/g | | (Zhao et al., 2013) |
| 22 | flavonoids | rutin | flower | AC | μg/g | | (Zhao et al., 2013) |
| 23 | Phenylpropanoids | caffeic acid | flower | AC | μg/g | | (Zhao et al., 2013) |
| 24 | Phenylpropanoids | chlorogeic acid | flower | AC | μg/g | | (Zhao et al., 2013) |
| 25 | Phenylpropanoids | ferulic acid | flower | AC | μg/g | | (Zhao et al., 2013) |
| 26 | Phenylpropanoids | trans-cinnamic acid | flower | AC | μg/g | | (Zhao et al., 2013) |
| 27 | amino acids | Alanine | fruit | AC | mg/kg | | (Yu et al., 2017) &(Guo et al., 2015) |
| 28 | amino acids | Arginine | fruit | AC | mg/kg | | (Yu et al., 2017) |
| 29 | amino acids | asparagine | fruit | AC | mg/kg | | (Guo et al., 2015) |
| 30 | amino acids | Aspartic acid | fruit | AC | mg/kg | | (Yu et al., 2017) &(Guo et al., 2015) |
| 31 | amino acids | Cysteine | fruit | AC | mg/kg | | (Yu et al., 2017) &(Guo et al., 2015) |
| 32 | amino acids | Glutamine | fruit | AC | mg/kg | | (Yu et al., 2017) & (Guo et al., 2015) |
| 33 | amino acids | Glycine | fruit | AC | mg/kg | | (Yu et al., 2017) & (Guo et al., 2015) |
| 34 | amino acids | Histidine | fruit | AC | mg/kg | | (Yu et al., 2017) &(Guo et al., 2015) |
| 35 | amino acids | Isoleucine | fruit | AC | mg/kg | | (Yu et al., 2017) &(Guo et al., 2015) |
| 36 | amino acids | Leucine | fruit | AC | mg/kg | | (Yu et al., 2017) &(Guo et al., 2015) |
| 37 | amino acids | Lysine | fruit | AC | mg/kg | | (Yu et al., 2017) &(Guo et al., 2015) |
| 38 | amino acids | Methionine | fruit | AC | mg/kg | | (Yu et al., 2017) &(Guo et al., 2015) |
| 39 | amino acids | Phenylalanine | fruit | AC | mg/kg | | (Yu et al., 2017) &(Guo et al., 2015) |
| 40 | amino acids | Proline | fruit | AC | mg/kg | | (Yu et al., 2017) &(Guo et al., 2015) |
| 41 | amino acids | Serine | fruit | AC | mg/kg | | (Yu et al., 2017) &(Guo et al., 2015) |
| 42 | amino acids | Threonine | fruit | AC | mg/kg | | (Yu et al., 2017) &(Guo et al., 2015) |
| 43 | amino acids | tryptophan | fruit | AC | mg/kg | | (Guo et al., 2015) |
| 44 | amino acids | Tyrosine | fruit | AC | mg/kg | | (Yu et al., 2017) &(Guo et al., 2015) |
| 45 | amino acids | Valine | fruit | AC | mg/kg | | (Yu et al., 2017) &(Guo et al., 2015) |
| 46 | amono acids | glutamic acid | fruit | AC |  | | SGN (Wu et al., 2016) |
| 47 | Biogenic amines | cadaverine | fruit | AC | mg/kg | | (Ai et al., 2021) |
| 48 | Biogenic amines | histamine | fruit | AC | mg/kg | | (Ai et al., 2021) |
| 49 | Biogenic amines | methylamine | fruit | AC | mg/kg | | (Ai et al., 2021) |
| 50 | Biogenic amines | phenyl ethylamine | fruit | AC | mg/kg | | (Ai et al., 2021) |
| 51 | Biogenic amines | putrescine | fruit | AC | mg/kg | | (Ai et al., 2021) |
| 52 | Biogenic amines | spermidine | fruit | AC | mg/kg | | (Ai et al., 2021) |
| 53 | Biogenic amines | spermine | fruit | AC | mg/kg | | (Ai et al., 2021) |
| 54 | Biogenic amines | tryptamine | fruit | AC | mg/kg | | (Ai et al., 2021) |
| 55 | Biogenic amines | tyramine | fruit | AC | mg/kg | | (Ai et al., 2021) |
| 56 | Carotenoid | 13- or 13’-cis-b-carotene | fruit | AC | mg/g | | (Wang et al., 2010) |
| 57 | Carotenoid | 13- or 13’-cis-zeaxanthin | fruit | AC | mg/g | | (Wang et al., 2010) |
| 58 | Carotenoid | 15- or 15’–cis-zeaxanthin | fruit | AC | mg/g | | (Wang et al., 2010) |
| 59 | Carotenoid | 9- or 9’-cis-b-carotene | fruit | AC | mg/g | | (Wang et al., 2010) |
| 60 | Carotenoid | 9- or 9’-cis-b-cryptoxanthin | fruit | AC | mg/g | | (Wang et al., 2010) |
| 61 | Carotenoid | All-trans-b-carotene | fruit | AC | mg/g | | (Wang et al., 2010) |
| 62 | Carotenoid | All-trans-b-cryptoxanthin | fruit | AC | mg/g | | (Wang et al., 2010) |
| 63 | Carotenoid | All-trans-zeaxanthin | fruit | AC | mg/g | | (Wang et al., 2010) |
| 64 | Carotenoid | Neoxanthin | fruit | AC | mg/g | | (Wang et al., 2010) |
| 65 | Carotenoid | Total carotenoid | fruit | AC | % | | (Peng et al., 2005) |
| 66 | Carotenoid | Zeaxanthin dipalmitate | fruit | AC | % | | (Peng et al., 2005) |
| 67 | Carotenoid | Zeaxanthin fraction 9- or 9’-cis-zeaxanthin | fruit | AC | mg/g | | (Wang et al., 2010) |
| 68 | carotenoids | β-carotene | fruit | AC | μg/100g | | (Oguz and Erdogan, 2016) |
| 69 | Fatty acids | Antioxidant activity | fruit | AC | % | | (Guo et al., 2015) |
| 70 | Fatty acids | Arachidic | fruit | AC | % | | (Guo et al., 2015) |
| 71 | Fatty acids | Fibre | fruit | AC | % | | (Guo et al., 2015) |
| 72 | Fatty acids | Linoleic | fruit | AC | % | | (Guo et al., 2015) |
| 73 | Fatty acids | Linolenic | fruit | AC | % | | (Guo et al., 2015) |
| 74 | Fatty acids | Myristic | fruit | AC | % | | (Guo et al., 2015) |
| 75 | Fatty acids | Oleic | fruit | AC | % | | (Guo et al., 2015) |
| 76 | Fatty acids | Palmitic | fruit | AC | % | | (Guo et al., 2015) |
| 77 | Fatty acids | Stearic | fruit | AC | % | | (Guo et al., 2015) |
| 78 | Fatty acids | Total phenol | fruit | AC | % | | (Guo et al., 2015) |
| 79 | Flavonoid | Acidic fraction Chlorogenic acid | fruit | AC | mg/g | | (Wang et al., 2010) |
| 80 | Flavonoid | Caffeoylquinic acid | fruit | AC | mg/g | | (Wang et al., 2010) |
| 81 | Flavonoid | Kaempferol-3-O-rutinoside | fruit | AC | mg/g | | (Wang et al., 2010) |
| 82 | Flavonoid | Neutral fraction Quercetin-diglycoside | fruit | AC | mg/g | | (Wang et al., 2010) |
| 83 | Flavonoid | p-Coumaric acid | fruit | AC | mg/g | | (Wang et al., 2010) |
| 84 | Flavonoid | Rutin | fruit | AC | mg/g | | (Wang et al., 2010) |
| 85 | Flavonoids | apigenin | fruit | AC | mg/g | | (Ali et al., 2019) |
| 86 | flavonoids | flavonoid | fruit | AC | g/100g | | NY/T 2741-2015 |
| 87 | flavonoids | Flavonoid analysis | fruit | AC |  | | (Oguz and Erdogan, 2016) |
| 88 | flavonoids | kaempferol | fruit | AC | μg/g | | (Zhao et al., 2013) |
| 89 | Flavonoids | luteolin | fruit | AC | mg/g | | (Ali et al., 2019) |
| 90 | flavonoids | morin | fruit | AC | mg/g | | (Ali et al., 2019) |
| 91 | flavonoids | myricetin | fruit | AC | mg/g | | (Ali et al., 2019) |
| 92 | Flavonoids | naringenin | fruit | AC | mg/kg | | (Zhang et al., 2021) |
| 93 | flavonoids | quercitrin | fruit | AC | mg/g | | (Ali et al., 2019) |
| 94 | Flavonoids | rutin | fruit | AC | μg/g | | (Ali et al., 2019) &(Zhao et al., 2013) &(Zhang et al., 2021) |
| 95 | flavonoids | Total anthocyanin contents | Fruit | AC | mg/100g | | (Wu et al., 2016) |
| 96 | flavonoids | total flavonoids | fruit | AC | μg/100g | | GB 5009.83-2016 |
| 97 | flavonols | hyperoside | fruit | AC | mg/g | | (Ali et al., 2019) |
| 98 | lactones | P-coumaric acid | fruit | AC | mg/g | | (Ali et al., 2019) |
| 99 | oils | Raw oil analysis | fruit | AC | % | | (Endes et al., 2015) |
| 100 | organic acids | malic acid | fruit | AC | mg/100g | | SGN (Oguz and Erdogan, 2016) |
| 101 | organic acids | titratable acid | fruit | AC | % | | SGN (Song et al., 2018) |
| 102 | organic acids | Total organic acids | fruit | AC | mg/g | | SGN (Zhao et al., 2015) |
| 103 | Phenylpropanoids | Caffeic acid | fruit | AC | mg/g | | (Zhao et al., 2013) &(Wang et al., 2010) |
| 104 | Phenylpropanoids | chlorogeic acid | fruit | AC | μg/g | | (Zhao et al., 2013) |
| 105 | phenylpropanoids | chlorogenic acid | fruit | AC | mg/kg | | (Zhang et al., 2021) & (Ali et al., 2019) |
| 106 | Phenylpropanoids | ferulic acid | fruit | AC | μg/g | | (Zhang et al., 2021) &(Ali et al., 2019) &(Zhao et al., 2013) |
| 107 | phenylpropanoids | p-coumaric acid | fruit | AC | mg/kg | | (Zhang et al., 2021) |
| 108 | phenylpropanoids | protocatechuic acid | fruit | AC | mg/kg | | (Zhang et al., 2021) |
| 109 | phenylpropanoids | sinapic acid | fruit | AC | mg/kg | | (Zhang et al., 2021) |
| 110 | Phenylpropanoids | trans-cinnamic acid | fruit | AC | μg/g | | (Zhao et al., 2013) |
| 111 | polyamines | spermidine | fruit | AC | g/100g | | NY/T 2741-2015 |
| 112 | Polyphenols | Total Phenolic Content | Fruit | AC | mg/100g | | (Wu et al., 2016) |
| 113 | Polysaccharide | Acidic polysaccharides with different molecular weights. | fruit | AC | mg/g | | (Wang et al., 2010) |
| 114 | Polysaccharide | Crude extract of polysaccharide | fruit | AC | mg/g | | (Wang et al., 2010) |
| 115 | Polysaccharide | Crude polysaccharide | fruit | AC | mg/g | | (Wang et al., 2010) |
| 116 | Polysaccharide | Neutral polysaccharide | fruit | AC | mg/g | | (Wang et al., 2010) |
| 117 | proteins | Crude protein | fruit | AC | % | | (Endes et al., 2015) |
| 118 | proteins | protein content | fruit | AC | g/100g | | (Yao et al., 2018b) &(Oguz and Erdogan, 2016) |
| 119 | quaternary ammonium hydroxide | Betaine | fruit | AC | g/100g | | GB/T5009.4-2003 |
| 120 | saccharides | fructose | fruit | AC | mg/g | | (Ma et al., 2021) |
| 121 | saccharides | glucose content | fruit | AC | mg/g | | SGN (Song et al., 2018) &(Ma et al., 2021) |
| 122 | saccharides | *Lycium barbarum* Polysaccharide | fruit | AC | g/100g | | (He et al., 2012) |
| 123 | saccharides | Pectin content | fruit | AC |  | | (Oguz and Erdogan, 2016) |
| 124 | saccharides | starch | fruit | AC | mg/g | | (Ma et al., 2021) |
| 125 | saccharides | sucrose | fruit | AC | mg/g | | SGN (Ma et al., 2021) |
| 126 | saccharides | total sugar | fruit | AC | g/100g | | (Endes et al., 2015) |
| 127 | Vitamins | vitamin C | fruit | AC | g/100g | | (Oguz and Erdogan, 2016) |
| 128 | Phenolic | Total Phenolic compounds | fruit | AC |  | | (Oguz and Erdogan, 2016) |
| 129 | Aminoglycosides | jasmonoyl‐isoleucine content | in stamens and pistils | AC |  | | (Pan et al., 2019) |
| 130 | hormones | endogenous IAA content | in stamens and pistils | AC |  | | (Pan et al., 2019) |
| 131 | saccharides | fructose content | in stamens and pistils | AC |  | | (Pan et al., 2019) |
| 132 | saccharides | glucose content | in stamens and pistils | AC |  | | (Pan et al., 2019) |
| 133 | saccharides | jasmonate content | in stamens and pistils | AC |  | | (Pan et al., 2019) |
| 134 | saccharides | soluble sucrose content | in stamens and pistils | AC |  | | (Pan et al., 2019) |
| 135 | Biogenic amines | cadaverine | Leaf | AC | mg/kg | | (Ai et al., 2021) |
| 136 | Biogenic amines | histamine | Leaf | AC | mg/kg | | (Ai et al., 2021) |
| 137 | Biogenic amines | methylamine | Leaf | AC | mg/kg | | (Ai et al., 2021) |
| 138 | Biogenic amines | phenylethylamine | Leaf | AC | mg/kg | | (Ai et al., 2021) |
| 139 | Biogenic amines | putrescine | Leaf | AC | mg/kg | | (Ai et al., 2021) |
| 140 | Biogenic amines | spermidine | Leaf | AC | mg/kg | | (Ai et al., 2021) |
| 141 | Biogenic amines | spermine | Leaf | AC | mg/kg | | (Ai et al., 2021) |
| 142 | Biogenic amines | tryptamine | Leaf | AC | mg/kg | | (Ai et al., 2021) |
| 143 | Biogenic amines | tyramine | Leaf | AC | mg/kg | | (Ai et al., 2021) |
| 144 | flavonoids | isoquercitrin | leaf | AC | mg/100g | | (Zhu et al., 2017) |
| 145 | flavonoids | kaempferol | leaf | AC | μg/g | | (Zhu et al., 2017) & (Zhao et al., 2013) |
| 146 | flavonoids | rutin | leaf | AC | μg/g | | (Dong et al., 2009) &(Zhao et al., 2013) |
| 147 | flavonoids | total flavonoids | leaf | AC | mg/g | | (Dong et al., 2009) &(Zhu et al., 2017) |
| 148 | Phenylpropanoids | caffeic acid | leaf | AC | mg/100g | | (Zhao et al., 2013) &(Zhu et al., 2017) |
| 149 | Phenylpropanoids | Chlorogenic acid | leaf | AC | mg/100g | | (Zhao et al., 2013) &(Zhu et al., 2017) |
| 150 | Phenylpropanoids | cryptochlorogenic acid | leaf | AC | mg/100g | | (Zhu et al., 2017) |
| 151 | Phenylpropanoids | ferulic acid | leaf | AC | μg/g | | (Zhao et al., 2013) |
| 152 | Phenylpropanoids | isochlorogenic acid A | leaf | AC | mg/100g | | (Zhu et al., 2017) |
| 153 | Phenylpropanoids | isochlorogenic acid B | leaf | AC | mg/100g | | (Zhu et al., 2017) |
| 154 | Phenylpropanoids | isochlorogenic acid C | leaf | AC | mg/100g | | (Zhu et al., 2017) |
| 155 | Phenylpropanoids | neochlorogenic acid | leaf | AC | mg/100g | | (Zhu et al., 2017) |
| 156 | Phenylpropanoids | trans-cinnamic acid | leaf | AC | μg/g | | (Zhao et al., 2013) |
| 157 | Polyphenols | total polyphenols | leaf | AC | mg/g | | (Zhu et al., 2017) |
| 158 | saccharides | fructose | leaf | AC | mg/g | | (Ma et al., 2021) |
| 159 | saccharides | glucose content | leaf | AC | mg/g | | SGN (Ma et al., 2021) |
| 160 | saccharides | starch | leaf | AC | mg/g | | (Ma et al., 2021) |
| 161 | saccharides | sucrose | leaf | AC | mg/g | | SGN (Ma et al., 2021) |
| 162 | Biogenic amines | cadaverine | root | AC | mg/kg | | (Ai et al., 2021) |
| 163 | Biogenic amines | histamine | root | AC | mg/kg | | (Ai et al., 2021) |
| 164 | Biogenic amines | methylamine | root | AC | mg/kg | | (Ai et al., 2021) |
| 165 | Biogenic amines | phenylethylamine | root | AC | mg/kg | | (Ai et al., 2021) |
| 166 | Biogenic amines | putrescine | root | AC | mg/kg | | (Ai et al., 2021) |
| 167 | Biogenic amines | spermidine | root | AC | mg/kg | | (Ai et al., 2021) |
| 168 | Biogenic amines | spermine | root | AC | mg/kg | | (Ai et al., 2021) |
| 169 | Biogenic amines | tryptamine | root | AC | mg/kg | | (Ai et al., 2021) |
| 170 | Biogenic amines | tyramine | root | AC | mg/kg | | (Ai et al., 2021) |
| 171 | flavonoids | kaempferol | root | AC | μg/g | | (Zhao et al., 2013) |
| 172 | flavonoids | rutin | root | AC | μg/g | | (Zhao et al., 2013) |
| 173 | Phenylpropanoids | caffeic acid | root | AC | μg/g | | (Zhao et al., 2013) |
| 174 | Phenylpropanoids | chlorogeic acid | root | AC | μg/g | | (Zhao et al., 2013) |
| 175 | Phenylpropanoids | ferulic acid | root | AC | μg/g | | (Zhao et al., 2013) |
| 176 | Phenylpropanoids | trans-cinnamic acid | root | AC | μg/g | | (Zhao et al., 2013) |
| 177 | Biogenic amines | cadaverine | stems | AC | mg/kg | | (Ai et al., 2021) |
| 178 | Biogenic amines | histamine | stems | AC | mg/kg | | (Ai et al., 2021) |
| 179 | Biogenic amines | methylamine | stems | AC | mg/kg | | (Ai et al., 2021) |
| 180 | Biogenic amines | phenylethylamine | stems | AC | mg/kg | | (Ai et al., 2021) |
| 181 | Biogenic amines | putrescine | stems | AC | mg/kg | | (Ai et al., 2021) |
| 182 | Biogenic amines | spermidine | stems | AC | mg/kg | | (Ai et al., 2021) |
| 183 | Biogenic amines | spermine | stems | AC | mg/kg | | (Ai et al., 2021) |
| 184 | Biogenic amines | tryptamine | stems | AC | mg/kg | | (Ai et al., 2021) |
| 185 | Biogenic amines | tyramine | stems | AC | mg/kg | | (Ai et al., 2021) |
| 186 | flavonoids | kaempferol | stems | AC | μg/g | | (Zhao et al., 2013) |
| 187 | flavonoids | rutin | stems | AC | μg/g | | (Zhao et al., 2013) |
| 188 | Phenylpropanoids | caffeic acid | stems | AC | μg/g | | (Zhao et al., 2013) |
| 189 | Phenylpropanoids | chlorogeic acid | stems | AC | μg/g | | (Zhao et al., 2013) |
| 190 | Phenylpropanoids | ferulic acid | stems | AC | μg/g | | (Zhao et al., 2013) |
| 191 | Phenylpropanoids | trans-cinnamic acid | stems | AC | μg/g | | (Zhao et al., 2013) |
| 192 | Alcohols and polyols | 1,5-Anhydro-D-glucitol | Fruit | RC |  | | (Shi et al., 2019) |
| 193 | Alcohols and polyols | D-Arabitol | Fruit | RC |  | | (Shi et al., 2019) |
| 194 | Alcohols and polyols | D-Mannitol | Fruit | RC |  | | (Shi et al., 2019) |
| 195 | Alcohols and polyols | D-Sorbitol | Fruit | RC |  | | (Shi et al., 2019) |
| 196 | Alcohols and polyols | Dulcitol | Fruit | RC |  | | (Shi et al., 2019) |
| 197 | Alcohols and polyols | Enterodiol | Fruit | RC |  | | (Shi et al., 2019) |
| 198 | Alcohols and polyols | Pantothenol | Fruit | RC |  | | (Shi et al., 2019) |
| 199 | Alkaloids | Betaine | Fruit | RC |  | | (Shi et al., 2019) |
| 200 | Alkaloids | Camptothecin | Fruit | RC |  | | (Shi et al., 2019) |
| 201 | Alkaloids | Delphinidin-3-O-rutinoside (trans-p-coumaroyl)-5-O-glucoside | Fruit | RC |  | | (Yang et al., 2020) |
| 202 | Alkaloids | Dihydrocaffeoyl caffeoyl spermidine hexoside | Fruit | RC |  | | (Yang et al., 2020) |
| 203 | Alkaloids | Dihydrocaffeoyl caffeoyl spermidine isomer 1 | Fruit | RC |  | | (Yang et al., 2020) |
| 204 | Alkaloids | Dihydrocaffeoyl caffeoyl spermidine isomer 2 | Fruit | RC |  | | (Yang et al., 2020) |
| 205 | Alkaloids | Dihydrocaffeoyl caffeoyl spermidine isomer 3 | Fruit | RC |  | | (Yang et al., 2020) |
| 206 | Alkaloids | Dihydrocaffeoyl caffeoyl spermidine isomer 4 | Fruit | RC |  | | (Yang et al., 2020) |
| 207 | Alkaloids | Dihydrocaffeoyl caffeoyl spermidine isomer 5 | Fruit | RC |  | | (Yang et al., 2020) |
| 208 | Alkaloids | Hordenine | Fruit | RC |  | | (Shi et al., 2019) |
| 209 | Alkaloids | Isohemiphloin | Fruit | RC |  | | (Shi et al., 2019) |
| 210 | Alkaloids | Isoquinoline | Fruit | RC |  | | (Shi et al., 2019) |
| 211 | Alkaloids | Kukoamine A | Fruit | RC |  | | (Yang et al., 2020) |
| 212 | Alkaloids | Malvidin-3-O-rutinoside (trans-p-coumaroyl)-5-O-glucoside | Fruit | RC |  | | (Yang et al., 2020) |
| 213 | Alkaloids | N1,N10-dihydrocaffeoyl spermidine hexoside | Fruit | RC |  | | (Yang et al., 2020) |
| 214 | Alkaloids | N1-Caffeoyl, N10-dihydrocaffeoyl spermidine hexose | Fruit | RC |  | | (Yang et al., 2020) |
| 215 | Alkaloids | N1-Dihydrocaffeoyl, N10-caffeoyl spermidine hexose | Fruit | RC |  | | (Yang et al., 2020) |
| 216 | Alkaloids | N1-Dihydrocaffeoyl, N10-coumaroyl spermidine | Fruit | RC |  | | (Yang et al., 2020) |
| 217 | Alkaloids | N-caffeoyl, N'-dihydrocaffeoyl spermidine dihexose | Fruit | RC |  | | (Yang et al., 2020) |
| 218 | Alkaloids | Petunidin-3-O-rutinoside (cis-p-coumaroyl)-5-O-glucoside | Fruit | RC |  | | (Yang et al., 2020) |
| 219 | Alkaloids | Petunidin-3-O-rutinoside (trans-p-coumaroyl)-5-O-glucoside | Fruit | RC |  | | (Yang et al., 2020) |
| 220 | Alkaloids | Petunidin-3-O-rutinoside (feruloyl)-5-O-glucoside | Fruit | RC |  | | (Yang et al., 2020) |
| 221 | Alkaloids | Petunidin-3-O-rutinoside (glucosyl-cis-p-coumaroyl)-5-O-glucoside | Fruit | RC |  | | (Yang et al., 2020) |
| 222 | Alkaloids | Petunidin-3-O-rutinoside (p-coumaroyl)-5-O-glucoside isomer | Fruit | RC |  | | (Yang et al., 2020) |
| 223 | Alkaloids | Petunidin-3-O-rutinoside (trans-caffeoyl)-5-O-glucoside | Fruit | RC |  | | (Yang et al., 2020) |
| 224 | Alkaloids | Petunidin-3-O-rutinoside(glucosyl-trans-p-coumaroyl)-5-O-glucoside | Fruit | RC |  | | (Yang et al., 2020) |
| 225 | Alkaloids | Petunidin-3-O-rutinoside-5-O-glucoside | Fruit | RC |  | | (Yang et al., 2020) |
| 226 | Alkaloids | Piperidine | Fruit | RC |  | | (Shi et al., 2019) |
| 227 | Alkaloids | Trigonelline | Fruit | RC |  | | (Shi et al., 2019) |
| 228 | Amino acid derivatives | (-)-3-(3,4-Dihydroxyphenyl)-2-methylalanine | Fruit | RC |  | | (Shi et al., 2019) |
| 229 | Amino acid derivatives | (5-L-Glutamyl)-L-amino acid | Fruit | RC |  | | (Shi et al., 2019) |
| 230 | Amino acid derivatives | 1-Methylhistidine | Fruit | RC |  | | (Shi et al., 2019) |
| 231 | Amino acid derivatives | 2,3-dimethylsuccinic acid | Fruit | RC |  | | (Shi et al., 2019) |
| 232 | Amino acid derivatives | 2,6-Diaminooimelic acid | Fruit | RC |  | | (Shi et al., 2019) |
| 233 | Amino acid derivatives | 2-Aminoisobutyric acid | Fruit | RC |  | | (Shi et al., 2019) |
| 234 | Amino acid derivatives | 3-(2-Naphthyl)-D-alanine | Fruit | RC |  | | (Shi et al., 2019) |
| 235 | Amino acid derivatives | 3-(6-Hydroxy-3,4-dioxo-1,5-cyclohexadien-1-yl)-L-alanine | Fruit | RC |  | | (Shi et al., 2019) |
| 236 | Amino acid derivatives | 3,4-Dihydroxy-DL-phenylalanine | Fruit | RC |  | | (Shi et al., 2019) |
| 237 | Amino acid derivatives | 3-Hydroxy-3-methylpentane-1,5-dioic acid | Fruit | RC |  | | (Shi et al., 2019) |
| 238 | Amino acid derivatives | 3-N-Methyl-L-histidine | Fruit | RC |  | | (Shi et al., 2019) |
| 239 | Amino acid derivatives | 4-Hydroxy-L-glutamic acid | Fruit | RC |  | | (Shi et al., 2019) |
| 240 | Amino acid derivatives | 5-Aminovaleric acid | Fruit | RC |  | | (Shi et al., 2019) |
| 241 | Amino acid derivatives | 5-Hydroxy-L-tryptophan | Fruit | RC |  | | (Shi et al., 2019) |
| 242 | Amino acid derivatives | 5-oxoproline | Fruit | RC |  | | (Shi et al., 2019) |
| 243 | Amino acid derivatives | Acetyl tryptophan | Fruit | RC |  | | (Shi et al., 2019) |
| 244 | Amino acid derivatives | Allysine(6-Oxo DL-Norleucine) | Fruit | RC |  | | (Shi et al., 2019) |
| 245 | Amino acid derivatives | Aspartic acid di-O-glucoside | Fruit | RC |  | | (Shi et al., 2019) |
| 246 | Amino acid derivatives | Asp-phe | Fruit | RC |  | | (Shi et al., 2019) |
| 247 | Amino acid derivatives | CYS-GLY | Fruit | RC |  | | (Shi et al., 2019) |
| 248 | Amino acid derivatives | D-Alanyl-D-Alanine | Fruit | RC |  | | (Shi et al., 2019) |
| 249 | Amino acid derivatives | Glutathione oxidized | Fruit | RC |  | | (Shi et al., 2019) |
| 250 | Amino acid derivatives | Glutathione reduced form | Fruit | RC |  | | (Shi et al., 2019) |
| 251 | Amino acid derivatives | H-HomoArg-OH | Fruit | RC |  | | (Shi et al., 2019) |
| 252 | Amino acid derivatives | Histamine | Fruit | RC |  | | (Shi et al., 2019) |
| 253 | Amino acid derivatives | L-Glutamic acid O-glucoside | Fruit | RC |  | | (Shi et al., 2019) |
| 254 | Amino acid derivatives | L-Glutamine O-hexside | Fruit | RC |  | | (Shi et al., 2019) |
| 255 | Amino acid derivatives | L-Glutaminyl-L-valyl-L-valyl-L-cysteine | Fruit | RC |  | | (Shi et al., 2019) |
| 256 | Amino acid derivatives | L-Kynurenine | Fruit | RC |  | | (Shi et al., 2019) |
| 257 | Amino acid derivatives | L-Methionine methyl ester | Fruit | RC |  | | (Shi et al., 2019) |
| 258 | Amino acid derivatives | L-Pipecolic acid | Fruit | RC |  | | (Shi et al., 2019) |
| 259 | Amino acid derivatives | L-Saccharopine | Fruit | RC |  | | (Shi et al., 2019) |
| 260 | Amino acid derivatives | Lysine butyrate | Fruit | RC |  | | (Shi et al., 2019) |
| 261 | Amino acid derivatives | Methionine sulfoxide | Fruit | RC |  | | (Shi et al., 2019) |
| 262 | Amino acid derivatives | N-(3-Indolylacetyl)-L-alanine | Fruit | RC |  | | (Shi et al., 2019) |
| 263 | Amino acid derivatives | N,N-Dimethylglycine | Fruit | RC |  | | (Shi et al., 2019) |
| 264 | Amino acid derivatives | N6-Acetyl-L-lysine | Fruit | RC |  | | (Shi et al., 2019) |
| 265 | Amino acid derivatives | N-Acetylaspartate | Fruit | RC |  | | (Shi et al., 2019) |
| 266 | Amino acid derivatives | N-acetylglycine | Fruit | RC |  | | (Shi et al., 2019) |
| 267 | Amino acid derivatives | N-Acetyl-L-glutamic acid | Fruit | RC |  | | (Shi et al., 2019) |
| 268 | Amino acid derivatives | N-Acetyl-l-leucine | Fruit | RC |  | | (Shi et al., 2019) |
| 269 | Amino acid derivatives | N-Acetyl-L-tyrosine | Fruit | RC |  | | (Shi et al., 2019) |
| 270 | Amino acid derivatives | N-Acetylmethionine | Fruit | RC |  | | (Shi et al., 2019) |
| 271 | Amino acid derivatives | N-Acetylthreonine | Fruit | RC |  | | (Shi et al., 2019) |
| 272 | Amino acid derivatives | N'-Formylkynurenine | Fruit | RC |  | | (Shi et al., 2019) |
| 273 | Amino acid derivatives | N-formylmethionine | Fruit | RC |  | | (Shi et al., 2019) |
| 274 | Amino acid derivatives | N-Glycyl-L-leucine | Fruit | RC |  | | (Shi et al., 2019) |
| 275 | Amino acid derivatives | N-Propionylglycine | Fruit | RC |  | | (Shi et al., 2019) |
| 276 | Amino acid derivatives | Nα-Acetyl-L-arginine | Fruit | RC |  | | (Shi et al., 2019) |
| 277 | Amino acid derivatives | Nα-Acetyl-L-glutamine | Fruit | RC |  | | (Shi et al., 2019) |
| 278 | Amino acid derivatives | N-γ-Acetyl-N-2-Formyl-5-methoxykynurenamine | Fruit | RC |  | | (Shi et al., 2019) |
| 279 | Amino acid derivatives | Phe-Phe | Fruit | RC |  | | (Shi et al., 2019) |
| 280 | Amino acid derivatives | Pyrrole-2-carboxylic acid | Fruit | RC |  | | (Shi et al., 2019) |
| 281 | Amino acid derivatives | S-(5'-Adenosy)-L-homocysteine | Fruit | RC |  | | (Shi et al., 2019) |
| 282 | Amino acid derivatives | S-(methyl)glutathione | Fruit | RC |  | | (Shi et al., 2019) |
| 283 | Amino acids | 2-Aminoadipic acid (L-Homoglutamic acid) | Fruit | RC |  | | (Shi et al., 2019) |
| 284 | Amino acids | Acetylserine | Fruit | RC |  | | (Yang et al., 2020) |
| 285 | Amino acids | Arginine | Fruit | RC |  | | (Yang et al., 2020) |
| 286 | Amino acids | Asparagine | Fruit | RC |  | | (Yang et al., 2020) |
| 287 | Amino acids | Dl-Norvaline | Fruit | RC |  | | (Shi et al., 2019) |
| 288 | Amino acids | Histidine | Fruit | RC |  | | (Yang et al., 2020) |
| 289 | Amino acids | L-(-)-Tyrosine | Fruit | RC |  | | (Shi et al., 2019) |
| 290 | Amino acids | L-(+)-Arginine | Fruit | RC |  | | (Shi et al., 2019) |
| 291 | Amino acids | L-(+)-Lysine | Fruit | RC |  | | (Shi et al., 2019) |
| 292 | Amino acids | L(+)-Ornithine | Fruit | RC |  | | (Shi et al., 2019) |
| 293 | Amino acids | L-Alanine | Fruit | RC |  | | (Shi et al., 2019) |
| 294 | Amino acids | L-Asparagine | Fruit | RC |  | | (Shi et al., 2019) |
| 295 | Amino acids | L-Aspartic acid | Fruit | RC |  | | (Shi et al., 2019) |
| 296 | Amino acids | L-Citrulline | Fruit | RC |  | | (Shi et al., 2019) |
| 297 | Amino acids | L-Cysteine | Fruit | RC |  | | (Shi et al., 2019) |
| 298 | Amino acids | L-Glutamic acid | Fruit | RC |  | | (Shi et al., 2019) |
| 299 | Amino acids | L-Glutamine | Fruit | RC |  | | (Shi et al., 2019) |
| 300 | Amino acids | L-Histidine | Fruit | RC |  | | (Shi et al., 2019) |
| 301 | Amino acids | L-Homocitrulline | Fruit | RC |  | | (Shi et al., 2019) |
| 302 | Amino acids | L-Homocystine | Fruit | RC |  | | (Shi et al., 2019) |
| 303 | Amino acids | L-Homoserine | Fruit | RC |  | | (Shi et al., 2019) |
| 304 | Amino acids | L-Isoleucine | Fruit | RC |  | | (Shi et al., 2019) |
| 305 | Amino acids | L-Leucine | Fruit | RC |  | | (Shi et al., 2019) |
| 306 | Amino acids | L-Methionine | Fruit | RC |  | | (Shi et al., 2019) |
| 307 | Amino acids | L-Phenylalanine | Fruit | RC |  | | (Shi et al., 2019) |
| 308 | Amino acids | L-Proline | Fruit | RC |  | | (Shi et al., 2019) |
| 309 | Amino acids | L-Serine | Fruit | RC |  | | (Shi et al., 2019) |
| 310 | Amino acids | L-Theanine | Fruit | RC |  | | (Shi et al., 2019) |
| 311 | Amino acids | L-Threonine | Fruit | RC |  | | (Shi et al., 2019) |
| 312 | Amino acids | L-Tryptophan | Fruit | RC |  | | (Shi et al., 2019) |
| 313 | Amino acids | L-Tyramine | Fruit | RC |  | | (Shi et al., 2019) |
| 314 | Amino acids | L-Valine | Fruit | RC |  | | (Shi et al., 2019) |
| 315 | Amino acids | Norleucine isomer 1 | Fruit | RC |  | | (Yang et al., 2020) |
| 316 | Amino acids | Norleucine isomer 2 | Fruit | RC |  | | (Yang et al., 2020) |
| 317 | Amino acids | Phenylalanine | Fruit | RC |  | | (Yang et al., 2020) |
| 318 | Amino acids | Proline | Fruit | RC |  | | (Yang et al., 2020) |
| 319 | Amino acids | Tyrosine isomer1 | Fruit | RC |  | | (Yang et al., 2020) |
| 320 | Amino acids | Tyrosine isomer2 | Fruit | RC |  | | (Yang et al., 2020) |
| 321 | Amino acids | Tyrosine isomer3 | Fruit | RC |  | | (Yang et al., 2020) |
| 322 | Anthocyanins | Cyanidin | Fruit | RC |  | | (Shi et al., 2019) |
| 323 | Anthocyanins | Cyanidin 3-O-glucoside (Kuromanin) | Fruit | RC |  | | (Shi et al., 2019) |
| 324 | Anthocyanins | Cyanidin 3-O-glucosyl-malonylglucoside | Fruit | RC |  | | (Shi et al., 2019) |
| 325 | Anthocyanins | Cyanidin 3-O-rutinoside (Keracyanin) | Fruit | RC |  | | (Shi et al., 2019) |
| 326 | Anthocyanins | Cyanidin O-syringic acid | Fruit | RC |  | | (Shi et al., 2019) |
| 327 | Anthocyanins | Delphinidin | Fruit | RC |  | | (Shi et al., 2019) |
| 328 | Anthocyanins | Delphinidin 3-O-glucoside (Mirtillin) | Fruit | RC |  | | (Shi et al., 2019) |
| 329 | Anthocyanins | Delphinidin 3-O-rutinoside (Tulipanin) | Fruit | RC |  | | (Shi et al., 2019) |
| 330 | Anthocyanins | Malvidin 3,5-diglucoside (Malvin) | Fruit | RC |  | | (Shi et al., 2019) |
| 331 | Anthocyanins | Pelargonidin | Fruit | RC |  | | (Shi et al., 2019) |
| 332 | Anthocyanins | Peonidin O-hexoside | Fruit | RC |  | | (Shi et al., 2019) |
| 333 | Anthocyanins | Peonidin O-malonylhexoside | Fruit | RC |  | | (Shi et al., 2019) |
| 334 | Anthocyanins | Rosinidin O-hexoside | Fruit | RC |  | | (Shi et al., 2019) |
| 335 | Benzoic acid derivatives | 2,4-Dihydroxybenzoic acid | Fruit | RC |  | | (Shi et al., 2019) |
| 336 | Benzoic acid derivatives | 2,5-dihydroxy benzoic acid O-hexside | Fruit | RC |  | | (Shi et al., 2019) |
| 337 | Benzoic acid derivatives | 4-Hydroxybenzaldehyde | Fruit | RC |  | | (Shi et al., 2019) |
| 338 | Benzoic acid derivatives | 8-Methyl-2-oxo-4-phenyl-2H-chromen-7-yl 4-(hexyloxy)benzoate | Fruit | RC |  | | (Shi et al., 2019) |
| 339 | Benzoic acid derivatives | Anthranilate O-hexosyl-O-hexoside | Fruit | RC |  | | (Shi et al., 2019) |
| 340 | Benzoic acid derivatives | Anthranilic acid | Fruit | RC |  | | (Shi et al., 2019) |
| 341 | Benzoic acid derivatives | Benzoic acid | Fruit | RC |  | | (Shi et al., 2019) |
| 342 | Benzoic acid derivatives | Gallic acid O-Hexoside | Fruit | RC |  | | (Shi et al., 2019) |
| 343 | Benzoic acid derivatives | Methyl gallate | Fruit | RC |  | | (Shi et al., 2019) |
| 344 | Benzoic acid derivatives | p-Aminobenzoate | Fruit | RC |  | | (Shi et al., 2019) |
| 345 | Benzoic acid derivatives | Syringic acid O-feruloyl-O-hexoside | Fruit | RC |  | | (Shi et al., 2019) |
| 346 | Benzoic acid derivatives | Syringic acid O-glucoside | Fruit | RC |  | | (Shi et al., 2019) |
| 347 | Benzoic acid derivatives | Vanillin | Fruit | RC |  | | (Shi et al., 2019) |
| 348 | Carbohydrates | 2-Deoxyribose 1-phosphate | Fruit | RC |  | | (Shi et al., 2019) |
| 349 | Carbohydrates | D(-)-Threose | Fruit | RC |  | | (Shi et al., 2019) |
| 350 | Carbohydrates | D-(+)-Glucono-1,5-lactone | Fruit | RC |  | | (Shi et al., 2019) |
| 351 | Carbohydrates | D(+)-Glucose | Fruit | RC |  | | (Shi et al., 2019) |
| 352 | Carbohydrates | D(+)-Melezitose | Fruit | RC |  | | (Shi et al., 2019) |
| 353 | Carbohydrates | D(+)-Melezitose O-rhamnoside | Fruit | RC |  | | (Shi et al., 2019) |
| 354 | Carbohydrates | D-(+)-Sucrose | Fruit | RC |  | | (Shi et al., 2019) |
| 355 | Carbohydrates | D-Fructose 6-phosphate | Fruit | RC |  | | (Shi et al., 2019) |
| 356 | Carbohydrates | D-glucoronic acid | Fruit | RC |  | | (Shi et al., 2019) |
| 357 | Carbohydrates | D-Glucose 6-phosphate | Fruit | RC |  | | (Shi et al., 2019) |
| 358 | Carbohydrates | DL-Arabinose | Fruit | RC |  | | (Shi et al., 2019) |
| 359 | Carbohydrates | D-Sedoheptuiose 7-phosphate | Fruit | RC |  | | (Shi et al., 2019) |
| 360 | Carbohydrates | Glucarate O-Phosphoric acid | Fruit | RC |  | | (Shi et al., 2019) |
| 361 | Carbohydrates | Gluconic acid | Fruit | RC |  | | (Shi et al., 2019) |
| 362 | Carbohydrates | Glucosamine | Fruit | RC |  | | (Shi et al., 2019) |
| 363 | Carbohydrates | L-Fucose | Fruit | RC |  | | (Shi et al., 2019) |
| 364 | Carbohydrates | L-Gulonic-γ-lactone | Fruit | RC |  | | (Shi et al., 2019) |
| 365 | Carbohydrates | N-Acetyl-D-glucosamine | Fruit | RC |  | | (Shi et al., 2019) |
| 366 | Carbohydrates | Ribulose-5-phosphate | Fruit | RC |  | | (Shi et al., 2019) |
| 367 | Carbohydrates | Trehalose 6-phosphate | Fruit | RC |  | | (Shi et al., 2019) |
| 368 | Catechin derivatives | (+)-Gallocatechin (GC) | Fruit | RC |  | | (Shi et al., 2019) |
| 369 | Catechin derivatives | Epicatechin gallate (ECG) | Fruit | RC |  | | (Shi et al., 2019) |
| 370 | Catechin derivatives | Epigallate catechin gallate (EGCG) | Fruit | RC |  | | (Shi et al., 2019) |
| 371 | Catechin derivatives | Epigallocatechin (EGC) | Fruit | RC |  | | (Shi et al., 2019) |
| 372 | Catechin derivatives | Gallocatechin-catechin | Fruit | RC |  | | (Shi et al., 2019) |
| 373 | Catechin derivatives | Gallocatechin-gallocatechin | Fruit | RC |  | | (Shi et al., 2019) |
| 374 | Catechin derivatives | L-Epicatechin | Fruit | RC |  | | (Shi et al., 2019) |
| 375 | Catechin derivatives | Protocatechuic acid | Fruit | RC |  | | (Shi et al., 2019) |
| 376 | Catechin derivatives | Protocatechuic acid O-glucoside | Fruit | RC |  | | (Shi et al., 2019) |
| 377 | Catechin derivatives | Protocatechuic aldehyde | Fruit | RC |  | | (Shi et al., 2019) |
| 378 | Cholines | Acetylcholine | Fruit | RC |  | | (Shi et al., 2019) |
| 379 | Cholines | Choline | Fruit | RC |  | | (Shi et al., 2019) |
| 380 | Cholines | Coumaroyl choline | Fruit | RC |  | | (Shi et al., 2019) |
| 381 | Cholines | Feruloylcholine | Fruit | RC |  | | (Shi et al., 2019) |
| 382 | Cholines | Sinapoylcholine | Fruit | RC |  | | (Shi et al., 2019) |
| 383 | Cholines | sn-Glycero-3-phosphocholine | Fruit | RC |  | | (Shi et al., 2019) |
| 384 | Coumarins | 4-hydroxycoumarin di-glucoside | Fruit | RC |  | | (Shi et al., 2019) |
| 385 | Coumarins | 6-Hydroxy-4-methylcoumarin | Fruit | RC |  | | (Shi et al., 2019) |
| 386 | Coumarins | 6-Methoxy-7,8-DihydroxyCoumarin | Fruit | RC |  | | (Shi et al., 2019) |
| 387 | Coumarins | Daphnetin | Fruit | RC |  | | (Shi et al., 2019) |
| 388 | Coumarins | Esculetin (6,7-dihydroxycoumarin) | Fruit | RC |  | | (Shi et al., 2019) |
| 389 | Coumarins | Esculetin O-quinacyl esculetin O-quinic acid | Fruit | RC |  | | (Shi et al., 2019) |
| 390 | Coumarins | Esculin (6,7-Dihydroxycoumarin-6-glucoside) | Fruit | RC |  | | (Shi et al., 2019) |
| 391 | Coumarins | O-Feruloyl 4-hydroxylcoumarin | Fruit | RC |  | | (Shi et al., 2019) |
| 392 | Coumarins | Scoparone | Fruit | RC |  | | (Shi et al., 2019) |
| 393 | Coumarins | Scopoletin (7-Hydroxy-5-methoxycoumarin) | Fruit | RC |  | | (Shi et al., 2019) |
| 394 | Flavanone | 4'-Hydroxy-5,7-dimethoxyflavanone | Fruit | RC |  | | (Shi et al., 2019) |
| 395 | Flavanone | Butein | Fruit | RC |  | | (Shi et al., 2019) |
| 396 | Flavanone | Hesperetin | Fruit | RC |  | | (Shi et al., 2019) |
| 397 | Flavanone | Hesperetin 5-O-glucoside | Fruit | RC |  | | (Shi et al., 2019) |
| 398 | Flavanone | Hesperetin O-Glucuronic acid | Fruit | RC |  | | (Shi et al., 2019) |
| 399 | Flavanone | Hesperetin O-hexosyl-O-hexoside | Fruit | RC |  | | (Shi et al., 2019) |
| 400 | Flavanone | Hesperetin O-malonylhexoside | Fruit | RC |  | | (Shi et al., 2019) |
| 401 | Flavanone | Homoeriodictyol | Fruit | RC |  | | (Shi et al., 2019) |
| 402 | Flavanone | Isosakuranetin (4'-Methylnaringenin) | Fruit | RC |  | | (Shi et al., 2019) |
| 403 | Flavanone | Naringenin | Fruit | RC |  | | (Shi et al., 2019) |
| 404 | Flavanone | Naringenin 7-O-glucoside (Prunin) | Fruit | RC |  | | (Shi et al., 2019) |
| 405 | Flavanone | Naringenin 7-O-neohesperidoside (Naringin) | Fruit | RC |  | | (Shi et al., 2019) |
| 406 | Flavanone | Naringenin chalcone | Fruit | RC |  | | (Shi et al., 2019) |
| 407 | Flavanone | Naringenin O-malonylhexoside | Fruit | RC |  | | (Shi et al., 2019) |
| 408 | Flavanone | Xanthohumol | Fruit | RC |  | | (Shi et al., 2019) |
| 409 | Flavone | 3’,4’,5’-Tricetin O-rutinoside | Fruit | RC |  | | (Shi et al., 2019) |
| 410 | Flavone | Acacetin | Fruit | RC |  | | (Shi et al., 2019) |
| 411 | Flavone | Acacetin O-acetyl hexoside | Fruit | RC |  | | (Shi et al., 2019) |
| 412 | Flavone | Acacetin O-glucuronic acid | Fruit | RC |  | | (Shi et al., 2019) |
| 413 | Flavone | Apigenin 4-O-rhamnoside | Fruit | RC |  | | (Shi et al., 2019) |
| 414 | Flavone | Apigenin 5-O-glucoside | Fruit | RC |  | | (Shi et al., 2019) |
| 415 | Flavone | Apigenin 7-O-glucoside (Cosmosiin) | Fruit | RC |  | | (Shi et al., 2019) |
| 416 | Flavone | Apigenin 7-O-neohesperidoside (Rhoifolin) | Fruit | RC |  | | (Shi et al., 2019) |
| 417 | Flavone | Apigenin 7-rutinoside (Isorhoifolin) | Fruit | RC |  | | (Shi et al., 2019) |
| 418 | Flavone | Apigenin O-hexosyl-O-rutinoside | Fruit | RC |  | | (Shi et al., 2019) |
| 419 | Flavone | Butin | Fruit | RC |  | | (Shi et al., 2019) |
| 420 | Flavone | Chrysoeriol | Fruit | RC |  | | (Shi et al., 2019) |
| 421 | Flavone | Chrysoeriol 5-O-hexoside | Fruit | RC |  | | (Shi et al., 2019) |
| 422 | Flavone | Chrysoeriol 7-O-hexoside | Fruit | RC |  | | (Shi et al., 2019) |
| 423 | Flavone | Chrysoeriol 7-O-rutinoside | Fruit | RC |  | | (Shi et al., 2019) |
| 424 | Flavone | Chrysoeriol O-glucuronic acid-O-hexoside | Fruit | RC |  | | (Shi et al., 2019) |
| 425 | Flavone | Chrysoeriol O-hexosyl-O-hexoside | Fruit | RC |  | | (Shi et al., 2019) |
| 426 | Flavone | Chrysoeriol O-malonylhexoside | Fruit | RC |  | | (Shi et al., 2019) |
| 427 | Flavone | Limocitrin O-hexoside | Fruit | RC |  | | (Shi et al., 2019) |
| 428 | Flavone | Luteolin | Fruit | RC |  | | (Shi et al., 2019) |
| 429 | Flavone | Luteolin 3',7-di-O-glucoside | Fruit | RC |  | | (Shi et al., 2019) |
| 430 | Flavone | Luteolin 7-O-glucoside (Cynaroside) | Fruit | RC |  | | (Shi et al., 2019) |
| 431 | Flavone | Luteolin O-hexosyl-O-hexosyl-O-hexoside | Fruit | RC |  | | (Shi et al., 2019) |
| 432 | Flavone | Luteolin O-hexosyl-O-pentoside | Fruit | RC |  | | (Shi et al., 2019) |
| 433 | Flavone | Nobiletin | Fruit | RC |  | | (Shi et al., 2019) |
| 434 | Flavone | O-methylChrysoeriol 5-O-hexoside | Fruit | RC |  | | (Shi et al., 2019) |
| 435 | Flavone | O-methylChrysoeriol 7-O-hexoside | Fruit | RC |  | | (Shi et al., 2019) |
| 436 | Flavone | sakuranetin | Fruit | RC |  | | (Shi et al., 2019) |
| 437 | Flavone | Syringetin 5-O-hexoside | Fruit | RC |  | | (Shi et al., 2019) |
| 438 | Flavone | Syringetin 7-O-hexoside | Fruit | RC |  | | (Shi et al., 2019) |
| 439 | Flavone | Tangeretin | Fruit | RC |  | | (Shi et al., 2019) |
| 440 | Flavone | Tricetin O-malonylhexoside | Fruit | RC |  | | (Shi et al., 2019) |
| 441 | Flavone | Tricin | Fruit | RC |  | | (Shi et al., 2019) |
| 442 | Flavone | Tricin 5-O-hexoside | Fruit | RC |  | | (Shi et al., 2019) |
| 443 | Flavone | Tricin 5-O-hexosyl-O-hexoside | Fruit | RC |  | | (Shi et al., 2019) |
| 444 | Flavone | Tricin 7-O-acetylglucoside | Fruit | RC |  | | (Shi et al., 2019) |
| 445 | Flavone | Tricin 7-O-hexoside | Fruit | RC |  | | (Shi et al., 2019) |
| 446 | Flavone | Tricin 7-O-hexosyl-O-hexoside | Fruit | RC |  | | (Shi et al., 2019) |
| 447 | Flavone | Tricin O-eudesmic acid | Fruit | RC |  | | (Shi et al., 2019) |
| 448 | Flavone | Tricin O-malonylhexoside | Fruit | RC |  | | (Shi et al., 2019) |
| 449 | Flavone | Tricin O-saccharic acid | Fruit | RC |  | | (Shi et al., 2019) |
| 450 | Flavone C-glycosides | 6-C-hexosyl luteolin O-pentoside | Fruit | RC |  | | (Shi et al., 2019) |
| 451 | Flavone C-glycosides | 6-C-hexosyl-apigenin O-hexosyl-O-hexoside | Fruit | RC |  | | (Shi et al., 2019) |
| 452 | Flavone C-glycosides | 6-C-hexosyl-hesperetin O-hexoside | Fruit | RC |  | | (Shi et al., 2019) |
| 453 | Flavone C-glycosides | 6-C-hexosyl-luteolin O-hexoside | Fruit | RC |  | | (Shi et al., 2019) |
| 454 | Flavone C-glycosides | 8-C-hexosyl-apigenin O-feruloylhexoside | Fruit | RC |  | | (Shi et al., 2019) |
| 455 | Flavone C-glycosides | 8-C-hexosyl-apigenin O-hexosyl-O-hexoside | Fruit | RC |  | | (Shi et al., 2019) |
| 456 | Flavone C-glycosides | 8-C-hexosyl-hesperetin O-hexoside | Fruit | RC |  | | (Shi et al., 2019) |
| 457 | Flavone C-glycosides | 8-C-hexosyl-luteolin O-hexoside | Fruit | RC |  | | (Shi et al., 2019) |
| 458 | Flavone C-glycosides | Apigenin C-glucoside | Fruit | RC |  | | (Shi et al., 2019) |
| 459 | Flavone C-glycosides | C-hexosyl-apigenin O-caffeoylhexoside | Fruit | RC |  | | (Shi et al., 2019) |
| 460 | Flavone C-glycosides | C-hexosyl-apigenin O-p-coumaroylhexoside | Fruit | RC |  | | (Shi et al., 2019) |
| 461 | Flavone C-glycosides | C-hexosyl-chrysin O-feruloylhexoside | Fruit | RC |  | | (Shi et al., 2019) |
| 462 | Flavone C-glycosides | C-hexosyl-chrysoeriol O-sinapoylhexoside | Fruit | RC |  | | (Shi et al., 2019) |
| 463 | Flavone C-glycosides | C-hexosyl-luteolin O-feruloylhexoside | Fruit | RC |  | | (Shi et al., 2019) |
| 464 | Flavone C-glycosides | Chrysoeriol C-hexosyl-O-rhamnoside | Fruit | RC |  | | (Shi et al., 2019) |
| 465 | Flavone C-glycosides | C-pentosyl-chrysoeriol 7-O-feruloylhexoside | Fruit | RC |  | | (Shi et al., 2019) |
| 466 | Flavone C-glycosides | di-C,C-hexosyl-apigenin | Fruit | RC |  | | (Shi et al., 2019) |
| 467 | Flavone C-glycosides | Eriodictiol 6-C-hexoside 8-C-hexoside-O-hexoside | Fruit | RC |  | | (Shi et al., 2019) |
| 468 | Flavone C-glycosides | Eriodictyol C-hexoside | Fruit | RC |  | | (Shi et al., 2019) |
| 469 | Flavone C-glycosides | Hesperetin C-hexosyl-O-hexosyl-O-hexoside | Fruit | RC |  | | (Shi et al., 2019) |
| 470 | Flavone C-glycosides | Isovitexin | Fruit | RC |  | | (Shi et al., 2019) |
| 471 | Flavone C-glycosides | Luteolin 8-C-hexosyl-O-hexoside | Fruit | RC |  | | (Shi et al., 2019) |
| 472 | Flavone C-glycosides | Luteolin C-hexosyl-O-rhamnoside O-hexoside | Fruit | RC |  | | (Shi et al., 2019) |
| 473 | Flavone C-glycosides | Vitexin 2''-O-beta-L-rhamnoside | Fruit | RC |  | | (Shi et al., 2019) |
| 474 | Flavonoids | Isorhamnetin | Fruit | RC |  | | (Yang et al., 2020) |
| 475 | Flavonoids | Isorhamnetin-3-O-rutinoside | Fruit | RC |  | | (Yang et al., 2020) |
| 476 | Flavonoids | Kaempferol | Fruit | RC |  | | (Yang et al., 2020) |
| 477 | Flavonoids | Kaempferol-3-O-rutinoside | Fruit | RC |  | | (Yang et al., 2020) |
| 478 | Flavonoids | Myricetin | Fruit | RC |  | | (Yang et al., 2020) |
| 479 | Flavonoids | Naringenin | Fruit | RC |  | | (Yang et al., 2020) |
| 480 | Flavonoids | Quercetin 3-rutinoside-hexose | Fruit | RC |  | | (Yang et al., 2020) |
| 481 | Flavonoids | Quercetin-3-O-glucoside | Fruit | RC |  | | (Yang et al., 2020) |
| 482 | Flavonoids | Rutin isomer 1 | Fruit | RC |  | | (Yang et al., 2020) |
| 483 | Flavonoids | Rutin isomer 2 | Fruit | RC |  | | (Yang et al., 2020) |
| 484 | Flavonol | Aromadedrin (Dihydrokaempferol) | Fruit | RC |  | | (Shi et al., 2019) |
| 485 | Flavonol | Dihydromyricetin | Fruit | RC |  | | (Shi et al., 2019) |
| 486 | Flavonol | Dihydroquercetin (Taxifolin) | Fruit | RC |  | | (Shi et al., 2019) |
| 487 | Flavonol | Di-O-methylquercetin | Fruit | RC |  | | (Shi et al., 2019) |
| 488 | Flavonol | Fustin | Fruit | RC |  | | (Shi et al., 2019) |
| 489 | Flavonol | Isorhamnetin | Fruit | RC |  | | (Shi et al., 2019) |
| 490 | Flavonol | Isorhamnetin 3-O-neohesperidoside | Fruit | RC |  | | (Shi et al., 2019) |
| 491 | Flavonol | Isorhamnetin 5-O-hexoside | Fruit | RC |  | | (Shi et al., 2019) |
| 492 | Flavonol | Isorhamnetin O-hexoside | Fruit | RC |  | | (Shi et al., 2019) |
| 493 | Flavonol | Kaempferide | Fruit | RC |  | | (Shi et al., 2019) |
| 494 | Flavonol | Kaempferol | Fruit | RC |  | | (Shi et al., 2019) |
| 495 | Flavonol | Kaempferol 3-O-galactoside (Trifolin) | Fruit | RC |  | | (Shi et al., 2019) |
| 496 | Flavonol | Kaempferol 3-O-glucoside (Astragalin) | Fruit | RC |  | | (Shi et al., 2019) |
| 497 | Flavonol | Kaempferol 3-O-rhamnoside (Kaempferin) | Fruit | RC |  | | (Shi et al., 2019) |
| 498 | Flavonol | Kaempferol 3-O-robinobioside (Biorobin) | Fruit | RC |  | | (Shi et al., 2019) |
| 499 | Flavonol | Kaempferol 3-O-rutinoside (Nicotiflorin) | Fruit | RC |  | | (Shi et al., 2019) |
| 500 | Flavonol | Kaempferol-3-O-robinoside-7-O-rhamnoside (Robinin) | Fruit | RC |  | | (Shi et al., 2019) |
| 501 | Flavonol | Kumatakenin | Fruit | RC |  | | (Shi et al., 2019) |
| 502 | Flavonol | methylQuercetin O-hexoside | Fruit | RC |  | | (Shi et al., 2019) |
| 503 | Flavonol | Morin | Fruit | RC |  | | (Shi et al., 2019) |
| 504 | Flavonol | Myricetin | Fruit | RC |  | | (Shi et al., 2019) |
| 505 | Flavonol | Quercetin | Fruit | RC |  | | (Shi et al., 2019) |
| 506 | Flavonol | Quercetin 3-O-glucoside (Isotrifoliin) | Fruit | RC |  | | (Shi et al., 2019) |
| 507 | Flavonol | Quercetin 3-O-rutinoside (Rutin) | Fruit | RC |  | | (Shi et al., 2019) |
| 508 | Flavonol | Quercetin 4'-O-glucoside (Spiraeoside) | Fruit | RC |  | | (Shi et al., 2019) |
| 509 | Flavonol | Quercetin 7-O-rutinoside | Fruit | RC |  | | (Shi et al., 2019) |
| 510 | Flavonol | Syringetin 3-O-hexoside | Fruit | RC |  | | (Shi et al., 2019) |
| 511 | Flavonolignan | Tricin 4'-O-(syringyl alcohol) ether 7-O-hexoside | Fruit | RC |  | | (Shi et al., 2019) |
| 512 | Flavonolignan | Tricin 4'-O-β-guaiacylglycerol | Fruit | RC |  | | (Shi et al., 2019) |
| 513 | Hydroxycinnamic acid derivatives | Caffeic acid | Fruit | RC |  | | (Yang et al., 2020) |
| 514 | Hydroxycinnamic acid derivatives | Chlorogenic acid | Fruit | RC |  | | (Yang et al., 2020) |
| 515 | Hydroxycinnamic acid derivatives | Ferulic acid | Fruit | RC |  | | (Yang et al., 2020) |
| 516 | Hydroxycinnamic acid derivatives | p-coumaric acid | Fruit | RC |  | | (Yang et al., 2020) |
| 517 | Hydroxycinnamic acid derivatives | Sinapinic acid | Fruit | RC |  | | (Yang et al., 2020) |
| 518 | Hydroxycinnamic acid derivatives | Sinapinic acid derivative 1 | Fruit | RC |  | | (Yang et al., 2020) |
| 519 | Hydroxycinnamoyl derivatives | 1-O-beta-D-Glucopyranosyl sinapate | Fruit | RC |  | | (Shi et al., 2019) |
| 520 | Hydroxycinnamoyl derivatives | 3-(4-Hydroxyphenyl)propionic acid | Fruit | RC |  | | (Shi et al., 2019) |
| 521 | Hydroxycinnamoyl derivatives | 3,4-Dimethoxycinnamic acid | Fruit | RC |  | | (Shi et al., 2019) |
| 522 | Hydroxycinnamoyl derivatives | 3-Hydroxy-4-methoxycinnamic acid | Fruit | RC |  | | (Shi et al., 2019) |
| 523 | Hydroxycinnamoyl derivatives | 4-Methoxycinnamic acid | Fruit | RC |  | | (Shi et al., 2019) |
| 524 | Hydroxycinnamoyl derivatives | 6-Hydroxymethylherniarin | Fruit | RC |  | | (Shi et al., 2019) |
| 525 | Hydroxycinnamoyl derivatives | Caffeic acid | Fruit | RC |  | | (Shi et al., 2019) |
| 526 | Hydroxycinnamoyl derivatives | Caffeic acid O-glucoside | Fruit | RC |  | | (Shi et al., 2019) |
| 527 | Hydroxycinnamoyl derivatives | Caffeic aldehyde | Fruit | RC |  | | (Shi et al., 2019) |
| 528 | Hydroxycinnamoyl derivatives | Caftaric acid | Fruit | RC |  | | (Shi et al., 2019) |
| 529 | Hydroxycinnamoyl derivatives | Cinnamic acid | Fruit | RC |  | | (Shi et al., 2019) |
| 530 | Hydroxycinnamoyl derivatives | Coniferin | Fruit | RC |  | | (Shi et al., 2019) |
| 531 | Hydroxycinnamoyl derivatives | Coniferylaldehyde | Fruit | RC |  | | (Shi et al., 2019) |
| 532 | Hydroxycinnamoyl derivatives | Coumarin O-rutinoside | Fruit | RC |  | | (Shi et al., 2019) |
| 533 | Hydroxycinnamoyl derivatives | Disinapoyl hexoside | Fruit | RC |  | | (Shi et al., 2019) |
| 534 | Hydroxycinnamoyl derivatives | Ferulic acid | Fruit | RC |  | | (Shi et al., 2019) |
| 535 | Hydroxycinnamoyl derivatives | Gallic acid O-feruloyl-O-hexosyl-O-hexoside | Fruit | RC |  | | (Shi et al., 2019) |
| 536 | Hydroxycinnamoyl derivatives | Homovanillic acid | Fruit | RC |  | | (Shi et al., 2019) |
| 537 | Hydroxycinnamoyl derivatives | Hydroxy-methoxycinnamate | Fruit | RC |  | | (Shi et al., 2019) |
| 538 | Hydroxycinnamoyl derivatives | Medicarpin | Fruit | RC |  | | (Shi et al., 2019) |
| 539 | Hydroxycinnamoyl derivatives | O-Caffeoyl maltotriose | Fruit | RC |  | | (Shi et al., 2019) |
| 540 | Hydroxycinnamoyl derivatives | p-Coumaraldehyde | Fruit | RC |  | | (Shi et al., 2019) |
| 541 | Hydroxycinnamoyl derivatives | p-Coumaric acid | Fruit | RC |  | | (Shi et al., 2019) |
| 542 | Hydroxycinnamoyl derivatives | p-Coumaryl alcohol | Fruit | RC |  | | (Shi et al., 2019) |
| 543 | Hydroxycinnamoyl derivatives | Pinoresinol | Fruit | RC |  | | (Shi et al., 2019) |
| 544 | Hydroxycinnamoyl derivatives | Resveratrol | Fruit | RC |  | | (Shi et al., 2019) |
| 545 | Hydroxycinnamoyl derivatives | Sinapic acid | Fruit | RC |  | | (Shi et al., 2019) |
| 546 | Hydroxycinnamoyl derivatives | Sinapinaldehyde | Fruit | RC |  | | (Shi et al., 2019) |
| 547 | Hydroxycinnamoyl derivatives | Sinapyl alcohol | Fruit | RC |  | | (Shi et al., 2019) |
| 548 | Hydroxycinnamoyl derivatives | Syringaldehyde | Fruit | RC |  | | (Shi et al., 2019) |
| 549 | Hydroxycinnamoyl derivatives | Syringic acid | Fruit | RC |  | | (Shi et al., 2019) |
|  | Hydroxycinnamoyl derivatives | Syringin | Fruit | RC |  | | (Shi et al., 2019) |
| 551 | Hydroxycinnamoyl derivatives | trans-cinnamaldehyde | Fruit | RC |  | | (Shi et al., 2019) |
| 552 | Hydroxycinnamoyl derivatives | Vanillic acid | Fruit | RC |  | | (Shi et al., 2019) |
| 553 | Indole derivatives | 3-Indoleacetonitrile | Fruit | RC |  | | (Shi et al., 2019) |
| 554 | Indole derivatives | Indole | Fruit | RC |  | | (Shi et al., 2019) |
| 555 | Indole derivatives | Indole-3-carboxaldehyde | Fruit | RC |  | | (Shi et al., 2019) |
| 556 | Indole derivatives | Indole-5-carboxylic acid | Fruit | RC |  | | (Shi et al., 2019) |
| 557 | Indole derivatives | Methoxyindoleacetic acid | Fruit | RC |  | | (Shi et al., 2019) |
| 558 | Isoflavone | 2'-Hydroxygenistein | Fruit | RC |  | | (Shi et al., 2019) |
| 559 | Isoflavone | 6-Hydroxydaidzein | Fruit | RC |  | | (Shi et al., 2019) |
| 560 | Isoflavone | Daidzein | Fruit | RC |  | | (Shi et al., 2019) |
| 561 | Isoflavone | Formononetin (4'-O-methyldaidzein) | Fruit | RC |  | | (Shi et al., 2019) |
| 562 | Isoflavone | Genistein 7-O-Glucoside (Genistin) | Fruit | RC |  | | (Shi et al., 2019) |
| 563 | Isoflavone | Glycitin | Fruit | RC |  | | (Shi et al., 2019) |
| 564 | Lipids_Fatty acids | 12,13-EODE | Fruit | RC |  | | (Shi et al., 2019) |
| 565 | Lipids_Fatty acids | 13-HOTrE(r) | Fruit | RC |  | | (Shi et al., 2019) |
| 566 | Lipids_Fatty acids | 13-HPODE | Fruit | RC |  | | (Shi et al., 2019) |
| 567 | Lipids_Fatty acids | 13-HpOTrE(r) | Fruit | RC |  | | (Shi et al., 2019) |
| 568 | Lipids_Fatty acids | 14,15-Dehydrocrepenynic acid | Fruit | RC |  | | (Shi et al., 2019) |
| 569 | Lipids_Fatty acids | 4-Hydroxysphinganine | Fruit | RC |  | | (Shi et al., 2019) |
| 570 | Lipids_Fatty acids | 8,15-DiHETE | Fruit | RC |  | | (Shi et al., 2019) |
| 571 | Lipids_Fatty acids | 9,10-EODE | Fruit | RC |  | | (Shi et al., 2019) |
| 572 | Lipids_Fatty acids | 9-HOTrE | Fruit | RC |  | | (Shi et al., 2019) |
| 573 | Lipids_Fatty acids | 9-HpOTrE | Fruit | RC |  | | (Shi et al., 2019) |
| 574 | Lipids_Fatty acids | 9-Hydroxy-(10E,12Z,15Z)-octadecatrienoic acid | Fruit | RC |  | | (Shi et al., 2019) |
| 575 | Lipids_Fatty acids | 9-KODE | Fruit | RC |  | | (Shi et al., 2019) |
| 576 | Lipids_Fatty acids | Lauric acid (C12:0) | Fruit | RC |  | | (Shi et al., 2019) |
| 577 | Lipids_Fatty acids | Myristoleic acid (C14:1) | Fruit | RC |  | | (Shi et al., 2019) |
| 578 | Lipids_Fatty acids | Octadeca-11E,13E,15Z-trienoic acid | Fruit | RC |  | | (Shi et al., 2019) |
| 579 | Lipids_Fatty acids | Octadecadien-6-ynoic acid | Fruit | RC |  | | (Shi et al., 2019) |
| 580 | Lipids_Fatty acids | Palmitaldehyde | Fruit | RC |  | | (Shi et al., 2019) |
| 581 | Lipids_Fatty acids | Punicic acid | Fruit | RC |  | | (Shi et al., 2019) |
| 582 | Lipids_Fatty acids | α-Linolenic acid | Fruit | RC |  | | (Shi et al., 2019) |
| 583 | Lipids_Glycerolipids | DGMG (18:1) | Fruit | RC |  | | (Shi et al., 2019) |
| 584 | Lipids_Glycerolipids | DGMG (18:2) isomer1 | Fruit | RC |  | | (Shi et al., 2019) |
| 585 | Lipids_Glycerolipids | DGMG (18:2) isomer2 | Fruit | RC |  | | (Shi et al., 2019) |
| 586 | Lipids_Glycerolipids | DGMG (18:2) isomer3 | Fruit | RC |  | | (Shi et al., 2019) |
| 587 | Lipids_Glycerolipids | MAG (18:1) isomer1 | Fruit | RC |  | | (Shi et al., 2019) |
| 588 | Lipids_Glycerolipids | MAG (18:1) isomer2 | Fruit | RC |  | | (Shi et al., 2019) |
| 589 | Lipids_Glycerolipids | MAG (18:2) | Fruit | RC |  | | (Shi et al., 2019) |
| 590 | Lipids_Glycerolipids | MAG (18:2) isomer1 | Fruit | RC |  | | (Shi et al., 2019) |
| 591 | Lipids_Glycerolipids | MAG (18:3) isomer1 | Fruit | RC |  | | (Shi et al., 2019) |
| 592 | Lipids_Glycerolipids | MAG (18:3) isomer2 | Fruit | RC |  | | (Shi et al., 2019) |
| 593 | Lipids_Glycerolipids | MAG (18:3) isomer3 | Fruit | RC |  | | (Shi et al., 2019) |
| 594 | Lipids_Glycerolipids | MAG (18:3) isomer4 | Fruit | RC |  | | (Shi et al., 2019) |
| 595 | Lipids_Glycerolipids | MAG (18:3) isomer5 | Fruit | RC |  | | (Shi et al., 2019) |
| 596 | Lipids_Glycerolipids | MAG (18:4) isomer2 | Fruit | RC |  | | (Shi et al., 2019) |
| 597 | Lipids_Glycerolipids | MAG (18:4) isomer3 | Fruit | RC |  | | (Shi et al., 2019) |
| 598 | Lipids_Glycerolipids | MGMG (18:2) isomer1 | Fruit | RC |  | | (Shi et al., 2019) |
| 599 | Lipids_Glycerolipids | MGMG (18:2) isomer2 | Fruit | RC |  | | (Shi et al., 2019) |
| 600 | Lipids_Glycerophospholipids | LysoPC 12:1 | Fruit | RC |  | | (Shi et al., 2019) |
| 601 | Lipids_Glycerophospholipids | LysoPC 14:0 | Fruit | RC |  | | (Shi et al., 2019) |
| 602 | Lipids_Glycerophospholipids | LysoPC 14:0 (2n isomer) | Fruit | RC |  | | (Shi et al., 2019) |
| 603 | Lipids_Glycerophospholipids | LysoPC 15:0 | Fruit | RC |  | | (Shi et al., 2019) |
| 604 | Lipids_Glycerophospholipids | LysoPC 15:1 | Fruit | RC |  | | (Shi et al., 2019) |
| 605 | Lipids_Glycerophospholipids | LysoPC 16:0 | Fruit | RC |  | | (Shi et al., 2019) |
| 606 | Lipids_Glycerophospholipids | LysoPC 16:0 (2n isomer) | Fruit | RC |  | | (Shi et al., 2019) |
| 607 | Lipids_Glycerophospholipids | LysoPC 16:1 | Fruit | RC |  | | (Shi et al., 2019) |
| 608 | Lipids_Glycerophospholipids | LysoPC 16:1 (2n isomer) | Fruit | RC |  | | (Shi et al., 2019) |
| 609 | Lipids_Glycerophospholipids | LysoPC 16:2 (2n isomer) | Fruit | RC |  | | (Shi et al., 2019) |
| 610 | Lipids_Glycerophospholipids | LysoPC 17:0 | Fruit | RC |  | | (Shi et al., 2019) |
| 611 | Lipids_Glycerophospholipids | LysoPC 18:0 | Fruit | RC |  | | (Shi et al., 2019) |
| 612 | Lipids_Glycerophospholipids | LysoPC 18:0 (2n isomer) | Fruit | RC |  | | (Shi et al., 2019) |
| 613 | Lipids_Glycerophospholipids | LysoPC 18:1 | Fruit | RC |  | | (Shi et al., 2019) |
| 614 | Lipids_Glycerophospholipids | LysoPC 18:1 (2n isomer) | Fruit | RC |  | | (Shi et al., 2019) |
| 615 | Lipids_Glycerophospholipids | LysoPC 18:2 | Fruit | RC |  | | (Shi et al., 2019) |
| 616 | Lipids_Glycerophospholipids | LysoPC 18:2 (2n isomer) | Fruit | RC |  | | (Shi et al., 2019) |
| 617 | Lipids_Glycerophospholipids | LysoPC 18:3 | Fruit | RC |  | | (Shi et al., 2019) |
| 618 | Lipids_Glycerophospholipids | LysoPC 18:3 (2n isomer) | Fruit | RC |  | | (Shi et al., 2019) |
| 619 | Lipids_Glycerophospholipids | LysoPC 19:0 | Fruit | RC |  | | (Shi et al., 2019) |
| 620 | Lipids_Glycerophospholipids | LysoPC 20:1 | Fruit | RC |  | | (Shi et al., 2019) |
| 621 | Lipids_Glycerophospholipids | LysoPC 20:1 (2n isomer) | Fruit | RC |  | | (Shi et al., 2019) |
| 622 | Lipids_Glycerophospholipids | LysoPC 20:4 | Fruit | RC |  | | (Shi et al., 2019) |
| 623 | Lipids_Glycerophospholipids | LysoPE 14:0 | Fruit | RC |  | | (Shi et al., 2019) |
| 624 | Lipids_Glycerophospholipids | LysoPE 14:0 (2n isomer) | Fruit | RC |  | | (Shi et al., 2019) |
| 625 | Lipids_Glycerophospholipids | LysoPE 16:0 | Fruit | RC |  | | (Shi et al., 2019) |
| 626 | Lipids_Glycerophospholipids | LysoPE 16:0 (2n isomer) | Fruit | RC |  | | (Shi et al., 2019) |
| 627 | Lipids_Glycerophospholipids | LysoPE 18:0 | Fruit | RC |  | | (Shi et al., 2019) |
| 628 | Lipids_Glycerophospholipids | LysoPE 18:0 (2n isomer) | Fruit | RC |  | | (Shi et al., 2019) |
| 629 | Lipids_Glycerophospholipids | LysoPE 18:1 | Fruit | RC |  | | (Shi et al., 2019) |
| 630 | Lipids_Glycerophospholipids | LysoPE 18:1 (2n isomer) | Fruit | RC |  | | (Shi et al., 2019) |
| 631 | Lipids_Glycerophospholipids | LysoPE 18:2 (2n isomer) | Fruit | RC |  | | (Shi et al., 2019) |
| 632 | Lipids_Glycerophospholipids | LysoPE 18:3 | Fruit | RC |  | | (Shi et al., 2019) |
| 633 | Lipids_Glycerophospholipids | PC 19:2/16:0 | Fruit | RC |  | | (Shi et al., 2019) |
| 634 | Nicotinic acid derivatives | 6-hydroxynicotinic acid | Fruit | RC |  | | (Shi et al., 2019) |
| 635 | Nicotinic acid derivatives | Nicotinate ribonucleoside | Fruit | RC |  | | (Shi et al., 2019) |
| 636 | Nicotinic acid derivatives | Nicotinic acid | Fruit | RC |  | | (Shi et al., 2019) |
| 637 | Nicotinic acid derivatives | Nicotinic acid-hexoside | Fruit | RC |  | | (Shi et al., 2019) |
| 638 | Nucleotide and its derivates | 1-Methyladenine | Fruit | RC |  | | (Shi et al., 2019) |
| 639 | Nucleotide and its derivates | 1-Methyladenosine | Fruit | RC |  | | (Shi et al., 2019) |
| 640 | Nucleotide and its derivates | 1-methylguanidine | Fruit | RC |  | | (Shi et al., 2019) |
| 641 | Nucleotide and its derivates | 2-(dimethylamino)guanosine | Fruit | RC |  | | (Shi et al., 2019) |
| 642 | Nucleotide and its derivates | 2'-Deoxyadenosine-5'-monophosphate | Fruit | RC |  | | (Shi et al., 2019) |
| 643 | Nucleotide and its derivates | 2'-Deoxycytidine-5'-monophosphate | Fruit | RC |  | | (Shi et al., 2019) |
| 644 | Nucleotide and its derivates | 2'-Deoxyinosine-5'-monophosphate | Fruit | RC |  | | (Shi et al., 2019) |
| 645 | Nucleotide and its derivates | 5'-Deoxy-5'-(methylthio)adenosine | Fruit | RC |  | | (Shi et al., 2019) |
| 646 | Nucleotide and its derivates | 5-Methylcytosine | Fruit | RC |  | | (Shi et al., 2019) |
| 647 | Nucleotide and its derivates | 5-Methyluridine | Fruit | RC |  | | (Shi et al., 2019) |
| 648 | Nucleotide and its derivates | 6-Methylmercaptopurine | Fruit | RC |  | | (Shi et al., 2019) |
| 649 | Nucleotide and its derivates | 7-methylguanine | Fruit | RC |  | | (Shi et al., 2019) |
| 650 | Nucleotide and its derivates | 8-Hydroxy-2-deoxyguanosine | Fruit | RC |  | | (Shi et al., 2019) |
| 651 | Nucleotide and its derivates | 8-Hydroxyguanosine | Fruit | RC |  | | (Shi et al., 2019) |
| 652 | Nucleotide and its derivates | Adenine | Fruit | RC |  | | (Shi et al., 2019) |
| 653 | Nucleotide and its derivates | Adenosine | Fruit | RC |  | | (Shi et al., 2019) |
| 654 | Nucleotide and its derivates | Adenosine 3'-monophosphate | Fruit | RC |  | | (Shi et al., 2019) |
| 655 | Nucleotide and its derivates | Adenosine 5'-monophosphate | Fruit | RC |  | | (Shi et al., 2019) |
| 656 | Nucleotide and its derivates | Adenosine O-ribose | Fruit | RC |  | | (Shi et al., 2019) |
| 657 | Nucleotide and its derivates | Cyclic AMP | Fruit | RC |  | | (Shi et al., 2019) |
| 658 | Nucleotide and its derivates | Cytidine | Fruit | RC |  | | (Shi et al., 2019) |
| 659 | Nucleotide and its derivates | Cytidine 5'-monophosphate (Cytidylic acid) | Fruit | RC |  | | (Shi et al., 2019) |
| 660 | Nucleotide and its derivates | Cytosine | Fruit | RC |  | | (Shi et al., 2019) |
| 661 | Nucleotide and its derivates | Deoxyadenosine | Fruit | RC |  | | (Shi et al., 2019) |
| 662 | Nucleotide and its derivates | Deoxycytidine | Fruit | RC |  | | (Shi et al., 2019) |
| 663 | Nucleotide and its derivates | Deoxyguanosine | Fruit | RC |  | | (Shi et al., 2019) |
| 664 | Nucleotide and its derivates | Dihydrouracil | Fruit | RC |  | | (Shi et al., 2019) |
| 665 | Nucleotide and its derivates | Flavin adenine dinucleotide (FAD) | Fruit | RC |  | | (Shi et al., 2019) |
| 666 | Nucleotide and its derivates | Guanine | Fruit | RC |  | | (Shi et al., 2019) |
| 667 | Nucleotide and its derivates | Guanosine | Fruit | RC |  | | (Shi et al., 2019) |
| 668 | Nucleotide and its derivates | Guanosine 3',5'-cyclic monophosphate | Fruit | RC |  | | (Shi et al., 2019) |
| 669 | Nucleotide and its derivates | Guanosine 5'-monophosphate | Fruit | RC |  | | (Shi et al., 2019) |
| 670 | Nucleotide and its derivates | Guanosine monophosphate | Fruit | RC |  | | (Shi et al., 2019) |
| 671 | Nucleotide and its derivates | Hypoxanthine | Fruit | RC |  | | (Shi et al., 2019) |
| 672 | Nucleotide and its derivates | Hypoxanthine-9-β-D-arabinofuranoside | Fruit | RC |  | | (Shi et al., 2019) |
| 673 | Nucleotide and its derivates | Inosine | Fruit | RC |  | | (Shi et al., 2019) |
| 674 | Nucleotide and its derivates | Inosine 5'-monophosphate | Fruit | RC |  | | (Shi et al., 2019) |
| 675 | Nucleotide and its derivates | iP7G | Fruit | RC |  | | (Shi et al., 2019) |
| 676 | Nucleotide and its derivates | N2-methylguanosine | Fruit | RC |  | | (Shi et al., 2019) |
| 677 | Nucleotide and its derivates | N6-Succinyl Adenosine | Fruit | RC |  | | (Shi et al., 2019) |
| 678 | Nucleotide and its derivates | Nicotinic acid adenine dinucleotide | Fruit | RC |  | | (Shi et al., 2019) |
| 679 | Nucleotide and its derivates | Purine | Fruit | RC |  | | (Shi et al., 2019) |
| 680 | Nucleotide and its derivates | Succinyladenosine | Fruit | RC |  | | (Shi et al., 2019) |
| 681 | Nucleotide and its derivates | Thymine | Fruit | RC |  | | (Shi et al., 2019) |
| 682 | Nucleotide and its derivates | Uracil | Fruit | RC |  | | (Shi et al., 2019) |
| 683 | Nucleotide and its derivates | Uridine | Fruit | RC |  | | (Shi et al., 2019) |
| 684 | Nucleotide and its derivates | Uridine 5’-diphosphate | Fruit | RC |  | | (Shi et al., 2019) |
| 685 | Nucleotide and its derivates | Uridine 5'-diphospho-D-glucose | Fruit | RC |  | | (Shi et al., 2019) |
| 686 | Nucleotide and its derivates | Uridine 5'-monophosphate | Fruit | RC |  | | (Shi et al., 2019) |
| 687 | Nucleotide and its derivates | Xanthine | Fruit | RC |  | | (Shi et al., 2019) |
| 688 | Nucleotide and its derivates | Xanthosine | Fruit | RC |  | | (Shi et al., 2019) |
| 689 | Nucleotide and its derivates | β-Nicotinamide mononucleotide | Fruit | RC |  | | (Shi et al., 2019) |
| 690 | Nucleotide and its derivates | β-Pseudouridine | Fruit | RC |  | | (Shi et al., 2019) |
| 691 | Organic acids | (3,4-Dimethoxyphenyl) acetic acid | Fruit | RC |  | | (Shi et al., 2019) |
| 692 | Organic acids | (Rs)-Mevalonic acid | Fruit | RC |  | | (Shi et al., 2019) |
| 693 | Organic acids | (S)-(-)-2-Hydroxyisocaproic acid | Fruit | RC |  | | (Shi et al., 2019) |
| 694 | Organic acids | 2-(Formylamino)benzoic acid | Fruit | RC |  | | (Shi et al., 2019) |
| 695 | Organic acids | 2,3-Dihydroxybenzoic acid | Fruit | RC |  | | (Shi et al., 2019) |
| 696 | Organic acids | 2-Aminoethanesulfinic acid | Fruit | RC |  | | (Shi et al., 2019) |
| 697 | Organic acids | 2-Aminoethanesulfonic acid | Fruit | RC |  | | (Shi et al., 2019) |
| 698 | Organic acids | 2-Isopropylmalate | Fruit | RC |  | | (Shi et al., 2019) |
| 699 | Organic acids | 2-Methylglutaric acid | Fruit | RC |  | | (Shi et al., 2019) |
| 700 | Organic acids | 2-Methylsuccinic acid | Fruit | RC |  | | (Shi et al., 2019) |
| 701 | Organic acids | 2-Picolinic acid | Fruit | RC |  | | (Shi et al., 2019) |
| 702 | Organic acids | 3-Hydroxy-3-methyl butyric acid | Fruit | RC |  | | (Shi et al., 2019) |
| 703 | Organic acids | 3-Hydroxybutyrate | Fruit | RC |  | | (Shi et al., 2019) |
| 704 | Organic acids | 3-Hydroxypropanoic acid | Fruit | RC |  | | (Shi et al., 2019) |
| 705 | Organic acids | 4-Acetamidobutyric acid | Fruit | RC |  | | (Shi et al., 2019) |
| 706 | Organic acids | 4-Ethylbenzoic acid | Fruit | RC |  | | (Shi et al., 2019) |
| 707 | Organic acids | 4-Guanidinobutyric acid | Fruit | RC |  | | (Shi et al., 2019) |
| 708 | Organic acids | 4-Hydroxy-2-oxoglutaric acid | Fruit | RC |  | | (Shi et al., 2019) |
| 709 | Organic acids | 4-Hydroxy-3-methoxymandelate | Fruit | RC |  | | (Shi et al., 2019) |
| 710 | Organic acids | 4-Hydroxybenzoic acid | Fruit | RC |  | | (Shi et al., 2019) |
| 711 | Organic acids | 4-Oxopentanoate | Fruit | RC |  | | (Shi et al., 2019) |
| 712 | Organic acids | 5-Aminolevulinate | Fruit | RC |  | | (Shi et al., 2019) |
| 713 | Organic acids | 5-hydroxyhexanoic acid | Fruit | RC |  | | (Shi et al., 2019) |
| 714 | Organic acids | 6-Aminocaproic acid | Fruit | RC |  | | (Shi et al., 2019) |
| 715 | Organic acids | Adipic acid | Fruit | RC |  | | (Shi et al., 2019) |
| 716 | Organic acids | A-Ketoglutaric acid | Fruit | RC |  | | (Shi et al., 2019) |
| 717 | Organic acids | Aminomalonic acid | Fruit | RC |  | | (Shi et al., 2019) |
| 718 | Organic acids | Argininosuccinate | Fruit | RC |  | | (Shi et al., 2019) |
| 719 | Organic acids | Azelaic Acid | Fruit | RC |  | | (Shi et al., 2019) |
| 720 | Organic acids | Citramalate | Fruit | RC |  | | (Shi et al., 2019) |
| 721 | Organic acids | Citric acid | Fruit | RC |  | | (Shi et al., 2019) |
| 722 | Organic acids | Creatine | Fruit | RC |  | | (Shi et al., 2019) |
| 723 | Organic acids | Creatinine | Fruit | RC |  | | (Shi et al., 2019) |
| 724 | Organic acids | D-Erythronolactone | Fruit | RC |  | | (Shi et al., 2019) |
| 725 | Organic acids | Diethyl phosphate | Fruit | RC |  | | (Shi et al., 2019) |
| 726 | Organic acids | Dl-2-Aminooctanoic acid | Fruit | RC |  | | (Shi et al., 2019) |
| 727 | Organic acids | Dodecanedioic aicd | Fruit | RC |  | | (Shi et al., 2019) |
| 728 | Organic acids | D-Pantothenic dcid | Fruit | RC |  | | (Shi et al., 2019) |
| 729 | Organic acids | D-Xylonic acid | Fruit | RC |  | | (Shi et al., 2019) |
| 730 | Organic acids | Ethyl 3,4-Dihydroxybenzoate (Ethyl protocatechuate) | Fruit | RC |  | | (Shi et al., 2019) |
| 731 | Organic acids | Fumaric acid | Fruit | RC |  | | (Shi et al., 2019) |
| 732 | Organic acids | Glutaric acid | Fruit | RC |  | | (Shi et al., 2019) |
| 733 | Organic acids | Guanidinoethyl sulfonate | Fruit | RC |  | | (Shi et al., 2019) |
| 734 | Organic acids | Kynurenic acid | Fruit | RC |  | | (Shi et al., 2019) |
| 735 | Organic acids | Kynurenic acid O-hexside | Fruit | RC |  | | (Shi et al., 2019) |
| 736 | Organic acids | L(-)-Malic acid | Fruit | RC |  | | (Shi et al., 2019) |
| 737 | Organic acids | L-(+)-Tartaric acid | Fruit | RC |  | | (Shi et al., 2019) |
| 738 | Organic acids | Maleic acid | Fruit | RC |  | | (Shi et al., 2019) |
| 739 | Organic acids | Methylglutaric acid | Fruit | RC |  | | (Shi et al., 2019) |
| 740 | Organic acids | Methylmalonic acid | Fruit | RC |  | | (Shi et al., 2019) |
| 741 | Organic acids | Phthalic acid | Fruit | RC |  | | (Shi et al., 2019) |
| 742 | Organic acids | Rosmarinic acid | Fruit | RC |  | | (Shi et al., 2019) |
| 743 | Organic acids | Sebacate | Fruit | RC |  | | (Shi et al., 2019) |
| 744 | Organic acids | Shikimic acid | Fruit | RC |  | | (Shi et al., 2019) |
| 745 | Organic acids | Sinapoyl malate | Fruit | RC |  | | (Shi et al., 2019) |
| 746 | Organic acids | Suberic acid | Fruit | RC |  | | (Shi et al., 2019) |
| 747 | Organic acids | Succinic acid | Fruit | RC |  | | (Shi et al., 2019) |
| 748 | Organic acids | Taurocholic acid | Fruit | RC |  | | (Shi et al., 2019) |
| 749 | Organic acids | Terephthalic acid | Fruit | RC |  | | (Shi et al., 2019) |
| 750 | Organic acids | trans,trans-Muconic acid | Fruit | RC |  | | (Shi et al., 2019) |
| 751 | Organic acids | trans-Citridic acid | Fruit | RC |  | | (Shi et al., 2019) |
| 752 | Organic acids | Xanthurenic acid | Fruit | RC |  | | (Shi et al., 2019) |
| 753 | Organic acids | γ-aminobutyric acid | Fruit | RC |  | | (Shi et al., 2019) |
| 754 | Others | 10-Formyl-THF | Fruit | RC |  | | (Shi et al., 2019) |
| 755 | Others | 2-Aminoethylphosphonate | Fruit | RC |  | | (Shi et al., 2019) |
| 756 | Others | 3-Hydroxypyridine | Fruit | RC |  | | (Shi et al., 2019) |
| 757 | Others | 4-(Aminomethyl)-5-(hydroxymethyl)-2-methylpyridin-3-ol | Fruit | RC |  | | (Shi et al., 2019) |
| 758 | Others | 4-Methyl-5-thiazoleethanol | Fruit | RC |  | | (Shi et al., 2019) |
| 759 | Others | 4-Nitrophenol | Fruit | RC |  | | (Shi et al., 2019) |
| 760 | Others | Aminopurine | Fruit | RC |  | | (Shi et al., 2019) |
| 761 | Others | Arctiin | Fruit | RC |  | | (Shi et al., 2019) |
| 762 | Others | Azoxystrobin acid | Fruit | RC |  | | (Shi et al., 2019) |
| 763 | Others | Benzamide | Fruit | RC |  | | (Shi et al., 2019) |
| 764 | Others | Cholesterol | Fruit | RC |  | | (Shi et al., 2019) |
| 765 | Others | Cocamidopropyl betaine | Fruit | RC |  | | (Shi et al., 2019) |
| 766 | Others | D-erythro-Dihydrosphingosine | Fruit | RC |  | | (Shi et al., 2019) |
| 767 | Others | Diethanolamine | Fruit | RC |  | | (Shi et al., 2019) |
| 768 | Others | DIMBOA glucoside | Fruit | RC |  | | (Shi et al., 2019) |
| 769 | Others | E-3,4,5'-Trihydroxy-3'-glucopyranosylstilbene | Fruit | RC |  | | (Shi et al., 2019) |
| 770 | Others | Ellagic acid | Fruit | RC |  | | (Shi et al., 2019) |
| 771 | Others | Histidinol | Fruit | RC |  | | (Shi et al., 2019) |
| 772 | Others | Hydroxyphenethylamine | Fruit | RC |  | | (Shi et al., 2019) |
| 773 | Others | Inositol | Fruit | RC |  | | (Shi et al., 2019) |
| 774 | Others | Isovitexin 7-O-glucoside (Saponarin) | Fruit | RC |  | | (Shi et al., 2019) |
| 775 | Others | L-Carnitine | Fruit | RC |  | | (Shi et al., 2019) |
| 776 | Others | N-Acetylglucosamine 1-phosphate | Fruit | RC |  | | (Shi et al., 2019) |
| 777 | Others | NADP | Fruit | RC |  | | (Shi et al., 2019) |
| 778 | Others | N-Lauryldiethanolamine | Fruit | RC |  | | (Shi et al., 2019) |
| 779 | Others | O-Phosphorylethanolamine | Fruit | RC |  | | (Shi et al., 2019) |
| 780 | Others | Phellodensin F | Fruit | RC |  | | (Shi et al., 2019) |
| 781 | Others | Phenethylamine | Fruit | RC |  | | (Shi et al., 2019) |
| 782 | Others | Phthalic anhydride | Fruit | RC |  | | (Shi et al., 2019) |
| 783 | Others | sesamolin | Fruit | RC |  | | (Shi et al., 2019) |
| 784 | Phenolamides | Agmatine | Fruit | RC |  | | (Shi et al., 2019) |
| 785 | Phenolamides | N-(4'-O-glycosyl)-feruloyl agmatine | Fruit | RC |  | | (Shi et al., 2019) |
| 786 | Phenolamides | N-(4'-O-glycosyl)-p-coumaroyl agmatine | Fruit | RC |  | | (Shi et al., 2019) |
| 787 | Phenolamides | N', N''-di-p-coumaroylspermidine | Fruit | RC |  | | (Shi et al., 2019) |
| 788 | Phenolamides | N',N",N"'-p-coumaroyl-cinnamoyl-caffeoyl spermidine | Fruit | RC |  | | (Shi et al., 2019) |
| 789 | Phenolamides | N-Acetyl tryptamine | Fruit | RC |  | | (Shi et al., 2019) |
| 790 | Phenolamides | N-Acetylputrescine | Fruit | RC |  | | (Shi et al., 2019) |
| 791 | Phenolamides | N-Caffeoyl agmatine | Fruit | RC |  | | (Shi et al., 2019) |
| 792 | Phenolamides | N-Caffeoyl putrescine | Fruit | RC |  | | (Shi et al., 2019) |
| 793 | Phenolamides | N-Caffeoylspermidine | Fruit | RC |  | | (Shi et al., 2019) |
| 794 | Phenolamides | N-Feruloyl agmatine | Fruit | RC |  | | (Shi et al., 2019) |
| 795 | Phenolamides | N'-Feruloyl putrescine | Fruit | RC |  | | (Shi et al., 2019) |
| 796 | Phenolamides | N-Feruloyl tyramine | Fruit | RC |  | | (Shi et al., 2019) |
| 797 | Phenolamides | N-hexosyl-p-coumaroyl putrescine | Fruit | RC |  | | (Shi et al., 2019) |
| 798 | Phenolamides | N'-p-Coumaroyl agmatine | Fruit | RC |  | | (Shi et al., 2019) |
| 799 | Phenolamides | N-p-Coumaroyl hydroxydehydroagmatine | Fruit | RC |  | | (Shi et al., 2019) |
| 800 | Phenolamides | N'-p-Coumaroyl putrescine | Fruit | RC |  | | (Shi et al., 2019) |
| 801 | Phenolamides | N-p-Coumaroyl putrescine | Fruit | RC |  | | (Shi et al., 2019) |
| 802 | Phenolamides | N-Sinapoyl putrescine | Fruit | RC |  | | (Shi et al., 2019) |
| 803 | Phenolamides | Spermine | Fruit | RC |  | | (Shi et al., 2019) |
| 804 | Proanthocyanidins | Procyanidin A2 | Fruit | RC |  | | (Shi et al., 2019) |
| 805 | Proanthocyanidins | Procyanidin B2 | Fruit | RC |  | | (Shi et al., 2019) |
| 806 | Pyridine derivatives | 1,4-dihydro-1-Methyl-4-oxo-3-pyridinecarboxamide | Fruit | RC |  | | (Shi et al., 2019) |
| 807 | Pyridine derivatives | 3-Carbamyl-1-methylpyridinium (1-Methylnicotinamide) | Fruit | RC |  | | (Shi et al., 2019) |
| 808 | Pyridine derivatives | 4-Pyridoxic acid | Fruit | RC |  | | (Shi et al., 2019) |
| 809 | Quinate and its derivatives | 1-O-Caffeoyl quinic acid | Fruit | RC |  | | (Shi et al., 2019) |
| 810 | Quinate and its derivatives | 1-O-Feruloyl quinic acid | Fruit | RC |  | | (Shi et al., 2019) |
| 811 | Quinate and its derivatives | 3-O-Feruloyl quinic acid | Fruit | RC |  | | (Shi et al., 2019) |
| 812 | Quinate and its derivatives | 3-O-Feruloyl quinic acid glucoside | Fruit | RC |  | | (Shi et al., 2019) |
| 813 | Quinate and its derivatives | 3-O-p-Coumaroyl quinic acid | Fruit | RC |  | | (Shi et al., 2019) |
| 814 | Quinate and its derivatives | 3-O-p-coumaroyl quinic acid O-hexoside | Fruit | RC |  | | (Shi et al., 2019) |
| 815 | Quinate and its derivatives | 3-O-p-Coumaroyl shikimic acid | Fruit | RC |  | | (Shi et al., 2019) |
| 816 | Quinate and its derivatives | 3-O-p-coumaroyl shikimic acid O-hexoside | Fruit | RC |  | | (Shi et al., 2019) |
| 817 | Quinate and its derivatives | 4-O-Caffeoyl quinic acid (criptochlorogenic acid) | Fruit | RC |  | | (Shi et al., 2019) |
| 818 | Quinate and its derivatives | 5-O-Feruloyl quinic acid glucoside | Fruit | RC |  | | (Shi et al., 2019) |
| 819 | Quinate and its derivatives | 5-O-p-coumaroyl quinic acid O-hexoside | Fruit | RC |  | | (Shi et al., 2019) |
| 820 | Quinate and its derivatives | 5-O-p-Coumaroyl shikimic acid | Fruit | RC |  | | (Shi et al., 2019) |
| 821 | Quinate and its derivatives | 5-O-p-coumaroyl shikimic acid O-hexoside | Fruit | RC |  | | (Shi et al., 2019) |
| 822 | Quinate and its derivatives | 5-O-p-Coumaroylquinic acid | Fruit | RC |  | | (Shi et al., 2019) |
| 823 | Quinate and its derivatives | Chlorogenic acid (3-O-Caffeoylquinic acid) | Fruit | RC |  | | (Shi et al., 2019) |
| 824 | Quinate and its derivatives | Chlorogenic acid methyl ester | Fruit | RC |  | | (Shi et al., 2019) |
| 825 | Quinate and its derivatives | Eudesmoyl quinic acid | Fruit | RC |  | | (Shi et al., 2019) |
| 826 | Quinate and its derivatives | Neochlorogenic acid (5-O-Caffeoylquinic acid) | Fruit | RC |  | | (Shi et al., 2019) |
| 827 | Quinate and its derivatives | O-Feruloyl quinic acid | Fruit | RC |  | | (Shi et al., 2019) |
| 828 | Quinate and its derivatives | Quinacyl syringic acid | Fruit | RC |  | | (Shi et al., 2019) |
| 829 | Quinate and its derivatives | Quinic acid | Fruit | RC |  | | (Shi et al., 2019) |
| 830 | Terpenoids | Cucurbitacin D | Fruit | RC |  | | (Shi et al., 2019) |
| 831 | Terpenoids | Phytocassane C | Fruit | RC |  | | (Shi et al., 2019) |
| 832 | Tryptamine derivatives | 5-Methoxy-N,N-dimethyltryptamine | Fruit | RC |  | | (Shi et al., 2019) |
| 833 | Tryptamine derivatives | Cinnamoyl tyramine | Fruit | RC |  | | (Shi et al., 2019) |
| 834 | Tryptamine derivatives | L-Tryptamine | Fruit | RC |  | | (Shi et al., 2019) |
| 835 | Tryptamine derivatives | N-Acetyl-5-hydroxytryptamine | Fruit | RC |  | | (Shi et al., 2019) |
| 836 | Tryptamine derivatives | N-hydroxy tryptamine | Fruit | RC |  | | (Shi et al., 2019) |
| 837 | Tryptamine derivatives | serotonin | Fruit | RC |  | | (Shi et al., 2019) |
| 838 | Vitamins | 4-Pyridoxic acid O-hexoside | Fruit | RC |  | | (Shi et al., 2019) |
| 839 | Vitamins | All-trans-13,14-dihydroretinol | Fruit | RC |  | | (Shi et al., 2019) |
| 840 | Vitamins | Biotin | Fruit | RC |  | | (Shi et al., 2019) |
| 841 | Vitamins | D-Pantothenic acid | Fruit | RC |  | | (Shi et al., 2019) |
| 842 | Vitamins | L-ascorbate | Fruit | RC |  | | (Shi et al., 2019) |
| 843 | Vitamins | Menaquinone (K2) | Fruit | RC |  | | (Shi et al., 2019) |
| 844 | Vitamins | Niacinamide | Fruit | RC |  | | (Shi et al., 2019) |
| 845 | Vitamins | Nicotinic Acid Methyl Ester (Methyl Nicotinate) | Fruit | RC |  | | (Shi et al., 2019) |
| 846 | Vitamins | Orotic acid | Fruit | RC |  | | (Shi et al., 2019) |
| 847 | Vitamins | Pantetheine | Fruit | RC |  | | (Shi et al., 2019) |
| 848 | Vitamins | Pyridoxine | Fruit | RC |  | | (Shi et al., 2019) |
| 849 | Vitamins | Pyridoxine 5'-phosphate | Fruit | RC |  | | (Shi et al., 2019) |
| 850 | Vitamins | Pyridoxine O-glucoside | Fruit | RC |  | | (Shi et al., 2019) |
| 851 | Vitamins | Riboflavin | Fruit | RC |  | | (Shi et al., 2019) |
| 852 | Vitamins | Vitamin D3 | Fruit | RC |  | | (Shi et al., 2019) |
| 853 | MAgnesium porphyrin | chlorophyll content | leaf | RC |  | | (Ma et al., 2021) |
| 854 | glycosides | Apigenin 7-O-hexoside | Leave | RC |  | | (Zhu et al., 2017) |
| 855 | glycosides | Diosmetin-7-O-(6-O-pentosyl_x005f_x0002_rhamnoside) | Leave | RC |  | | (Zhu et al., 2017) |
| 856 | glycosides | Kaempferol-3-O-β-glucoside | Leave | RC |  | | (Zhu et al., 2017) |
| 857 | glycosides | Kaempferol-O-dihexoside | Leave | RC |  | | (Zhu et al., 2017) |
| 858 | glycosides | Kaempferol-O-trihexoside | Leave | RC |  | | (Zhu et al., 2017) |
| 859 | glycosides | Quercetin 3-O-β-glucoside | Leave | RC |  | | (Zhu et al., 2017) |
| 860 | glycosides | Quercetin-3-O-pentosyl_x005f_x0002_rhamnoside | Leave | RC |  | | (Zhu et al., 2017) |
| 861 | glycosides | Quercetin-O-dihexoside | Leave | RC |  | | (Zhu et al., 2017) |
| 862 | phenolic acids | 3,4,5-Tri-O-caffeoylquinic acid | Leave | RC |  | | (Zhu et al., 2017) |
| 863 | phenolic acids | 3-O-p-Coumaroylquinic acid | Leave | RC |  | | (Zhu et al., 2017) |
| 864 | phenolic acids | 4-O-Caffeoyl-5-O-p_x0002_coumaroylquinic acid | Leave | RC |  | | (Zhu et al., 2017) |
| 865 | phenolic acids | 4-O-Feruloyl-5-O-caffeoylquinic acid | Leave | RC |  | | (Zhu et al., 2017) |
| 866 | phenolic acids | 5-O-Feruloylquinnic acid | Leave | RC |  | | (Zhu et al., 2017) |
| 867 | phenolic acids | 5-O-p-Coumaroylquinic acid | Leave | RC |  | | (Zhu et al., 2017) |
| 868 | phenolic acids | Caffeoylquinic acid | Leave | RC |  | | (Zhu et al., 2017) |
| 869 | phenolic acids | Cis-5-O-feruloylquinnic acid | Leave | RC |  | | (Zhu et al., 2017) |
| 870 | phenolic acids | Quinic acid | Leave | RC |  | | (Zhu et al., 2017) |

Abbreviation: AC, authentic content; RC, relative content

**Table S10 Candidate post-harvest traits for QTL mapping**

|  | **Traits** | **Character** | **Description** | **Reference** |
| --- | --- | --- | --- | --- |
| 1 | Ash determination | QN | % | (Oguz and Erdogan, 2016) |
| 2 | color of dried fruit | QL | yellow | (Shi et al., 2012) |
|  |  |  | red |  |
|  |  |  | amaranthine |  |
|  |  |  | dark red |  |
|  |  |  | black |  |
| 3 | Decay index | QN |  | (Ge et al., 2008) & (Ali et al., 2010) |
| 4 | Decay percentage | QN | % | (Ali et al., 2010) |
| 5 | Dehydration time of fresh fruit | QN | h | (Ministry of Agriculture of China, 2013) |
| 6 | dry fruit color | QN | R value | (Yao et al., 2018a) |
|  |  |  | G value |  |
|  |  |  | B value |  |
| 7 | dry fruit length | QN | cm | (Yao et al., 2018a) |
| 8 | dry fruit weight | QN | cm | (Yao et al., 2018a) |
| 9 | dry fruit width | QN | cm | (Yao et al., 2018a) |
| 10 | fruit firmness | QN | N | (Mahfoudhi et al., 2014)  (Ali et al., 2010) |
| 11 | Fruit Moisture ratio | QN | % | (Ge et al., 2008) |
| 12 | Juice rate of fresh fruit | QN | % | (Wang et al., 2016) |
| 13 | pH value | QN |  | (Oguz and Erdogan, 2016) |
| 14 | ratio of dried and fresh fruit | QN |  | (Shi et al., 2012) |
| 15 | Sensory evaluation-flavor | PQ | (score, 0-2)bad | (Mahfoudhi et al., 2014) |
|  |  |  | (score, 3_5)fair |  |
|  |  |  | (score, 6_8)good |  |
|  |  |  | (score, 9)excellent |  |
| 16 | Sensory evaluation-overall acceptability | PQ | (score, 0-2)bad | (Mahfoudhi et al., 2014) |
|  |  |  | (score, 3_5)fair |  |
|  |  |  | (score, 6_8)good |  |
|  |  |  | (score, 9)excellent |  |
| 17 | Sensory evaluation-pulp color | PQ | (score, 0-2)bad | (Mahfoudhi et al., 2014) |
|  |  |  | (score, 3_5)fair |  |
|  |  |  | (score, 6_8)good |  |
|  |  |  | (score, 9)excellent |  |
| 18 | Sensory evaluation-texture | PQ | (score, 0-2)bad | (Mahfoudhi et al., 2014) |
|  |  |  | (score, 3_5) fair |  |
|  |  |  | (score, 6_8) good |  |
|  |  |  | (score,9) excellent |  |
| 19 | taste of fresh fruit | PQ | sweet | (Shi et al., 2012) |
|  |  |  | slightly sweet |  |
|  |  |  | slightly bitter |  |
| 20 | The degree of difficulty in drying | PQ | easy | (Shi et al., 2012) |
|  |  |  | intermediate |  |
|  |  |  | difficult |  |
| 21 | tolerance of storage | PQ | strong | (Shi et al., 2012) |
|  |  |  | intermediate |  |
|  |  |  | weak |  |
| 22 | Water soluble dry matter | QL | % | (Oguz and Erdogan, 2016) |
| 23 | Weight loss percentage | QL | % | (Mahfoudhi et al., 2014) & (Ali et al., 2010) |

Abbreviation: QN, quantitative characteristics; QL, qualitative characteristics; PQ, pseudo-qualitative characteristics.

**References:**

Ai, Y., Sun, Y.N., Liu, L., Yao, F.Y., and Zhang, Y., et al. (2021). Determination of biogenic amines in different parts of lycium barbarum l. By hplc with precolumn dansylation. *Molecules* 26. doi:10.3390/molecules26041046

Ali, A., Maqbool, M., Ramachandran, S., and Alderson, P.G. (2010). Gum arabic as a novel edible coating for enhancing shelf-life and improving postharvest quality of tomato (solanum lycopersicum l.) Fruit. *Postharvest Biology and Technology* 58, 42-47. doi:10.1016/j.postharvbio.2010.05.005

Ali, M.C., Chen, J., Zhang, H., Li, Z., and Zhao, L., et al. (2019). Effective extraction of flavonoids from lycium barbarum l. Fruits by deep eutectic solvents-based ultrasound-assisted extraction. *Talanta* 203, 16-22. doi:https://doi.org/10.1016/j.talanta.2019.05.012

Bai, Y., Pavan, S., Zheng, Z., Zappel, N.F., and Reinstaedler, A., et al. (2008). Naturally occurring broad-spectrum powdery mildew resistance in a central american tomato accession is caused by loss of mlo function. *Molecular Plant-Microbe Interactions* 21, 30-39. doi:10.1094/MPMI-21-1-0030

Belović, M., Pestorić, M., Mastilović, J., and Kevrešan, }. (2012). Identification and selection of the descriptors for establishing a sensory profile of tomato by a multidimensional approach. *Food and Feed Research* 39, 33-40.

Brewer, M., Rodriguez, G., Gonzalo, M.J., Anderson, C., and Lang, L., et al. (2008). Tomato analyzer user manual version 2.2.0.0. *Tomato Analyzer User Manual*, 1-18.

Brewer, M.T., Lang, L., Fujimura, K., Dujmovic, N., and Gray, S., et al. (2006). Development of a controlled vocabulary and software application to analyze fruit shape variation in tomato and other plant species. *Plant Physiology* 141, 15-25. doi:10.1104/pp.106.077867

Cao, Y., Zhao, Y., Zhao, J., Chen, X., and Chen, F. (1999). Vitro screening of resistant variants of lycium barbarum l . To root rot disease. *Acta Phytopathologica Sinica*, 163-168.

Carrillo-López, A., and Yahia, E.M. (2014). Changes in color-related compounds in tomato fruit exocarp and mesocarp during ripening using hplc-apci+-mass spectrometry. *Journal of Food Science and Technology* 51, 2720-2726. doi:10.1007/s13197-012-0782-0

Chen, K.Y., and Tanksley, S.D. (2004). High-resolution mapping and functional analysis of se2.1: a major stigma exsertion quantitative trait locus associated with the evolution from allogamy to autogamy in the genus lycopersicon. *Genetics* 168, 1563-1573. doi:10.1534/genetics.103.022558

Constantino, L.V., Rossetto, L.M., Benassi, M.T., Oliveira, C., and Zeffa, D.M., et al. (2021). Physico-biochemical characterization of mini-tomatoes and internal preference mapping based on consumer acceptance. *Scientia Horticulturae* 282, 110034. doi:10.1016/j.scienta.2021.110034

Doganlar, S., Tanksley, S.D., and Mutschler, M.A. (2000). Identification and molecular mapping of loci controlling fruit ripening time in tomato. *Theoretical and Applied Genetics* 100, 249-255. doi:10.1007/s001220050033

Dong, J.Z., Lu, D.Y., and Wang, Y. (2009). Analysis of flavonoids from leaves of cultivated lycium barbarum l. *Plant Foods for Human Nutrition* 64, 199-204. doi:10.1007/s11130-009-0128-x

Endes, Z., Uslu, N., Özcan, M.M., and Er, F. (2015). Physico-chemical properties, fatty acid composition and mineral contents of goji berry (lycium barbarum l.) Fruit. *Journal of Agroalimentary Processes and Technologies* 21, 36-40.

Fernandez-Pozo, N., Menda, N., Edwards, J.D., Saha, S., and Tecle, I.Y., et al. (2014). The sol genomics network (sgn)—from genotype to phenotype to breeding. *Nucleic Acids Research* 43, D1036-D1041. doi:10.1093/nar/gku1195

Frary, A., Doganlar, S., Daunay, M.C., and Tanksley, S.D. (2003). Qtl analysis of morphological traits in eggplant and implications for conservation of gene function during evolution of solanaceous species. *Theoretical and Applied Genetics* 107, 359-370. doi:10.1007/s00122-003-1257-5

Gautier, H.L.N., Rocci, A., Buret, M., Grasselly, D., and Causse, M. (2005). Fruit load or fruit position alters response to temperature and subsequently cherry tomato quality. *Journal of the Science of Food and Agriculture* 85, 1009-1016. doi:10.1002/jsfa.2060

Ge, Y., Cao, Y., Xu, X., Zhao, J., and Zhang, B. (2008). Study on quality change of fresh fruit of lycium barbarum after harvest. *Northen Horticulture*, 227-229.

Gharezi, M., and Gharezi, M. (2012). Effect of post harvest treatment on stored cherry tomatoes. *Journal of Nutrition & Food Sciences* 02. doi:10.4172/2155-9600.1000157

Gong, H., Rehman, F., Yang, T., Li, Z., and Zeng, S., et al. (2019). Construction of the first high-density genetic map and qtl mapping for photosynthetic traits in lycium barbarum l. *Molecular Breeding* 39, 106. doi:10.1007/s11032-019-1000-9

Gonzalo, M.J., and van der Knaap, E. (2008). A comparative analysis into the genetic bases of morphology in tomato varieties exhibiting elongated fruit shape. *Theoretical and Applied Genetics* 116, 647-656. doi:10.1007/s00122-007-0698-7

Guo, M., Shi, T., Duan, Y., Zhu, J., and Li, J., et al. (2015). Investigation of amino acids in wolfberry fruit (lycium barbarum) by solid-phase extraction and liquid chromatography with precolumn derivatization. *Journal of Food Composition and Analysis* 42, 84-90. doi:https://doi.org/10.1016/j.jfca.2015.03.004

He, N., Yang, X., Jiao, Y., Tian, L., and Zhao, Y. (2012). Characterisation of antioxidant and antiproliferative acidic polysaccharides from chinese wolfberry fruits. *Food Chemistry* 133, 978-989. doi:https://doi.org/10.1016/j.foodchem.2012.02.018

Hernández-Pérez, O.I., Valdez-Aguilar, L.A., Alia-Tejacal, I., Cartmill, A.D., and Cartmill, D.L. (2020). Tomato fruit yield, quality, and nutrient status in response to potassium: calcium balance and electrical conductivity in the nutrient solution. *Journal of Soil Science and Plant Nutrition* 20, 484-492. doi:10.1007/s42729-019-00133-9

Javaria, S., Khan, M.Q., Rahman, H.U., and Bakhsh, I. (2012). Response of tomato yield and post harvest life to potash levels. *Sarhad J. Agric* 28, 227-235.

Jin, Y., Wang, L., and Zhu, Y. (2016). Sdudy of rice varieties resistant to *oulema oryzae*. *Hubei Agricultural Sciences* 55, 1455-1458.

Li, L., Zhao, W., Feng, X., Chen, L., and Zhang, L., et al. (2018). Changes in fruit firmness, cell wall composition, and transcriptional profile in theyellow fruit tomato 1 (*yft1*) mutant. *Journal of Agricultural and Food Chemistry* 67, 463-472. doi:10.1021/acs.jafc.8b04611

Li, Y.Y., Di R, Hsu, W.L., Huang, Y.Q., and Cheung, H.Y. (2017). Quality control of lycium chinense and lycium barbarum cortex (digupi) by hplc using kukoamines as markers. *Chin Med* 12, 4. doi:10.1186/s13020-016-0121-x

Li, Z., Lv, K., Wang, Y., Zhao, B., and Yang, Z. (2015). Multi-scale engineering properties of tomato fruits related to harvesting, simulation and textural evaluation. *Lwt - Food Science and Technology* 61, 444-451. doi:10.1016/j.lwt.2014.12.018

Lin, C. (2016). *Damage difference and resistance of three main cultivated wolfeberry*, Shandong Agricultural University.

López Camelo, A.F., and Gómez, P.A. (2004). Comparison of color indexes for tomato ripening. *Horticultura Brasileira* 22, 534-537.

Ma, Y., Xie, Y., Ha, R., Cao, B., and Song, L. (2021). Effects of elevated co2 on photosynthetic accumulation, sucrose metabolism-related enzymes, and genes identification in goji berry (lycium barbarum l.). *Frontiers in Plant Science* 12. doi:10.3389/fpls.2021.643555

Mahfoudhi, N., Chouaibi, M., and Hamdi, S. (2014). Effectiveness of almond gum trees exudate as a novel edible coating for improving postharvest quality of tomato (*solanum lycopersicum* l.) Fruits. *Food Science and Technology International* 20, 33-43. doi:10.1177/1082013212469617

Maria, J.G., Marin, T.B., Claire, A., David, S., and Simon, G., et al. (2009). Tomato fruit shape analysis using morphometric and morphology attributes implemented in tomato analyzer software program. *Journal of the American Society for Horticultural Science J. Amer. Soc. Hort. Sci.* 134, 77-87. doi:10.21273/JASHS.134.1.77

Mazzucato, A., Ficcadenti, N., Caioni, M., Mosconi, P., and Piccinini, E., et al. (2010). Genetic diversity and distinctiveness in tomato (solanum lycopersicum l.) Landraces: the italian case study of ‘a pera abruzzese’. *Scientia Horticulturae* 125, 55-62. doi:10.1016/j.scienta.2010.02.021

Mendes, K.F., Mendes, K.F., Guedes, S.F., Silva, L.C.A.S., and Arthur, V. (2020). Evaluation of physicochemical characteristics in cherry tomatoes irradiated with 60co gamma-rays on post-harvest conservation. *Radiation Physics and Chemistry* 177, 109139. doi:10.1016/j.radphyschem.2020.109139

Ministry of Agriculture of China (2013). "Guideline for the conduct of tests for distinctness, uniformity and stability — lycium". In: *NY/T 2528-2013* (ed.) Testing of New Varieties of Plants. (Standards Press of China: Beijing).

Nankar, A.N., Tringovska, I., Grozeva, S., Todorova, V., and Kostova, D. (2020). Application of high-throughput phenotyping tool tomato analyzer to characterize balkan capsicum fruit diversity. *Scientia Horticulturae* 260, 108862. doi:10.1016/j.scienta.2019.108862

Nurullayeva, N., Haydarov, K., Umurzakova, Z., and Safarova, D. (2021). Growth and development of lycium barbarum l. In the environment of samarkand in uzbekistan. *Plant Science Today* 8, 278-282. doi:10.14719/pst.2021.8.2.919

Oguz, H.I., and Erdogan, O. (2016). A study on the development performances of goji berry (lycium barbarum l.) Varieties. *Fresenius Environment Bulletin*, 5581.

Pan, C., Yang, D., Zhao, X., Jiao, C., and Yan, Y., et al. (2019). Tomato stigma exsertion induced by high temperature is associated with the jasmonate signalling pathway. *Plant, Cell & Environment* 42, 1205-1221. doi:10.1111/pce.13444

Panthee, D.R., Labate, J.A., McGrath, M.T., Breksa, A.P., and Robertson, L.D. (2013). Genotype and environmental interaction for fruit quality traits in vintage tomato varieties. *Euphytica* 193, 169-182. doi:10.1007/s10681-013-0895-1

Peng, Y., Ma, C., Li, Y., Leung, K.S., and Jiang, Z.H., et al. (2005). Quantification of zeaxanthin dipalmitate and total carotenoids in lycium fruits (fructus lycii). *Plant Foods Hum Nutr* 60, 161-164. doi:10.1007/s11130-005-9550-5

Pestorić, M., Mastilović, J., Kevrešan, }., Pezo, L., and Belović, M., et al. (2021). Artificial neural network model in predicting the quality of fresh tomato genotypes. *Food and Feed Research* 48, 9-21. doi:10.5937/ffr48-29661

Pieczywek, P., Nowacka, M., Dadan, M., Wiktor, A., and Rybak, K., et al. (2018). Postharvest monitoring of tomato ripening using the dynamic laser speckle. *Sensors* 18, 1093. doi:10.3390/s18041093

Qi, Y., Zhu, C., Chen, J., Liu, G., and Yang, Z., et al. (2019). Comparative analysis of the quality and health-promoting compounds of two-shaped fruits of wild lycium ruthenicum murr. From the qinghai–tibet plateau. *Acta Physiologiae Plantarum* 41. doi:10.1007/s11738-019-2888-8

Rehman, F., Gong, H., Li, Z., Zeng, S., and Yang, T., et al. (2020). Identification of fruit size associated quantitative trait loci featuring slaf based high-density linkage map of goji berry (lycium spp.). *Bmc Plant Biology* 20. doi:10.1186/s12870-020-02567-1

Rodríguez, G., Strecker, J., Brewer, M., Anderson, C., and Lang, L., et al. (2010). Tomato analyzer user manual version 3. *Ohaio University*, 1-26.

Rodríguez, G.R., Moyseenko, J.B., Robbins, M.D., Huarachi Morejón, N., and Francis, D.M., et al. (2010). Tomato analyzer: a useful software application to collect accurate and detailed morphological and colorimetric data from two-dimensional objects. *Journal of Visualized Experiments*. doi:10.3791/1856

Ruangrak, E., Du, Y., Htwe, N.M.P.S., Pimorat, P., and Gao, J. (2019). Identification of early tomato fruit ripening loci by qtl-seq. *Journal of Agricultural Science* 11, 51-70.

Sanjaya, S., Pura, M.L., Gusti, S.K., Yanto, F., and Syafria, F. (2019). K-nearest neighbor for classification of tomato maturity level based on hue, saturation, and value colors. *Indonesian Journal of Artificial Intelligence and Data Mining* 2, 101-106.

Schubert, R., Grunewald, S., von Sivers, L., and Hause, B. (2019). Effects of jasmonate on ethylene function during the development of tomato stamens. *Plants* 8, 277. doi:10.3390/plants8080277

Sharma, B. (2012). Correlation and path coefficient analysis for quantitative and qualitative traits for fruit yield and seed yield in tomato genotypes. *Theindian Journal of Horticulture* v. 69, 540-544.

Shi, Z., Men, H., and Du, H. (2012). *Description specification and data standard of lycium germplasm resources*. China Forestry Publishing House: Beijing.

Shi, Z., Wei, F., Wan, R., Li, Y., and Wang, Y., et al. (2019). Impact of nitrogen fertilizer levels on metabolite profiling of the lycium barbarum l. Fruit. *Molecules*

24, 3879.

Solaiman, A.R.M., and Rabbani, M.G. (2006). Effects of n p k s and cow dung on growth and yield of tomato. *Bulletin of the Institute of Tropical Agriculture, Kyushu University* 29, 31-37. doi:10.11189/bita.29.31

Song, F., Li, J., Deng, X., and Zhao, Z. (2018). Effects of different inflatable packaging on storage quality of fresh lycium barbarum under different storage temperatures. *Science and Technology of Food Industry* 39, 270-274, 279.

State Forestry Administration of China (2013). "Guideline for the conduct of tests for distinctness, uniformity and stability — lycium". In: *LY/T 2099-2013* (ed.). (Standards Press of China: Beijing).

Tilahun, S., Park, D.S., Seo, M.H., Hwang, I.G., and Kim, S.H., et al. (2018). Prediction of lycopene and β-carotene in tomatoes by portable chroma-meter and vis/nir spectra. *Postharvest Biology and Technology* 136, 50-56. doi:10.1016/j.postharvbio.2017.10.007

Wang, C.C., Chang, S.C., Inbaraj, B.S., and Chen, B.H. (2010). Isolation of carotenoids, flavonoids and polysaccharides from lycium barbarum l. And evaluation of antioxidant activity. *Food Chemistry* 120, 184-192. doi:10.1016/j.foodchem.2009.10.005

Wang, P., Mu, X., Gao, Y.G., Zhang, J., and Du, J. (2020). Successful induction and the systematic characterization of tetraploids in cerasus humilis for subsequent breeding. *Scientia Horticulturae* 265, 109216. doi:10.1016/j.scienta.2020.109216

Wang, Y., Guo, S., An, W., Liu, L., and Yin, Y., et al. (2016). Study on the characters and major constituents in the fruits of five wolfberry varieties. *Journal of Forest and Environment* 36, 367-372.

Wei, A.N., Ya-jun, A.W., En-ning, J., Hao-xia, L.I., and Zhi-gang, S., et al. (2008). Evaluation criteria of some botanical quantitative characteristics of wolfberry (*lycium* l.) Germplasm resources. *Acta Horticulturae Sinica* 35, 301-306.

Weingerl, V., and Unuk, T. (2015). Chemical and fruit skin colour markers for simple quality control of tomato fruits. *Croatian Journal of Food Science and Technology* 7, 76-85. doi:10.17508/CJFST.2015.7.2.03

Wu, J., Ji, J., Wang, G., Wu, G., and Diao, J., et al. (2015). Ectopic expression of the lycium barbarum β-carotene hydroxylase gene (chyb) enhances drought and salt stress resistance by increasing xanthophyll cycle pool in tobacco. *Plant Cell, Tissue and Organ Culture (Pctoc)* 121, 559-569. doi:10.1007/s11240-015-0725-3

Wu, T., Lv, H., Wang, F., and Wang, Y. (2016). Characterization of polyphenols from lycium ruthenicum fruit by uplc-q-tof/mse and their antioxidant activity in caco-2 cells. *Journal of Agricultural and Food Chemistry* 64, 2280-2288. doi:10.1021/acs.jafc.6b00035

Xu, C., Liu, S., Qiao, H., Chen, J., and Guo, K., et al. (2013). Effects of bionic gum on population dynamics of lycium barbarum and its natural enemies. *China Journal of Chinese Materia Medica* 38, 666-669.

Yang, X., Lin, S., Jia, Y., Rehman, F., and Zeng, S., et al. (2020). Anthocyanin and spermidine derivative hexoses coordinately increase in the ripening fruit of lycium ruthenicum. *Food Chemistry* 311, 125874. doi:10.1016/j.foodchem.2019.125874

Yao, R., Heinrich, M., Zou, Y., Reich, E., and Zhang, X., et al. (2018a). Quality variation of goji (fruits of lycium spp.) In china: a comparative morphological and metabolomic analysis. *Frontiers in Pharmacology* 9. doi:10.3389/fphar.2018.00151

Yao, R., Heinrich, M., Zou, Y., Reich, E., and Zhang, X., et al. (2018b). Quality variation of goji (fruits of lycium spp.) In china: a comparative morphological and metabolomic analysis. *Frontiers in Pharmacology* 9. doi:10.3389/fphar.2018.00151

Yu, X., Gao, Y., Zhao, Z., and Gao, J. (2017). Rapid determination of amino acids in chinese wolfberry (lycium bararum l.) Fruit by using fourier transform infrared spectroscopy and partial least square regression. *Food Analytical Methods* 10, 2436-2443. doi:10.1007/s12161-017-0802-9

Zhang, H., Liu, F., Wang, J., Yang, Q., and Wang, P., et al. (2021). Salicylic acid inhibits the postharvest decay of goji berry (lycium barbarum l.) By modulating the antioxidant system and phenylpropanoid metabolites. *Postharvest Biology and Technology* 178, 111558. doi:https://doi.org/10.1016/j.postharvbio.2021.111558

Zhao, J., Li, H., Xi, W., An, W., and Niu, L., et al. (2015). Changes in sugars and organic acids in wolfberry (lycium barbarum l.) Fruit during development and maturation. *Food Chemistry* 173, 718-724. doi:10.1016/j.foodchem.2014.10.082

Zhao, S., Tuan, P.A., Li, X., Kim, Y.B., and Kim, H., et al. (2013). Identification of phenylpropanoid biosynthetic genes and phenylpropanoid accumulation by transcriptome analysis of lycium chinense. *Bmc Genomics* 14, 802. doi:10.1186/1471-2164-14-802

Zhu, L., Peng, Z., Zhang, X., Yang, J., and Lai, X., et al. (2017). Determination of polyphenols in lycium barbarum leaves by high-performance liquid chromatography-tandem mass spectrometry. *Analytical Letters* 50, 761-776. doi:10.1080/00032719.2016.1202956
